# A Unique and Stable Polyproline I Helix Sorted out from Conformational Equilibrium by Solvent Polarity

**DOI:** 10.1021/acs.joc.2c01377

**Published:** 2022-10-15

**Authors:** Matteo Pollastrini, Luca Pasquinelli, Marcin Górecki, Federica Balzano, Lorenzo Cupellini, Filippo Lipparini, Gloria Uccello Barretta, Fabio Marchetti, Gennaro Pescitelli, Gaetano Angelici

**Affiliations:** † Dipartimento di Chimica e Chimica Industriale, Università di Pisa, Via G. Moruzzi 13, Pisa 56124, Italy; ‡ Institute of Organic Chemistry, Polish Academy of Sciences, ul. Kasprzaka 44/52, Warsaw 01-224, Poland

## Abstract

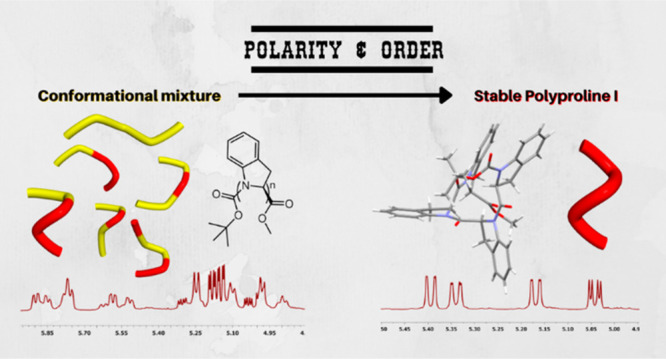

Polyproline I helical structures are often considered as the hidden face of their most famous geminal sibling, Polyproline II, as PPI is generally spotted only within a conformational equilibrium. We designed and synthesized a stable Polyproline I structure exploiting the striking tendency of (*S*)-indoline-2-carboxylic acid to drive the peptide bond conformation toward the *cis* amide isomer, when dissolved in polar solvents. The cooperative effect of only four amino acidic units is sufficient to form a preferential structure in solution. We shed light on this rare secondary structure with a thorough analysis of the spectroscopic and chiroptical properties of the tetramer, supported by X-ray crystallography and computational studies.

## Introduction

Since the very first synthesis of poly-l-proline in 1954, by Ephraim Katchalski-Katzir and collaborators,^1−3^ the conformational behavior of proline-containing oligomers intrigued many scientists. Two recurring helical secondary structures are generally observed with proline and proline-mimetic oligomers: the right-handed polyproline I (PPI) and the left-handed polyproline II (PPII).^4−6^ The characteristic backbone dihedral angles and geometric properties of PPI and PPII are summarized in Table 1.

**Table 1:** Geometrical Properties of Generic Polyprolines I and II

	structure	
	Polyproline I	Polyproline II
helix	right-handed	left-handed
rise per residue	1.90 Å	3.20 Å
residues per turn	3.3	3.0
ω	0°	180°
ϕ	–75°	–75°
ψ	+160	+145

The dualistic and interdependent nature of these two structures lies mainly in the different orientation of the amide bonds, with all amide bonds in the *cis* conformation (ω = 0°) for PPI and all in the *trans* (ω = 180°) for PPII. The PPII helix has been deeply studied,^7−10^ as it is the dominating form in aqueous solution for proline-enriched oligomers, it is involved in many biological processes, and it is the helical conformation adopted by the single strands of collagen.^11−13^ It is worth mentioning the recent advances on the comprehension of the factors, which stabilize PPII over PPI structures: the influence of concentration and of the polarity of the solvent,^14,15^ the effect of terminal functional groups or substituents on the proline moieties,^16−20^ and the temperature and pH.^21−23^

However, the PPI helix is a rare secondary structure, mostly observed in organic solvents in its dichotomic relation with PPII, through the mutarotation phenomenon of oligoprolines. More insight into the synthesis and characterization of PPI helices came from the study of peptoids, *N*-substituted glycine oligomers.^24−26^ In particular, the group of Taillefumier described PPI-like structures, stabilized by sterically hindered aliphatic side chains, which lock the amidic bond exclusively in the *cis* conformation.^27−29^ Another example is the stabilization of a PPI-like peptoid helix through metal coordination by Maayan et al.^30^ Recently, the group of Tedesco crystallized enantiomorphic right- and left-handed polyproline type I helices, obtained by conformationally restricted cyclic dodecapeptoids.^31^

**Scheme 1:**
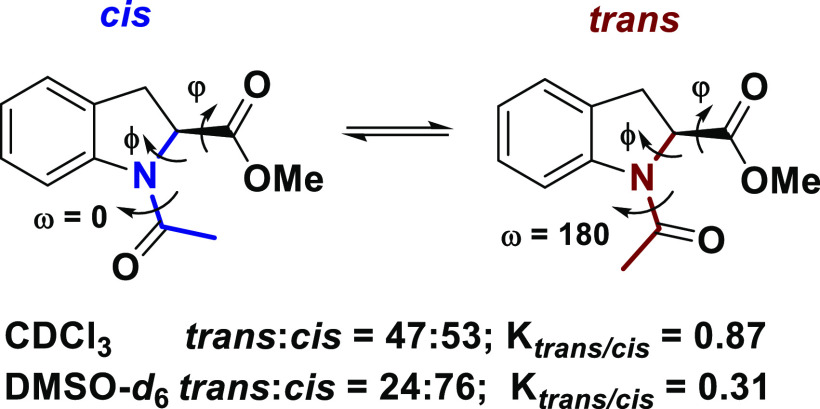
Representation of the *Cis–Trans* Isomerization Equilibrium of the Amidic Bond in Ac-(2*S*)-Ind-OMe Depending on the Polarity of the Solvent^32^

We have recently described the preference of (*S*)-indoline-2-carboxylic acid (2-(*S*)-Ind) derivatives, to drive the peptide bond conformation toward the *cis* amide isomer, when dissolved in polar solvents (Scheme 1).^32^

A thorough experimental and computational study on the conformational properties of the simple acetamide derivative of 2-(*S*)-Ind demonstrated the influence of the molecular dipole moment on the conformational equilibrium, and that steric and electrostatic interactions contribute to stabilize the amide *cis* geometry. 2-(*S*)-Ind is an excellent model molecule because the NMR signals of the different conformers are well separated and defined, allowing a careful measurement of the thermodynamic and kinetic constants for the *cis/trans* isomerism. Moreover, the behavior of 2-(*S*)-Ind is opposite to the general preference of proline for the *trans* isomer, in polar solvents.^33 −35^ Therefore, we speculated that longer oligomers of 2-(*S*)-Ind might show a cooperative effect strong enough to stabilize a PPI helix structure, even with a few amino acidic units. Here, we report the synthesis and structural characterization of oligomers containing one to four units of 2-(*S*)-Ind and prove that the tetramer indeed adopts a PPI conformation both in the solid state and in solution.

## Results and Discussion

### Synthesis of Oligomers **1**–**4**

**Scheme 2:**
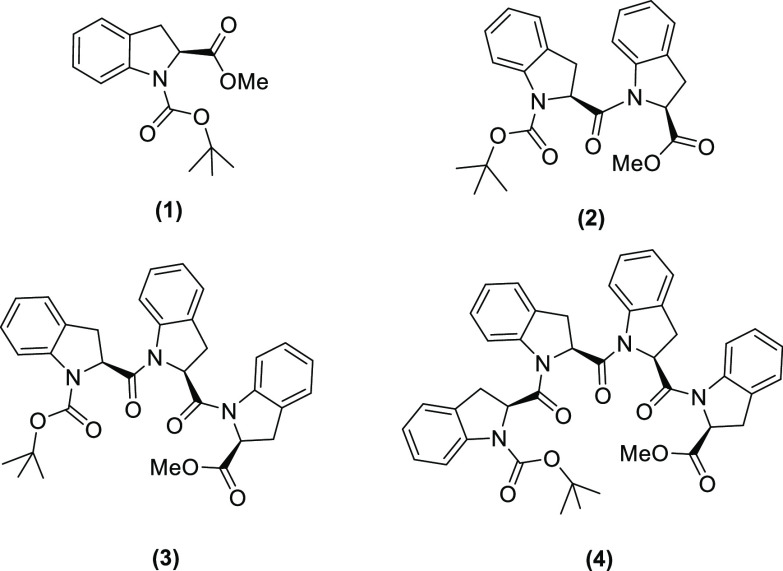
Structures of the Boc-((2*S*)-Ind)_*n*_-OMe Oligomers with *n* = 1–4

**Scheme 3:**
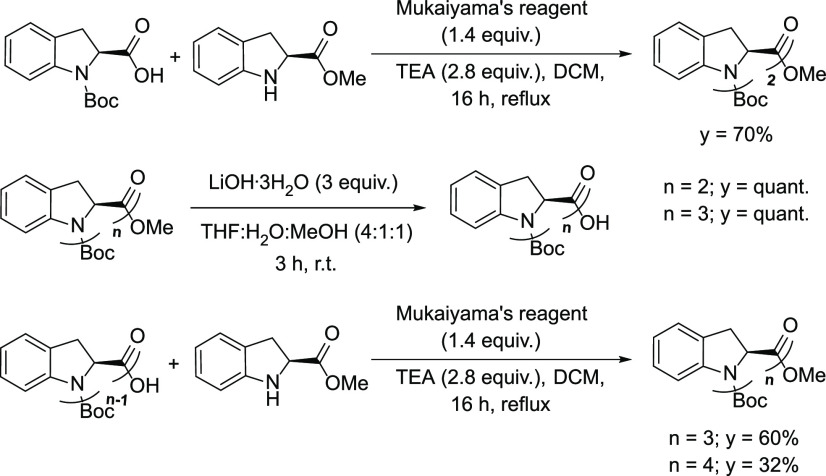
Synthetic Scheme of the Four Oligomers Boc-((2*S*)-Ind)*_n_*-OMe with *n* = 1–4

In our previous work, we studied the conformational preference of the monomeric unit Ac-(2*S*)-Ind-OMe, with the amine protected with a simple acetyl group, for a clear and simple assignment of NMR signals. However, for the synthesis of longer oligomers, we chose the more versatile *t*-butyl carbamate protective group (Boc), which we expected to increase the solubility of the compounds. The planned molecules are summarized in Scheme 2.

(*S*)-Indoline-2-carboxylic acid contains an aromatic secondary amine, which might be too weak, as a nucleophile, for an efficient peptide coupling. Therefore, we optimized the reaction between Boc-(2*S*)-Ind-OH and H-(2*S*)-Ind-OMe by testing different conditions. Interestingly, while the most common peptide coupling reagents, such as HATU, HBTU, FDPP, DCC, and EDCI, did not work at all, Mukaiyama’s reagent (2-chloro-1-methylpyridinium iodide) was efficient, in the conditions described in Scheme 3, although **4** was isolated in modest yield because of solubility issues.

### NMR Experiments

**Figure 1:**
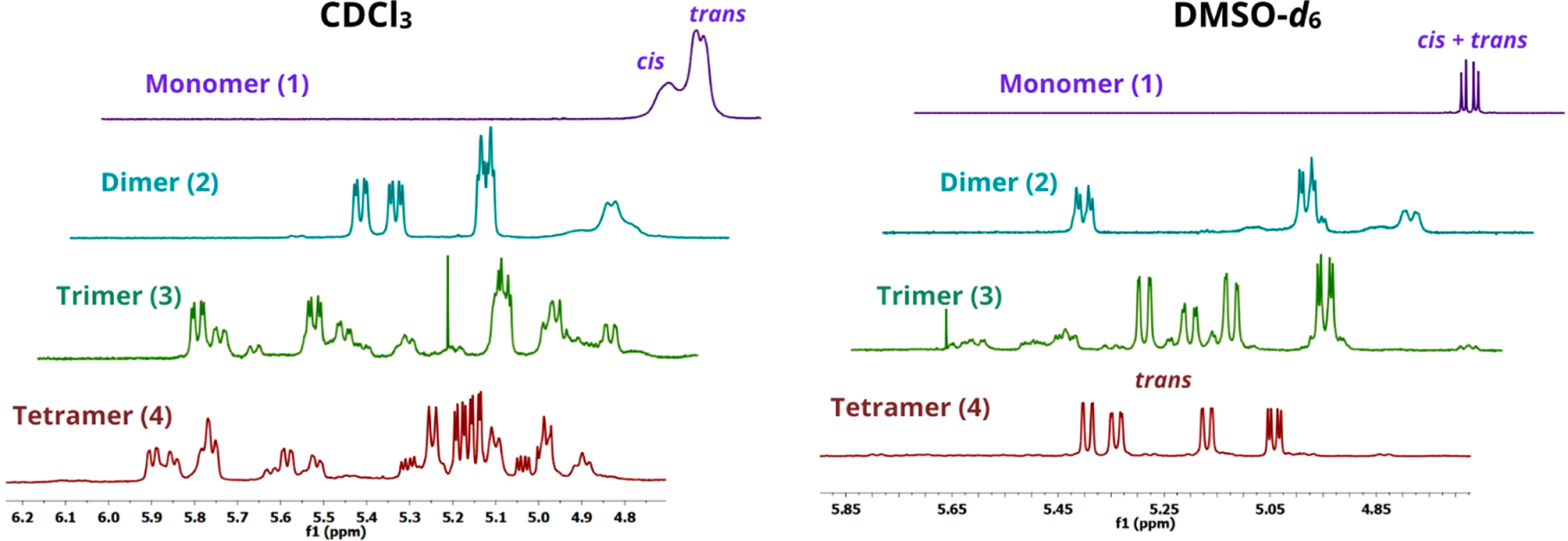
(Left) ^1^H NMR spectra in the H_α_ region of 0.1 M solutions of **1**–**4** oligomers dissolved in CDCl_3_ at 25 °C. (Right) ^1^H NMR spectra in the H_α_ region of 0.1 M solutions of **1**–**4** oligomers dissolved in DMSO-*d*_6_ at 25 °C. Horizontal offset was added for clarity.

We performed ^1^H NMR experiments on the four oligomers (**1**–**4**), using CDCl_3_ (ε_r_ = 4.8) and DMSO-*d*_6_ (ε_r_ = 46.7) as solvents, with a significantly different polarity. Boc-((2*S*)-Ind)*_n_*-OMe angles ϕ are fixed by the pyrrolidine ring; thus, the main degrees of conformational freedom are represented by the torsional angles ω and ψ (see Scheme 1 for angle definition). The amide angle ω is expected to assume values around 0° and 180° leading to *cis* and *trans* isomers, respectively, distinguished by NMR, while the rotational barrier for angle ψ is too low to be recognized by NMR. Therefore, it is reasonable to expect 2^*n*^ possible sets of NMR signals corresponding to the number *n* of amide bonds of the oligomers. Particularly, the regions of the spectra between 4.1 and 5.2 ppm, corresponding to the H_α_ signals, are explicative of the conformational equilibrium. Figure 1 (left) shows, as expected, an increase of complexity for the H_α_ signals of the chemically pure oligomers dissolved in CDCl_3_, upon increasing the oligomer size, indicative of the concurring presence of multiple helices. Conversely, Figure 1 (right) shows a major population of *cis* isomers in oligomers **1**–**3**, when dissolved in DMSO-*d*_6_. Moreover, the ^1^H NMR spectrum at 25 °C of tetramer **4** shows the presence of one predominant fold, suggesting that a cooperative effect stabilizes the formation of a well-defined conformation, possibly a PPI helix, already at the level of the tetramer. To demonstrate the effective prevalence of a single helical structure in solution, we performed more refined NMR experiments (described below) comparing the results with the crystal structure of the tetramer.

### X-ray Crystal Structure of Tetramer **4**

**Figure 2:**
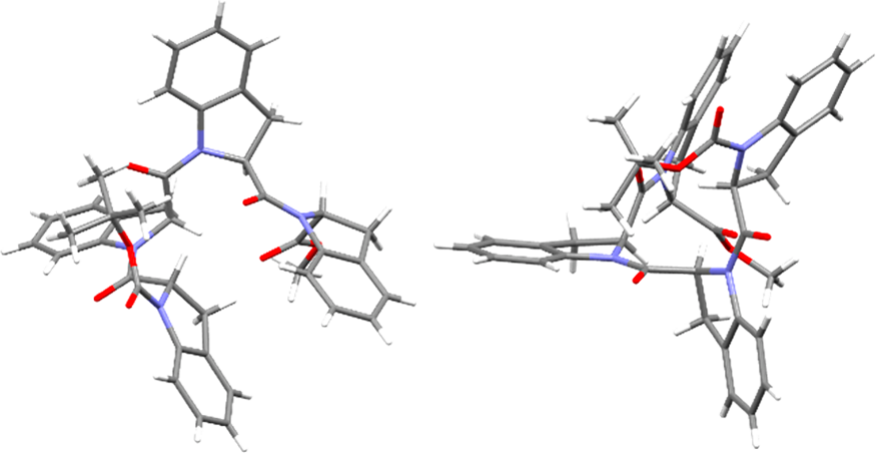
X-ray diffraction molecular structure of **4** (two views). CCDC number: 2173109.

Crystals of tetramer **4** suitable for X-ray diffraction analysis were obtained by slow evaporation of a 0.1 M solution of **4** dissolved in ethyl acetate, a polar solvent. The diffraction pattern showed a 2/*m* symmetry. The unit cell metrics and systematic absences along with the chiroptical properties of the molecule indicate the *P*2_1_ space group. The more relevant crystal data and structure refinement parameters are listed in Table S1, while a representation of the crystal structure of **4** is shown in Figure 2.

The dihedral angle values measured on the crystal are reported in Table 2, showing the typical values of a polyproline I helix. As a crystal structure could represent only one of the possible helices in solution, we performed afterward a series of NMR and CD analyses in solution, to confirm the predominant presence of a PPI helix in polar solvents.

**Table 2:** Dihedral Angle Values Found for Each Residue of Tetramer **4**

residue^a^	ω	ϕ	ψ
A	–0.1	–74.5	165.9
B	–7.9	–85.9	165.5
C	–9.7	–70.8	166.6
D	–7.5	–77.8	170.4^b^

a Residues depicted in Figure 3.

b The ψ angle referred to the ester bond NCCO(Me) of the last residue D.

### Two-Dimensional NMR Analysis

In DMSO-*d*_6_ solution, a largely prevalent species of Boc-((2*S*)-Ind)_4_-OMe (**4**) (>86%) was detected, which was completely characterized by comparing scalar and dipolar interactions detected in 2D homo- and heteronuclear maps. An exhaustive description of the methodology used for the attribution of each signal is reported in the Experimental Section.

For the definition of the conformation of the prevailing species of the tetramer in DMSO-*d*_6_ solution, interunit ROEs were fundamental. As a matter of fact, the H_α_^A^ of the terminal unit produced dipolar interactions nearly exclusively with the H_α_^B^ of the adjacent unit and not with its aromatic protons. In turn, the proton H_α_^B^ gave ROEs on H_α_^A^ and H_α_^C^ with the former more intense than the latter, to indicate that the internuclear distance H_α_^B^–H_α_^A^ was shorter than H_α_^B^–H_α_^C^. Similarly, H_α_^C^ gave almost equivalent ROEs on H_α_^B^ and H_α_^D^. Finally, the proton of the other terminal unit, H_α_^D^, produced a dipolar interaction exclusively with H_α_^C^. The above ROE data (Figure 3) supported an all-*cis* conformation of the tetramer, which is in fact the sole conformation keeping the CH_α_ methine protons in spatial proximity to each other. Such a conformation was also supported by the following spatial proximity constraints imposed by ROE data and represented in Figure 3: (i) intraunit ROE H_α_–H_β(*cis*)_ and interunit ROE H_α_–H_β′(*trans*)*_ (the star denotes the adjacent previous unit) showed comparable intensities (Figure S1); (ii) the methyl moieties of the *t*-butyl group gave ROE on peri aromatic protons H_B1_ and H_C1_ of units B and C; and (iii) a ROE between the protons of the two terminal units H_α_^A^ and H_D1_ was also detected. The conformation of the tetramer in DMSO-*d*_6_ solution defined by NMR data is in very good agreement with that obtained in the solid state by X-ray, as shown in Figure 3. Thus, the PPI helix is maintained in DMSO solution.

**Figure 3:**
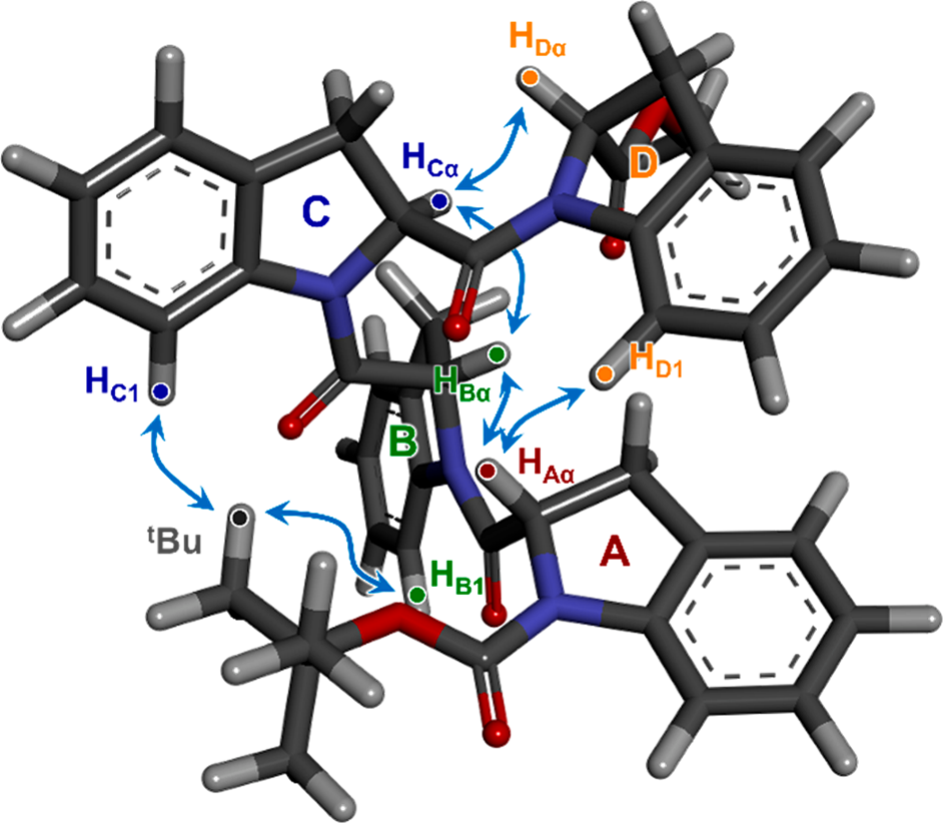
Relevant ROE interactions of **4** shown on the DFT-optimized X-ray structure. For clarity, the H_α_–H_β(*cis*)_ and interunit H_α_–H_β′(*trans*)_ interactions are shown in Figure S1.

### Thermal Stability of Tetramer **4** in DMSO

**Figure 4:**
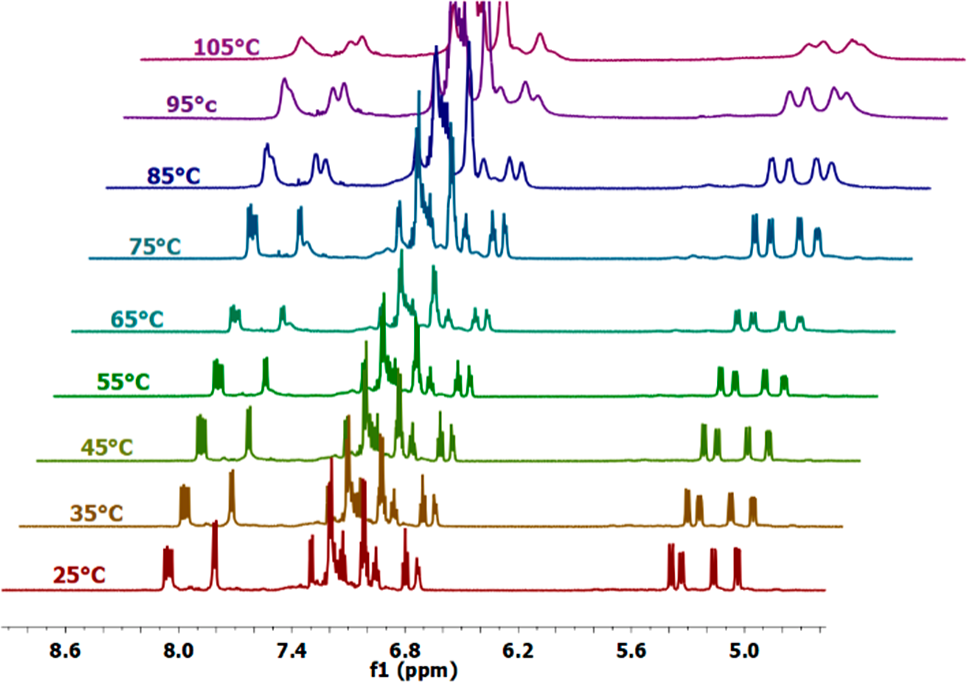
^1^H NMR spectra of a 0.1 M solution of **4** in DMSO-*d*_6_ at different temperatures in the regions of H_α_ protons (5.0–5.6 ppm) and aromatic protons (6.7–8.2 ppm).

We measured ^1^H NMR spectra at variable temperatures of tetramer **4** dissolved in DMSO-*d*_6_, to check the stability in solution of the PPI conformation. The temperature was increased by 10 °C steps for each measurement, starting from 25 until 105 °C. Figure 4 highlights an exceptionally stable structure up to the highest temperature. No other signals, corresponding to other isomers, are visible at higher temperatures in the H_α_ protons region (5.0–5.6 ppm), while the ratio between their integrals and the integrals of peri aromatic H_1_ protons (7.75–8.20 ppm), characteristic of *cis* isomeric units, remains basically unchanged.

### ECD and VCD Spectra of **1**–**4**

**Figure 5:**
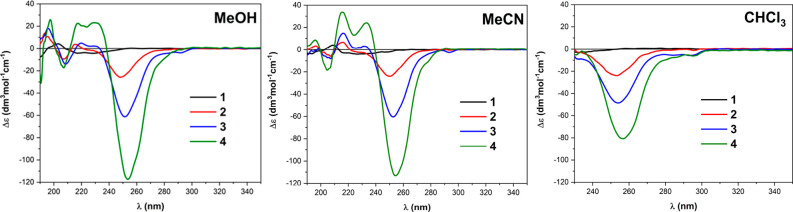
ECD spectra of ca. 3.45 × 10^–4^ M solutions of **1**–**4** in various solvents; cell path lengths are 0.02, 0.05, and 0.2 cm.

Electronic and vibrational circular dichroism (ECD and VCD) spectra were recorded for all compounds **1**–**4** in several solvents, CH_3_OH, CH_3_CN, and CHCl_3_ for ECD (DMSO could not be used due to its absorption bands up to 265 nm), and CD_3_CN, CDCl_3_, and DMSO-*d*_6_ for VCD. The ECD spectrum of monomer **1** is faintly visible. Upon increasing the oligomer length *n*, a consistent structured spectrum appears in all solvents, reaching maximum intensity for tetramer **4** (Figure 5). The most intense negative band at 250–255 nm is absent for **1** and approaches Δε_max_ values of −24, −60, and −113 M^–1^ cm^–1^ for **2**, **3**, and **4**, respectively. The nonlinear increase with *n* witnesses a progressive stabilization of a well-defined conformation, which is similar in CH_3_OH and CH_3_CN, and possibly also in CHCl_3_. In comparison, an almost linear trend is observed for the UV spectra (Figure S2). Variable-temperature ECD spectra measured in CH_3_CN between 20 and 50 °C showed only a modest decrease in intensity upon heating (Figure S3), witnessing again the stability of the major conformation responsible for the ECD profile. Because of the presence of the aniline-like chromophore, a comparison with ECD spectra commonly associated with the PPI structure, due to the amide chromophore transitions, is not immediate. A distinctive ECD signature of PPI is a positive band around 210 nm in water or alcohol solvents, allied with the amide π–π* transition.^6,36^ Although our spectra do contain a positive ECD band in that region (Figure 5), transition and molecular orbital (MO) analysis after TD-DFT calculations (see below) highlights a major contribution of MOs localized on the aromatic rings in this region too, in addition to amide-centered transitions (Supporting Information).

**Figure 6:**
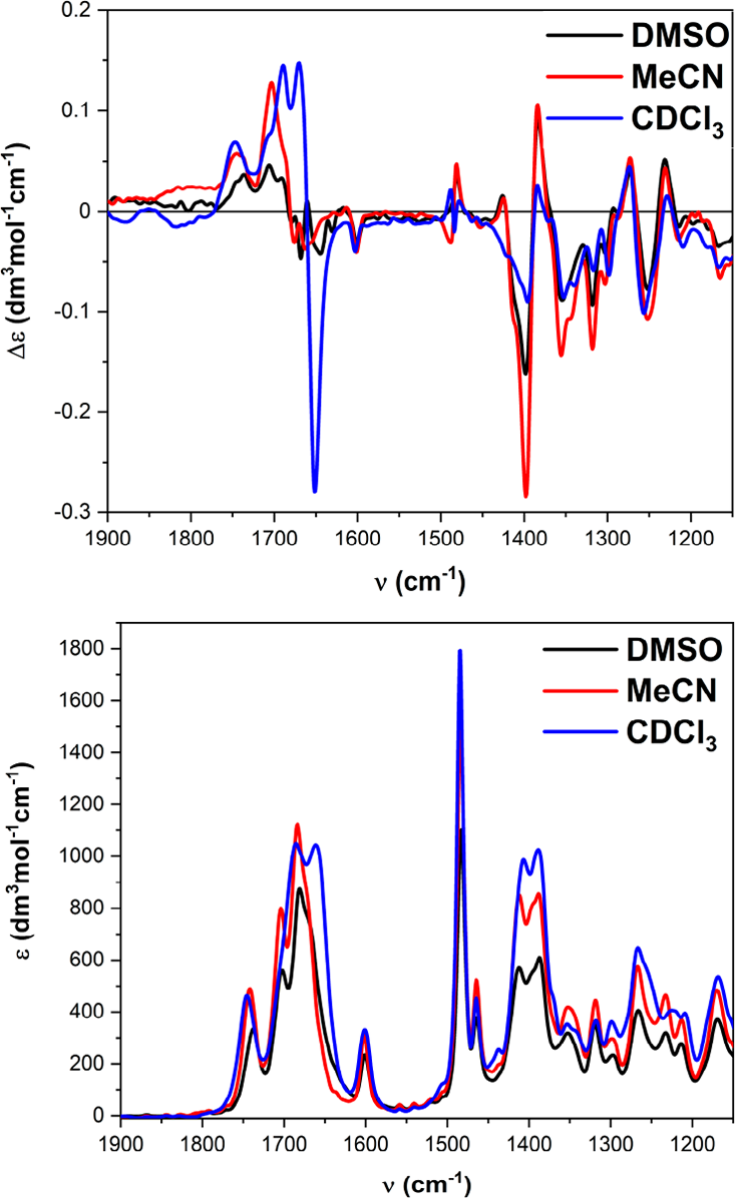
IR (bottom) and VCD (top) spectra of **4** measured in different solvents.

VCD spectra of compounds **2**–**4** parallel the behavior observed by ECD. The spectra become progressively stronger and better defined upon increasing the oligomer length *n*, and for the tetramer they acquire a consistent shape in the fingerprint region (<1450 cm^–1^) in all solvents (spectra for **4** in Figure 6; all other spectra shown in Figures S5 and S6). IR/VCD characterization of PPI helix has been sporadic, also because of its coexistence with PPII.^37,38^ According to Dukor and Keiderling, the VCD signature of PPI helix adopted by poly-l-proline consists of a negative couplet in the amide I region around 1650 cm^–1^ in D_2_O, similar to PPII but shifted to higher frequencies.^36^ Our spectra for the tetramer and the trimer (Figure S6) consistently display a negative VCD couplet between 1650 and 1700 cm^–1^, with solvent-dependent intensity, which by DFT calculations (vide infra) is assigned to amide I stretching vibrations. The IR spectra reported for PPI in D_2_O consist of three bands in the amide I region,^36^ also consistent with our results (Figure 6). We notice some discrepancy in this region between CDCl_3_ and the other two solvents, which we attribute to a different conformational manifold also suggested by NMR results. Thus, we may confirm the occurrence of the diagnostic IR and VCD signature established by Dukor and Keiderling on poly-l-proline also for other peptides when they assume a similar PPI helix conformation.

### Molecular Dynamics of Tetramer **4**

**Figure 7:**
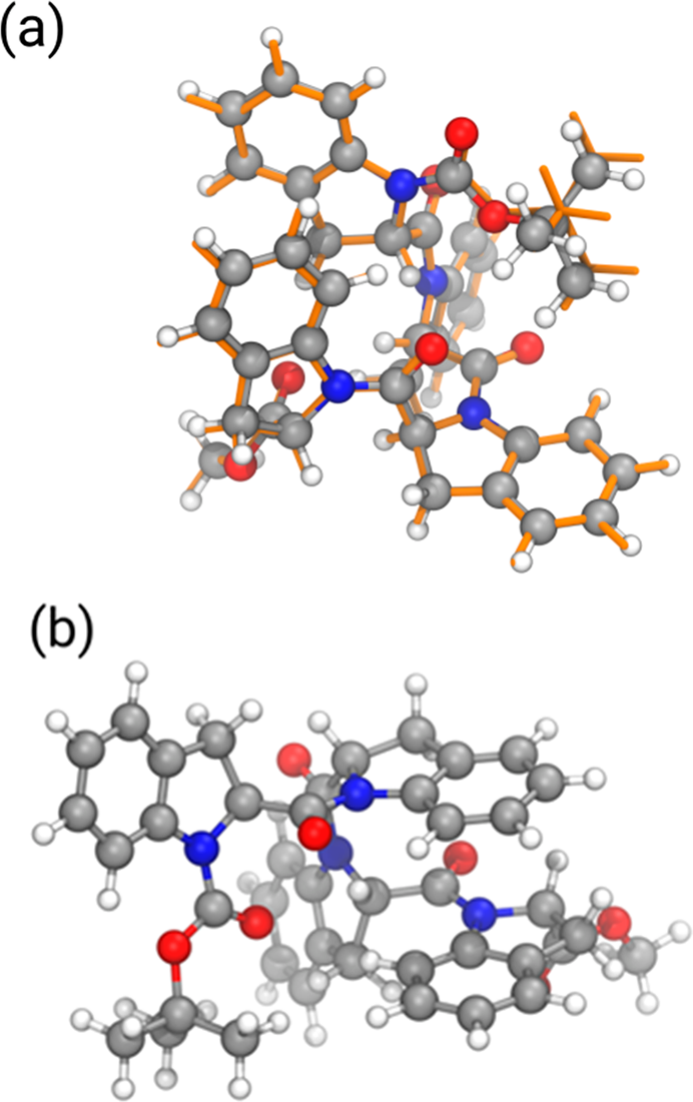
(a) Comparison between the DFT-optimized MD all-*cis* conformer (balls and sticks) and the DFT-optimized X-ray structure (orange sticks). (b) Structure of the most stable conformer according to DFT calculations.

To better characterize the possible conformations of tetramer **4**, we employed a molecular dynamics (MD) enhanced sampling strategy in explicit chloroform and DMSO solvents. Briefly, parallel-bias metadynamics (PBMETAD)^39,40^ was used to enhance the exploration of the tetramer free-energy landscape along with the four ω angles and the three amide ψ angles. A clustering procedure was employed to distinguish different conformers, and representative conformer structures were refined at the DFT level (see the Computational Details). Surprisingly, we found very small energy differences between the various conformers, many of them being within 1 kcal/mol from each other (see Table S2). As the intrinsic error associated with our calculations is likely larger than 1 kcal/mol, it is not possible to draw definitive conclusions from the calculations, but it is still possible to note some trends. First, of all of the conformers obtained from the MD simulations, the vast majority have at least two amide *cis* bonds, with only one having a single *cis* bond being selected among the stable ones per solvent. This suggests that conformations with more *cis* amide bonds were generally more stable. Interestingly, the all-*cis* conformer (see Figure 7a) is observed in both solvents. In DMSO, it is the second most stable conformer, with an energy difference of just 0.9 kcal/mol with respect to the absolute minimum (see Figure 7b). As was already mentioned, such an energy difference is smaller than the intrinsic error associated with the method, and therefore we can only conclude qualitatively that the all-*cis* conformer is thermodynamically favored in DMSO. This is not the case in chloroform, where such a conformer is predicted to be about 3 kcal/mol less stable than the minimum. The different behavior can be understood by looking at the large dipole moment of such a conformer, and in general of all of the predominantly *cis* conformers, which explains their higher stability in a polar solvent such as DMSO. Furthermore, the conformer with four *cis* amide bonds is very close to the X-ray structure (RMSD ≈ 0.6 A; see Figure 7a), which further supports the identification of such a conformer as the most thermodynamically stable one.

To obtain a more direct link with NMR experiments in DMSO, we analyzed the distances involved in interunit ROE interactions observed in NMR (Figures 3 and S1) along the PBMetaD. As the conformers interconvert slowly, we calculated ROE-like distances separately for each cluster (Table S4). While the all-*cis* conformer respects all of the experimental ROE constraints, all of the other conformers present at least two violations. This suggests that, of all of the conformations explored in our simulation, only one is compatible with all of the observed ROEs.

### Calculations of the VCD and ECD Spectra of **4**

X-ray crystallography, NMR evidence, and simulations all converge on a strongly preferred all-*cis* PPI-like structure for the tetramer. To reconcile these findings with chiroptical measurements, we run VCD calculations on two different structures, one obtained after relaxing the X-ray structure through DFT geometry optimizations at the B3LYP-D3/6-311+G(d,p) level and the other one being the all-*cis* structure obtained after the computational procedure at the same level of theory. As was stated above, the two structures are very similar to each other (Figure 7a). In particular, the two central units B and C are almost coincident, while the largest discrepancy occurs for the aromatic ring of unit D.

**Figure 8:**
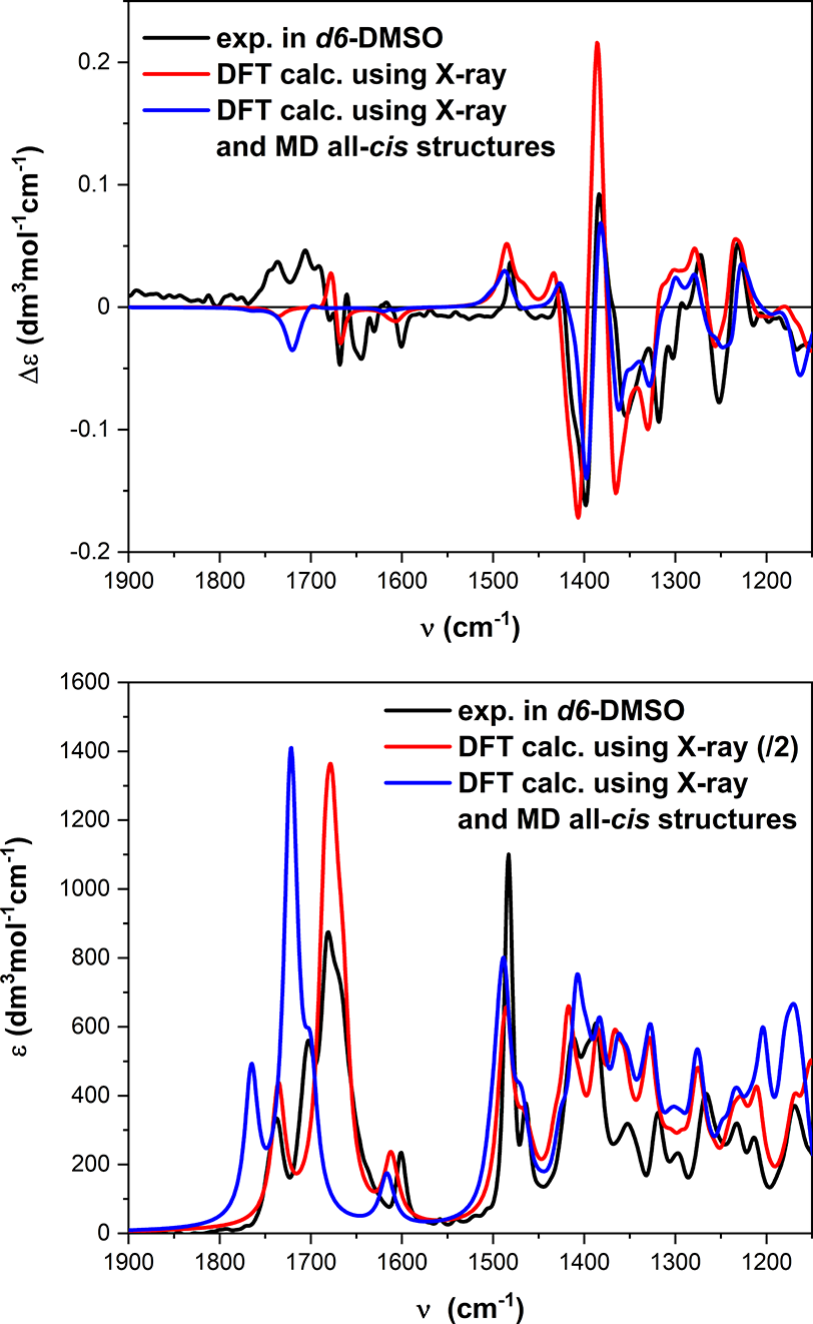
Comparison between the experimental IR (bottom) and VCD spectra (top) of **4** in DMSO-*d*_6_ and those calculated at the B3LYP/6-311+G(d,p)/PCM level using the two structures shown in Figure 7a. Plotting parameters: bandwidth, 9 cm^–1^; scaling factor, 0.983 for X-ray calc and 0.989 for MD calc.

In both cases, calculations were run both in vacuo and using the implicit PCM solvent model for DMSO. In fact, we aimed at reproducing VCD spectra recorded in this solvent where the major conformation found by NMR recalls the X-ray one. Figure 8 shows the comparison of VCD spectra calculated from the two aforementioned structures with PCM for DMSO with the experimental spectrum recorded in *d*_6_-DMSO. The agreement is remarkable in the fingerprint region, while it is poorer in the amide I region where, however, a negative couplet is correctly predicted. The normal modes responsible for the calculated couplet, shown in Figure S7, correspond to amide carbonyl stretching vibrations. Thus, VCD calculations further demonstrate that the X-ray geometry is largely retained in DMSO solution and confirm the VCD signature for the PPI helix established by Dukor and Keiderling, as a negative couplet in the amide I region.

**Figure 9:**
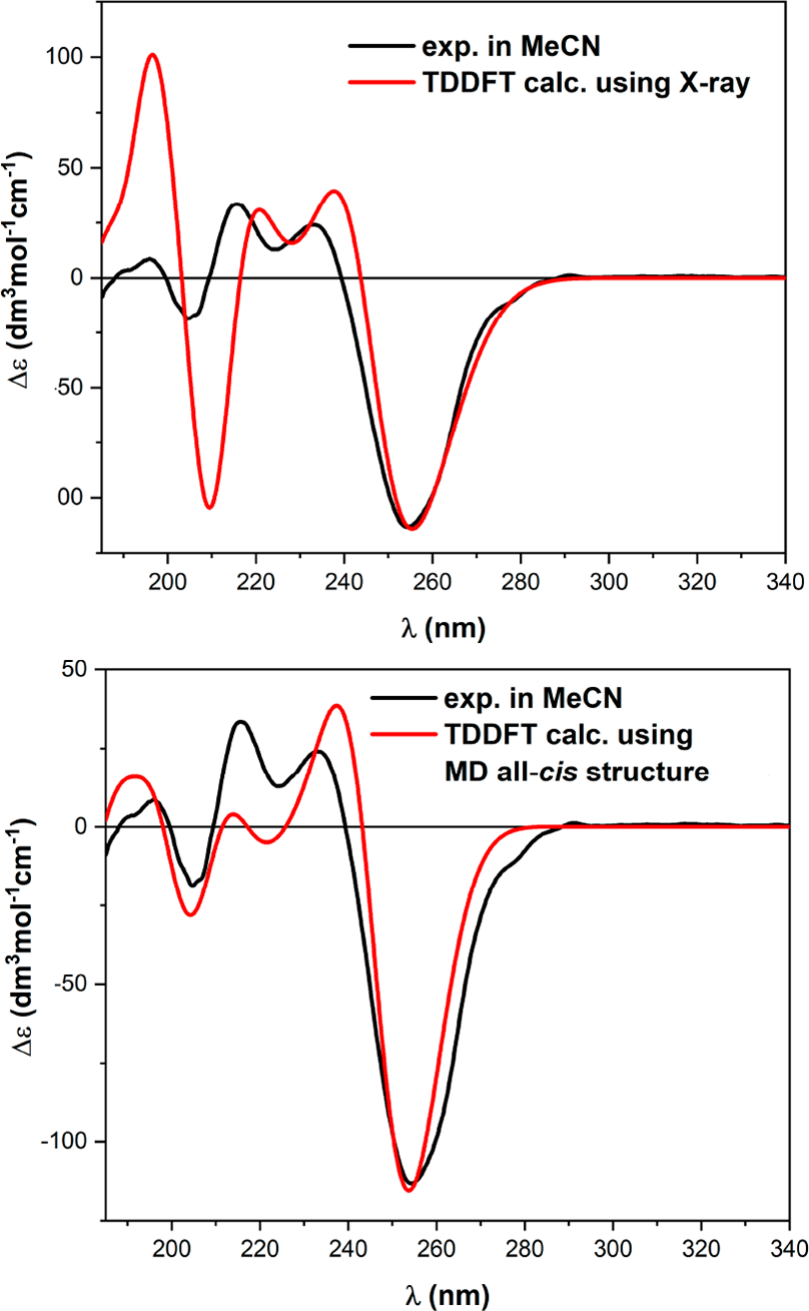
Comparison between the experimental ECD spectrum of **4** in CH_3_CN and those calculated at the TD-CAM-B3LYP/def2-SVP level using the structures obtained starting from the X-ray geometry (top) and MD simulations (bottom). Plotting parameters: bandwidth, 0.25 eV; wavelength shift, 20 nm; scaling, 1.20–1.35.

A consistent approach was adopted for ECD calculations of **4**. DFT-optimized geometries obtained starting from the X-ray and MD all-*cis* structures were employed in this case too. TD-DFT calculations were then run in vacuo or with the PCM solvent model for CH_3_CN or CH_3_OH, with B3LYP and CAM-B3LYP functionals, employing either the def2-SVP or the def2-TZVP basis sets. The results (Figures 9 and S8–S10) were all quite consistent and nicely reproduced the experimental ECD spectra. As an example, Figure 9 shows the comparison between the ECD spectrum of **4** in CH_3_CN and those calculated at the CAM-B3LYP/def2-SVP level using the two geometries (Figure 7a). The consistency between the experimental ECD spectra recorded in various solvents (Figure 5) and the calculation results demonstrates again that the all-*cis* PPI-type structure found in the solid state, and obtained from MD/DFT simulations, is preserved in different conditions and represents a strongly preferred fold for (2*S*)-Ind-based oligomers.

## Conclusion

We demonstrated the formation of a stable PPI helix structure, obtained from a short oligomer of (*S*)-indoline-2-carboxylic acid, in polar solvents. A thorough characterization, by NMR and X-ray diffraction, strongly supports the presence of a stable and basically unique structure in solution and in the solid phase. Measurements of the chiroptical properties, of the well-folded small oligomer, through ECD and VCD techniques, further confirmed the prevalence of a stable PPI fold and allowed the identification of diagnostic signals. The wealth of experimental evidence, combined with the conformational analysis and DFT simulation of the chiroptical properties, supports the assignment of a single, stable PPI conformation in polar solvents. In conclusion, the rare Polyproline I has been uniquely isolated, in solution and in the solid phase, allowing for a fine characterization, which will be useful for future identification of naturally occurring PPI or in the design of structured peptide sequences. These findings could aid in the design of β-turns or β-hairpins or in the synthesis of macrocyclic peptides.

## Experimental Section

### General Information and Materials

All nonaqueous reactions were run in oven-dried glassware under a positive pressure of argon, with exclusion of moisture from reagents and glassware, transferring solvents and liquid reagents with hypodermic syringes. The glassware has been dried with a heating gun under vacuum and allowed to cool under argon. Anhydrous solvents and liquid reagents were obtained using standard drying techniques. Solid reagents were of commercially available grade, used without further purification, and, when necessary, stored in a controlled atmosphere and/or at −20 °C. (*S*)-Indoline-2-carboxylic acid was purchased from abcr GmbH. Reactions were monitored by thin-layer chromatography using Merck silica gel 60 F254 plates. Visualization of the developed chromatogram was performed by UV absorbance, aqueous potassium permanganate, or iodine. Flash chromatography was performed using Sigma-Aldrich silica gel 60, particle size 40–63 μm, with the indicated solvent system.

Melting points were measured using a “Büchi Melting Point B-545” instrument.

NMR spectra of synthetic intermediates were recorded on a Bruker Avance DRx 400. Compounds **1**, **2**, and **3** were fully characterized with ^1^H NMR and ^13^C NMR on a Jeol instrument JNM-ECZ500R. Structural assignments were made with additional information from gCOSY, gHSQC, and gHMBC experiments. For compound **4**, ^1^H NMR, ^13^C NMR, gCOSY, gHSQC, ROESY, TOCSY, and temperature titration were recorded on a VARIAN INOVA 600 MHz instrument. Chemical shifts are reported in ppm with the deuterated solvent signal as the internal standard. Data are reported as follows: chemical shift, integration, multiplicity (s = singlet, d = doublet, t = triplet, q = quartet, qn = quintet, m = multiplet, and br = broad), with the coupling constant in hertz.

ECD spectra were measured with a Jasco J-715 spectropolarimeter with the following conditions: scan speed 100 nm/min; response 0.5 s; data pitch 0.2 nm; bandwidth 1.0 nm; 4 accumulations. VCD and IR spectra were recorded using a Jasco FVS-6000 VCD spectrometer with the following conditions: resolution 4 cm^–1^; range 2000–900 cm^–1^; 4000 accumulations.

The crystal structure measurements have been done on a Bruker SMART BREEZE CCD diffractometer equipped with a graphite monochromate Mo Kα radiation.

HPLC-ESI-Q/ToF flow injection analyses (FIA) were carried out with a 1200 Infinity HPLC (Agilent Technologies, U.S.), coupled with a quadrupole-time-of-flight tandem mass spectrometer (6530 Infinity Q-TOF; Agilent Technologies) through a Jet Stream ESI interface (Agilent). Mass Hunter Workstation Software (B.04.00) was used to control the HPLC and the mass spectrometer, for data acquisition and for data analysis.

#### Boc-(2*S*)-Ind-OMe (**1**)

(*S*)-Indoline-2-carboxylic acid (*H*-(2*S*)Ind-OH) (2.8 g, 17.2 mmol) was dissolved in 280 mL of methanol and cooled to 0 °C with an ice bath. To this suspension was added dropwise thionyl chloride (1.87 mL, 25.7 mmol, 1.5 equiv). The reaction mixture was stirred for 1 h and allowed to reach room temperature. Afterward, the resulting solution was heated at 70 °C, in an oil bath, and allowed to stir at reflux for 16 h. After being cooled at room temperature, the reaction mixture was concentrated under reduced pressure. The residue was dissolved in ethyl acetate (EtOAc) and washed with an aqueous saturated solution of sodium bicarbonate (NaHCO_3_) three times. The combined organic layers were washed with brine, dried over Na_2_SO_4_, and concentrated under reduced pressure. The resulting crude material was purified by flash chromatography on silica gel using 10–50% EtOAc in hexane to give the desired product (H-(2*S*)-Ind-OMe) as a white solid in 82% yield (2.5 g, 14.1 mmol). ^1^H NMR (400 MHz, CDCl_3_): δ = 7.12–6.99 (m, 2H), 6.79–6.68 (m, 2H), 4.39 (dd, *J* = 10.2, 5.5 Hz, 1H), 3.76 (s, 3H), 3.36 (m, 2H). 2.20 g (12.4 mmol) of the obtained *H*-(2*S*)-Ind-OMe was dissolved in 30 mL of dioxane and 12 mL of water. 4.42 g (20.3 mmol) of (Boc)_2_O was dissolved in 10 mL of dioxane and dropped into the reaction mixture, which was left to stir for 16 h at rt. 250 mL of EtOAc was added to the solution, and the resulting mixture was washed with HCl 1 M for 3 × 250 mL. The combined organic layers were dried over Na_2_SO_4_, filtered, and concentrated under reduced pressure. The resulting solid was purified by flash chromatography on silica gel using 10–50% EtOAc in hexane. The desired product was obtained as a white solid in an isolated yield of 75% (9.3 mmol, 2.58 g). TLC *R_f_* = 0.3 (Hex:EtOAc = 8:2), mp = 54–56 °C.

^1^H NMR (500 MHz, CDCl_3_): δ 7.88 + 7.48 (1H, d, *J* = 7.0 Hz + s), 7.25–6.92 (3H (7.19, m + 7.10, d, *J* = 7.4 Hz + 6.95, t, *J* = 7.4 Hz)), 5.00–4.75 (1H (4.92, bs + 4.85, bs)), 3.74 (3H, s), 3.48 (1H, m), 3.10 (1H, dd, *J* = 16.3, 3.8 Hz), 1.68–1.40 (9H (1.59, bs + 1.48, bs)). ^1^H NMR (500 MHz, DMSO-*d*_6_): δ 7.72–6.85 (4H (7.70, d, *J* = 7.7 Hz + 7.37, bs + 7.13, m + 6.91, td, *J* = 7.5, 0.9 Hz)), 4.85 (1H, dd, *J* = 11.6, 4.4 Hz), 3.65 (3H, s), 3.50 (1H, dd, *J* = 16.0, 11.6 Hz), 3.01 (1H, d, *J* = 16.0 Hz), 1.55–1.30 (9H (1.50, bs + 1.38 bs)). ^13^C{^1^H} NMR (150 MHz, DMSO-*d*_6_): δ 172.1, 151.0, 142.2, 128.5, 127.5, 124.7, 122.4, 80.6, 59.8, 51.8, 31.8, 27.7. HRMS (ESI-TOF) *m*/*z*: [M + Na]^+^ calcd for C_15_H_19_NO_4_Na, 300.1213; found, 300.1206. Analytical HPLC purity >99%.

#### Boc-((2*S*)-Ind)_2_-OMe (**2**)

(*S*)-Indoline-2-carboxylic acid (*H*-(2*S*)-Ind-OH) (1.2 g, 7.2 mmol) was dissolved in 20 mL of a dioxane:water (1:1) mixture, and 1.7 g (7.8 mmol) of (Boc)_2_O was added. The reaction mixture was left to stir at rt for 16 h. The resulting solution was then concentrated under reduced pressure, and the residue acidified with HCl 1 M was washed three times with EtOAc. The combined organic layers were dried over Na_2_SO_4_, filtered, and concentrated under reduced pressure. The crude product Boc-(2*S*)-Ind-OH was obtained as a white solid with 87% yield (6.3 mmol, 1.66 g) and used without further purification. When needed, the reaction was performed several times on the same scale.

^1^H NMR (400 MHz, CDCl_3_): δ = 7.89 (s, 1H), 7.46 (s, 1H), 7.24–7.09 (m, 1H), 6.96 (t, *J* = 7.4 Hz, 1H), 4.89 (s, 1H), 3.53 (s, 1H), 3.21 (s, 1H), 1.55 (d, *J* = 36.2 Hz, 9H).

To a 45 mL dry dichloromethane stirred solution of Boc-(2*S*)-Ind-OH (1.75 g, 6.63 mmol) and Mukaiyama reagent (2.37 g, 9.3 mmol) was added 1.17 g (6.63 mmol) of *H*-(2*S*)-Ind-OMe. 2.6 mL of TEA (18.6 mmol) was dropped into the solution, and the reaction mixture was heated at reflux, in an oil bath, for 16 h before being allowed to cool at room temperature. The reaction mixture was then diluted with DCM and washed with HCl 1 M, a saturated solution of Na_2_CO_3_, and brine. The combined organic layers were dried over Na_2_SO_4_, filtered, and concentrated under reduced pressure. The crude product was purified by flash chromatography on silica gel using 10–50% EtOAc in hexane, obtaining the desired product as a white solid with 70% yield (4.64 mmol, 1.96 g). TLC *R_f_* = 0.43 (Hex:EtOAc = 7.5:2.5), mp = 87–89 °C.

^1^H NMR (500 MHz, CDCl_3_): δ 8.30–6.85 (8H (8.19, bs + 7.98, bs + 7.91, d, *J* = 8.0 Hz + 7.52, d, *J* = 8.1 Hz + 7.31–7.00, m + 6.95–6.88, m)), 5.60–4.85 (2H (5.56, dd, *J* = 11.2, 3.2 Hz + 5.48, dd, *J* = 11.2, 4.0 Hz + 5.27, dt, *J* = 11.0, 3.7 Hz + 4.98, bs)), 3.85–3.0 (7H (3.81–3.61, m + 3.66, s + 3.64, s + 3.51, td, *J* = 16.6, 11.4 Hz + 3.36–3.22, m + 3.11 (ddd, *J* = 29.5, 16.7, 2.9 Hz))), 1.62–1.45 (9H (1.58, bs + 1.50, bs + 1.34, bs)). ^1^H NMR (500 MHz, DMSO-*d*_6_): δ 8.0–6.85 (8H (7.90, d, *J* = 7.9 Hz + 7.77, d, *J* = 7.8 Hz + 7.71, d, *J* = 8.0 Hz + 7.44–6.83, m)), 5.58–4.85 (2H (5.52, dd, *J* = 11.5, 3.6 Hz + 5.20, bs + 5.10, m + 4.98, bs + 4.90, m)), 3.92–2.89 (7H (3.86, m + 3.71, m + 3.68, s + 3.53, m + 3.25, m + 3.08–2.91, m)), 1.55–1.22 (9H (1.51, bs + 1.49, bs + 1.40, bs + 1.25, bs)). ^13^C{^1^H} NMR (150 MHz, DMSO-*d*_6_): δ 171.6, 169.9, 152.0, 151.3, 143.4, 143.1, 140.2, 131.5, 130.4, 128.8, 127.8, 127.8, 127.7, 127.6, 126.5, 126.4, 125.1, 125.0, 124.4, 124.4, 124.1, 124.1, 122.6, 122.6, 122.6, 122.6, 122.5, 122.5, 117.0, 114.3, 114.3, 114.2, 113.9, 113.8, 113.8, 113.8, 110.0, 80.7, 60.2, 59.9, 28.4, 28.4, 28.1, 28.0, 28.0.

HRMS (ESI-TOF) *m*/*z*: [M + Na]^+^ calcd for C_24_H_26_N_2_O_5_Na, 445.1734; found, 445.1733. Analytical HPLC purity 98%.

#### Boc-((2*S*)-Ind)_3_-OMe (**3**)

Compound **2** (1.8 g, 4.2 mmol) was dissolved in 35 mL of a THF:H_2_O:MeOH = 4:1:1 mixture, and 527 mg (12.6 mmol) of LiOH·H_2_O was added. The reaction mixture was left to stir for 3 h at room temperature. The solution was diluted with EtOAc and acidified with HCl 1 M. The obtained mixture was extracted three times with EtOAc, and the combined organic layers were dried over Na_2_SO_4_, filtered, and concentrated under reduced pressure. The crude product Boc-((2*S*)-Ind)_2_-OH obtained in quantitative yield as a white solid was used without further purification.

^1^H NMR (400 MHz, CDCl_3_): δ = 8.28–7.85 (m, 1H), 7.57–6.84 (m, 7H), 5.61–5.12 (m, 2H), 3.81–3.19 (m, 4H), 1.64–1.31 (m, 9H).

To a 25 mL dry DCM stirred solution of Boc-((2*S*)-Ind)_2_-OH (997 mg, 2.4 mmol) and Mukaiyama reagent (845 mg, 3.3 mmol) was added 434 mg (2.45 mmol) of *H*-(2*S*)-Ind-OMe. 920 μL of TEA (6.6 mmol) was dropped into the solution, and the reaction mixture was heated at reflux, in an oil bath, for 16 h before being allowed to cool at room temperature. The reaction mixture was then diluted with DCM and washed with HCl 1 M, a saturated solution of Na_2_CO_3_, and brine. The combined organic layers were dried over Na_2_SO_4_, filtered, and concentrated under reduced pressure. The crude product was purified by flash chromatography on silica gel using 10–30% EtOAc in toluene, to obtain the desired product as a white solid with 60% yield (1.44 mmol, 818 mg). The reaction was performed two times on the same scale. TLC *R_f_* = 0.81 (toluene:EtOAc = 7:3), mp = 122–124 °C.

^1^H NMR (500 MHz, CDCl_3_): δ 8.50–6.77 (12H, m), 5.95–4.56 (3H, m), 3.92–2.92 (9H, m), 1.70–1.31 (9H, m). ^1^H NMR (500 MHz, DMSO-*d*_6_): δ 8.35–6.75 (12H (8.35–7.98, m + 7.92–7.75, m + 7.50–7.40, m + 7.31–6.78, m)), 5.90–4.80 (3H (5.90–5.69, m + 5.65–5.44, m + 5.42–5.22, m + 5.20–5.10, m + 5.08–4.77, m)), 3.95–2.90 (9H (3.95–3.50, m + 3.50–2.90, m)), 1.66–1.30 (9H, m). ^13^C{^1^H} NMR (150 MHz, DMSO-*d*_6_): δ 173.1, 172.6, 171.5, 170.9, 170.0, 169.4, 169.3, 169.1, 152.3, 144.4, 143.1, 142.8, 130.4, 130.0, 129.8, 128.7, 128.1, 127.9, 127.8, 127.8, 126.6, 125.4, 125.3, 125.2, 124.9, 124.8, 124.6, 124.2, 124.1, 122.7, 116.8, 116.7, 116.6, 114.6, 114.0, 81.3, 81.2, 61.3, 61.1, 61.0, 60.4, 60.3, 60.2, 53.9, 53.3, 52.8, 34.8, 33.3, 33.2, 31.5, 28.7, 28.5, 28.3, 28.1, 24.0, 22.6, 21.3, 14.6, 14.5. HRMS (ESI-TOF) *m*/*z*: [M + Na]^+^ calcd for C_33_H_33_N_3_O_6_Na, 590.2261; found, 590.2262. Analytical HPLC purity 99%.

#### Boc-((2*S*)-Ind)_4_-OMe (**4**)

Compund **3** (1 g, 1.76 mmol) was dissolved in 20 mL of a THF:H_2_O:MeOH = 4:1:1 mixture, and 222 mg (5.3 mmol) of LiOH·H_2_O was added. The reaction mixture was left to stir for 3 h at room temperature. The solution was diluted with EtOAc and acidified with HCl 1 M. The obtained mixture was extracted three times with EtOAc, and the combined organic layers were dried over Na_2_SO_4_, filtered, and concentrated under reduced pressure. The crude product Boc-((2*S*)-Ind)_3_-OH obtained in quantitative yield as a white solid was used without further purification.

^1^H NMR (400 MHz, CDCl_3_): δ = 8.42–8.10 and 7.96–7.86 (m, 0.5H), 7.57–6.77 (m, 11.5H), 5.95–5.02 (m, 3H), 4.04–2.97 (m, 6H), 1.70–1.36 (m, 9H).

To a 10 mL dry DCM stirred solution of Boc-((2*S*)-Ind)_3_-OH (900 mg, 1.6 mmol) and Mukaiyama reagent (580 mg, 2.27 mmol) was added 290 mg (1.63 mmol) of *H*-(2*S*)-Ind-OMe. 630 μL of TEA (4.5 mmol) was dropped into the solution, and the reaction mixture was heated at reflux, in an oil bath, for 16 h before being allowed to cool at room temperature. The reaction mixture was then diluted with DCM and washed with HCl 1 M, a saturated solution of Na_2_CO_3_, and brine. The combined organic layers were dried over Na_2_SO_4_, filtered, and concentrated under reduced pressure. The crude product was purified by flash chromatography on silica gel using 10–40% EtOAc in toluene, to obtain the desired product as a white solid with 32% yield (0.5 mmol, 365 mg). TLC *R_f_* = 0.31 (toluene:EtOAc = 8:2), mp = 222–224 °C (dec.)

^1^H NMR (600 MHz, CDCl_3_): δ 8.40–6.40 (16H, m), 5.92–4.80 (4H, m), 4.0–2.7 (11H, m), 1.70–1.20 (9H (1.63, s + 1.57, s + 1.38, s + 1.25, s)). ^1^H NMR (600 MHz, DMSO-*d*_6_): δ 8.20–6.65 (16H (8.06, dd, *J* = 14.5, 8.3 Hz + 7.81, d, *J* = 8.0 Hz + 7.31–6.70, m)), 5.40 (1H, dd, *J* = 10.9, 2.0 Hz), 5.34 (1H, dd, *J* = 10.9, 2.0 Hz), 5.17 (1H, dd, *J* = 10.5, 1.6 Hz), 5.04 (1H, dd, *J* = 11.1, 3.8 Hz), 4.02–3.24 (11H, m), 1.52 (9H, s). ^13^C{^1^H} NMR (150 MHz, DMSO-*d*_6_): δ 173.2, 170.7, 169.8, 168.7, 152.4, 144.2, 143.8, 142.4, 130.1, 129.5, 129.3, 129.3, 128.7, 127.9, 127.9, 127.8, 127.7, 125.4, 125.2, 125.0, 124.9, 124.7 124.5, 124.2, 122.5, 116.6, 116.5, 116.4, 114.4, 81.3, 60.8, 60.6, 60.4, 60.1, 54.3, 34.7, 34.5, 33.2, 33.1, 28.7. HRMS (ESI-TOF) *m*/*z*: [M + Na]^+^ calcd for C_42_H_40_N_4_O_7_Na, 735.2789; found, 735.2773. Analytical HPLC purity >99%. A complete assignment of NMR signals for **4** is given below.

### Crystal Structure Determination

The structure solution was obtained by automated direct methods contained in the SHELX suite.^41^ The absolute configuration of the molecule was inferred starting from the known configuration of the stereogenic carbons of proline. After the hydrogen atoms were placed in calculated positions, the non-hydrogen atoms were refined with anisotropic thermal parameters. At the end of refinement, the thermal ellipsoids of some atoms of the *t*-butyl carbamate protecting group were excessively elongated, suggesting the presence of some conformational disorder, which, however, could not be modeled.

### NMR Characterization

^1^H NMR characterization of the PPI helix for compound **4** in DMSO-*d*_6_ was performed on a 600 MHz instrument.

On the basis of the ^1^H–^13^C scalar correlations detected in the HSQC map (Supporting Information), resonances between 4.9 and 5.5 ppm and between 2.8 and 4.1 ppm were attributed to the methine (H_α_) and diastereotopic methylene protons of the pentatomic rings, respectively. Aromatic protons originated a complex pattern of signals in the high-frequency spectral region (6.6–8.2 ppm), and sharp singlets at 1.52 and 3.86 ppm were unambiguously assigned to the *t*-butyl and methoxy groups, respectively. In the attribution of NMR signals of Boc-((2*S*)-Ind)_4_-OMe, the four monomeric units were named sequentially as A–D (Figure 3), with end A bearing the *t*-butyl moiety. Through-space dipolar interactions detected in the 2D ROESY map (Supporting Information) and scalar correlations detected in the 2D COSY and 2D TOCSY allowed one to distinguish the resonances bearing to every unit (Table 3). Among methine protons CH_α_, only the one centered at 5.05 ppm originated a dipolar interaction at the frequency of the *t*-butyl group and was then attributed to CH_α_^A^. Intraunit ROEs produced by CH_α_^A^ allowed one to distinguish H_β_^A^ (3.45 ppm) and H_β′_^A^ (2.92 ppm), cisoid and transoid to it, respectively. Importantly, CH_α_^A^ gave a dipole–dipole interaction with the methine proton resonating at 5.18 ppm, which was then attributed to CH_α_^B^ of the adjacent unit B. The above said dipolar interaction also led one to assess that the amide junction between the two adjacent A and B units of the prevailing species showed a strong preference for the *cis* conformation, as was already demonstrated for the dimer Ac-((2*S*)-Ind)_2_-OMe in the same solvent. Dipolar interactions generated by H_α_^B^ at the frequencies of 3.67, 3.32, and 5.35 ppm led to the assignment of adjacent cissoid H_β_^B^ (3.67 ppm) and transoid H_β′_^B^ (3.32 ppm) and to the identification of the methine at the chiral center of the third unit, H_α_^C^. Likewise, ROE effects produced by this last proton allowed one to attribute the methylene protons of unit C (H_β_^C^ at 3.98 ppm and H_β′_^C^ and 3.47 ppm, cisoid and transoid to H_α_^C^, respectively) and the methine proton H_α_^D^ (5.40 ppm) of the last unit. Intraunit ROEs given by this last proton led to the assignment of its cissoid H_β_^D^ (3.84 ppm) and transoid H_β′_^D^ (3.48 ppm). Regarding the aromatic proton resonances, peri protons H_ar4_ of every unit were assigned on the basis of the ROE effects produced by their adjacent methylene protons, and the TOCSY and COSY correlations detected starting from peri protons led to the complete assignment of aromatic protons (Supporting Information).

**Table 3:** ^1^H (600 MHz, DMSO-*d*_6_, 10 mM, 25 °C) Chemical Shifts (δ, ppm) of Boc-((2*S*)-Ind)_4_-OMe (**4**)

proton	A unit	B unit	C unit	D unit
H_α_	5.05	5.18	5.35	5.40
H_β_	3.45	3.67	3.98	3.84
H_β′_	2.92	3.32	3.47	3.48
H_ar4_	6.74	7.20	7.19	7.30
H_ar3_	6.80	7.02	7.02	7.03
H_ar2_	7.13	7.20	7.19	6.96
H_ar1_	7.81	8.05	8.07	7.81
*t*-Bu	1.52			
OMe				3.86

### Computational Details

#### Molecular Dynamics Simulations Run with the Amber Software

The tetramer was described by adapting the protein ff14SB parameters for the amide backbone,^42^ while specific parameters for the aromatic side chain were taken from the General Amber Force Field (GAFF).^43^ Torsional parameters for rotation around the amide bond were refit from calculations at the B3LYP/6-311G(d,p) level and substituted to the force field parameters. The tetramer was placed in a DMSO or chloroform solvent box, and the system was equilibrated (the equilibration protocol is described in detail in the Supporting Information). PBMETAD simulations were run for 1.5 μs in both solvents, applying a parallel metadynamics bias on each ω angle and the three ψ angles between the indoline residues. We used PBMETAD as it allows biasing several collective variables (CVs) at once in a parallel fashion. As the barriers separating the conformers are almost exclusively along these torsions, this strategy allows an exploration as much as possible unbiased of the tetramer’s conformational space. PBMETAD simulations were run with Amber interfaced with the PLUMED enhanced sampling library.^44^ The sampled structures were reweighted using the approach by Tiwary and Parrinello and clustered using the K-means algorithm based on the ω and ψ angles.^45^ One structure from each cluster, representative of one conformation, was extracted for the QM analysis.

#### QM Calculations

All QM calculations were run with *Gaussian 16* software.^46^ The structures extracted from DMSO and chloroform simulations were optimized in PCM solvent^47^ at the B3LYP-D3(BJ)/6-311+G(d,p) level of theory. Frequency calculations were used to ensure the achievement of a minimum, to compute the free energy differences, and to calculate the VCD and IR spectra. Excited-state calculations were run with the TD-DFT method employing the B3LYP and CAM-B3LYP functionals, the def2-SVP or def2-TZVP basis sets, either in vacuo or in PCM solvent. The number of roots was 100 for CAM-B3LYP and 150 for B3LYP. IR/VCD and UV/ECD spectra were generated using SpecDis v. 1.71;^48^ plotting parameters are reported in the figure legends.

#### Equilibration Protocol

The tetramer was placed in a truncated octahedron box with 1080 DMSO molecules (or 952 CHCl_3_ molecules) and minimized with 2000 conjugate gradient steps. Each box was heated to the desired 300 K temperature in a 100 ps simulation run. Additional 100 ps NPT simulations were performed to equilibrate the density of the solvent. Finally, a production run of 40 ns in the NPT ensemble was run without any bias. In all simulations, we used a 2.0 fs integration time step combined with the SHAKE algorithm. The Langevin thermostat was used to control the temperature, whereas the pressure was controlled by the Monte Carlo barostat implemented in Amber. In all unbiased runs, the tetramer remained in its initial conformation as was monitored by the ω and ψ angles.

#### Details on the Parallel-Bias Metadynamics

To explore the conformations of the tetramer in both solvents, we employed well-tempered Parallel-bias Metadynamics (PBMETAD) simulations. In PBMETAD, multiple one-dimensional bias potentials are applied on several collective variables (CV) of the system. As in standard Metadynamics, the bias potential is expressed as a sum of Gaussian functions. However, here the bias on one CV depends on how much the other CVs have been biased, ensuring the convergence of the monodimensional bias to the correct limit. Here, we considered each peptide ω and ψ angle as a CV, for a total of seven CVs in the PBMETAD simulations. We found it necessary to bias also the ψ angles, as the indoline side chains give rise to significant barriers also along ψ rotation. Well-tempered metadynamics was applied, with a bias factor of 20. This value was estimated from the potential energy barrier along ω rotation (∼16 kcal/mol).

## Supporting Information Available

Full characterization of the synthesized compounds, NMR experiments, calculation details, HPLC–MS spectra, and crystallographic data for compound **4** (PDF)
FAIR data, including the primary NMR FID files, for compounds **1**, **2**, **3**, and **4** (ZIP)

## Abbreviations

PPI: polyproline I
PPII: polyproline II
*H*-(2*S*)-Ind-OH: (*S*)-indoline-2-carboxylic acid
HATU: (1-[bis(dimethylamino)methylene]-1*H*-1,2,3-triazolo[4,5-*b*]pyridinium 3-oxide hexafluorophosphate
HBTU: (2-(1*H*-benzotriazol-1-yl)-1,1,3,3-tetramethyluronium hexafluorophosphate
FDPP: pentafluorophenyl diphenylphosphinate
DCC: *N*,*N*′-dicyclohexylcarbodiimide
EDCI: 1-ethyl-3-(3-dimethylaminopropyl)carbodiimide
TFA: trifluoroacetic acid
TLC: thin layer chromatography
DCM: dichloromethane
CDCl_3_: deuterated chloroform
DMSO-*d*_6_: deuterated dimethylsulfoxide
