# Supplementary material for: A Unique and Stable Polyproline I Helix Sorted out from Conformational Equilibrium by Solvent Polarity
Source: J Org Chem. 2022 Oct 15;87(21):13715–25. doi: 10.1021/acs.joc.2c01377 (PMC9639007; doi:10.1021/acs.joc.2c01377)
Supplement: Supplementary file 1 — jo2c01377_si_001.pdf [file jo2c01377_si_001.pdf]

SUPPORTING INFORMATION

A unique and stable Polyproline I helix sorted out from conformational equilibrium by solvent polarity

Matteo Pollastrini, Luca Pasquinelli, Marcin Górecki, Federica Balzano, Lorenzo Cupellini, Filippo Lipparini, Gloria Uccello Barretta, Fabio Marchetti, Gennaro Pescitelli*, Gaetano Angelici*

Dipartimento di Chimica e Chimica Industriale, Università di Pisa, Via G. Moruzzi 13, 56124 Pisa, Italy

Institute of Organic Chemistry, Polish Academy of Sciences, ul. Kasprzaka 44/52, 01-224 Warsaw, Poland

* Email: gennaro.pescitelli@unipi.it

* Email: gaetano.angelici@unipi.it

KEYWORDS “Polyproline I, foldamers, proline mimetic, conformational studies”

Contents

^{1}H NMR spectrum of Boc-(2*S*)-Ind-OMe (**1**) in $CDCl_3$ (0.1 M) at 500 MHz SI3

^{1}H NMR spectrum of Boc-(2*S*)-Ind-OMe (**1**) in DMSO-d_6 (0.1 M) at 500 MHz SI4

^{13}C{^{1}H}NMR spectrum of Boc-(2*S*)-Ind-OMe (**1**) in DMSO-d_6 at 125 MHz SI5

^{1}H NMR spectrum of Boc-((2*S*)-Ind)$_2$-OMe (**2**) in $CDCl_3$ (0.1 M) at 500 MHz SI6

^{1}H NMR spectrum of Boc-((2*S*)-Ind)$_2$-OMe (**2**) in DMSO-d_6 (0.1 M) at 500 MHz SI7

^{13}C{^{1}H}NMR spectrum of Boc-((2*S*)-Ind)$_2$-OMe (**2**) in DMSO-d_6 at 150 MHz SI8

^{1}H NMR spectrum of Boc-((2*S*)-Ind)$_3$-OMe (**3**) in $CDCl_3$ (0.1 M) at 500 MHz SI9

^{1}H NMR spectrum of Boc-((2*S*)-Ind)$_3$-OMe (**3**) in DMSO-d_6 (0.1 M) at 500 MHz SI10

^{13}C{^{1}H}NMR spectrum of Boc-((2*S*)-Ind)$_3$-OMe (**3**) in DMSO-d_6 at 125 MHz SI11

^{1}H NMR spectrum of Boc-((2*S*)-Ind)$_4$-OMe (**4**) in $CDCl_3$ (0.1 M) at 500 MHz SI12

^{1}H NMR spectrum of Boc-((2*S*)-Ind)$_4$-OMe (**4**) in DMSO-d_6 (0.1 M) at 500 MHz SI13

^{13}C{^{1}H}NMR spectrum of Boc-((2*S*)-Ind)$_4$-OMe (**4**) in DMSO-d_6 at 150 MHz SI14

2D HSQC (600 MHz, DMSO-d6, 10 mM, 25 °C) map of Boc-((2*S*)-Ind)$_4$-OMe (**4**) SI15

2D ROESY (600 MHz, DMSO-d6, 10 mM, 25 °C, mix 0.6 s) map of Boc-((2*S*)-Ind)$_4$-OMe (**4**) SI16

2D COSY (600 MHz, DMSO-d6, 10 mM, 25 °C) map of Boc-((2*S*)-Ind)$_4$-OMe (**4**) SI17

2D TOCSY (600 MHz, DMSO-d6, 10 mM, 25 °C) map of Boc-((2*S*)-Ind)$_4$-OMe (**4**) SI18

HPLC-MS analysis of (**1**) SI19

HPLC-MS analysis of (**2**) SI20

HPLC-MS analysis of (**3**) SI21

HPLC-MS analysis of (**4**) SI22

Table S1. Crystal data and structure refinement of (**4**) SI23

Table S2. Description of main C–H···O interactions SI24

Table S3. Calculated energy differences between conformers SI25

Torsional parameters. SI26

Table S4. Calculated ROE distances along the PBMetaD in DMSO SI27

Figure S1. Intra-unit Ha-Hb(cis) and inter-unit Ha-Hb'(trans) ROE interactions of 4 shown on the DFT-optimized X-ray structure. SI28

Figure S2. Experimental absorption spectra measured for compounds 1-4 in different solvents.... SI29

Figure S3. Variable-temperature ECD spectra of **4** measured in MeCN. SI30

Figure S4. Absorption and ECD spectra of **4** measured in TFE. SI31

Figure S5. IR (bottom) and VCD (top) spectra of **2** measured in different solvents. SI32

Figure S6. IR (bottom) and VCD (top) spectra of **3** measured in different solvents. SI33

Figure S7. Expansion of the VCD spectrum of (**4**) calculated at B3LYP/6-311+G(d,p)/PCM level using the DFT-optimized X-ray structure. SI34

Figure S8. Calculated ECD spectra for **4** at TD-CAM-B3LYP/def2-SVP and at TD-CAM-B3LYP/def2-TZVP level using the structure obtained starting from the X-ray geometry. SI35

Figure S9. Calculated ECD spectra for 4 at TD-CAM-B3LYP/def2-SVP/PCM for MeOH level using the structures obtained starting from the X-ray geometry and MD simulations. SI36

Figure S10. Calculated ECD spectra for **4** at TD-B3LYP/def2-SVP level in vacuum and with PCM solvent model for MeOH using the structures obtained starting from the X-ray geometry and MD simulations. SI37

Figure S11. Calculated ECD spectrum (TD-CAM-B3LYP/def2-SVP/PCM for MeCN) using the MD-derived structure with band assignment SI38

Figure S12. View of the molecular structure of (**4**). Ellipsoids are represented at 20% probability. SI39

Coordinates of the DFT-optimized geometries SI40

Structures and energies of the optimized clusters SI44

^{1}H NMR spectrum of Boc-(2*S*)-Ind-OMe (**1**) in $CDCl_3$ (0.1 M) at 500 MHz

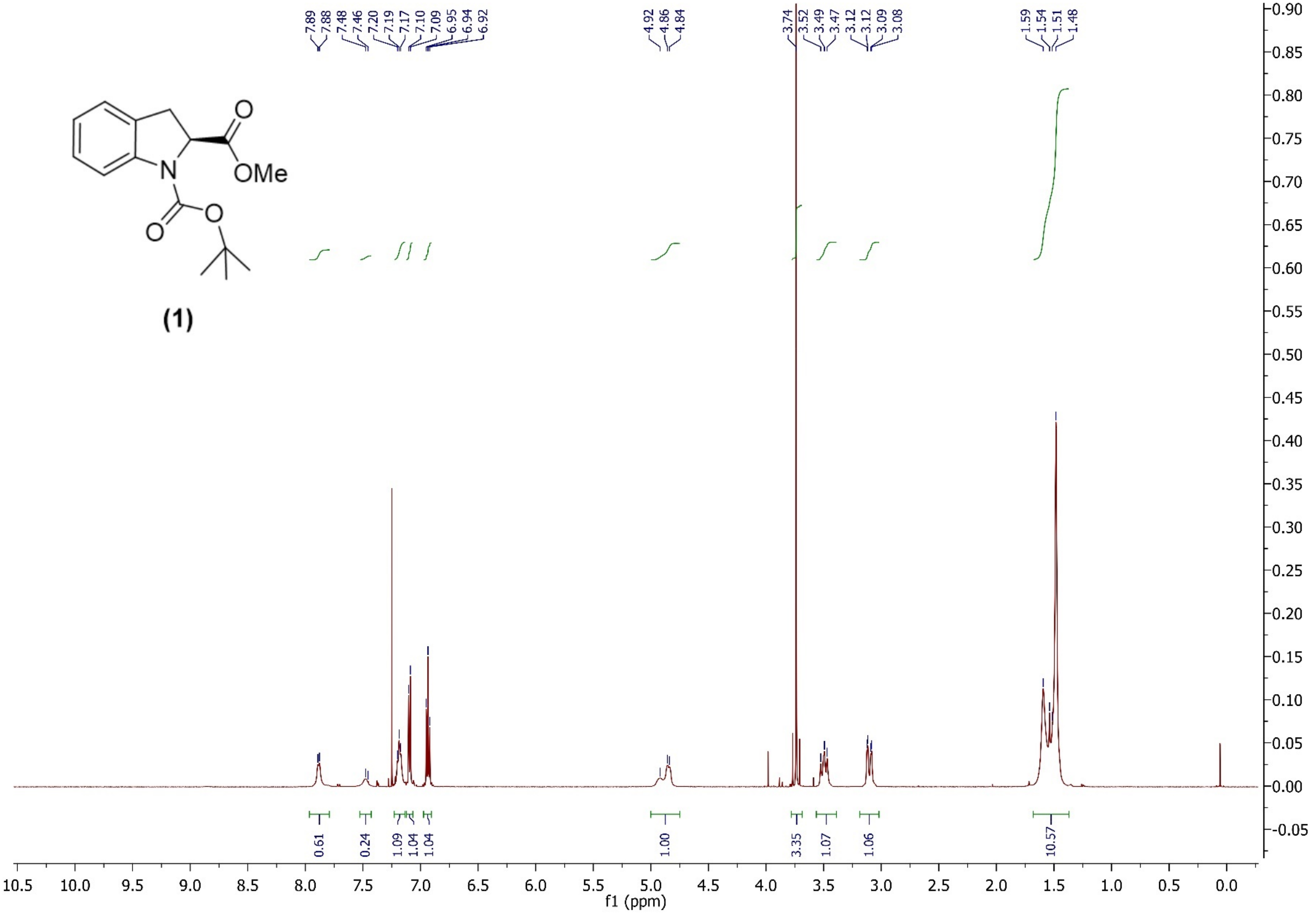

^{1}H NMR spectrum of Boc-(2*S*)-Ind-OMe (**1**) in DMSO-d_6 (0.1 M) at 500 MHz

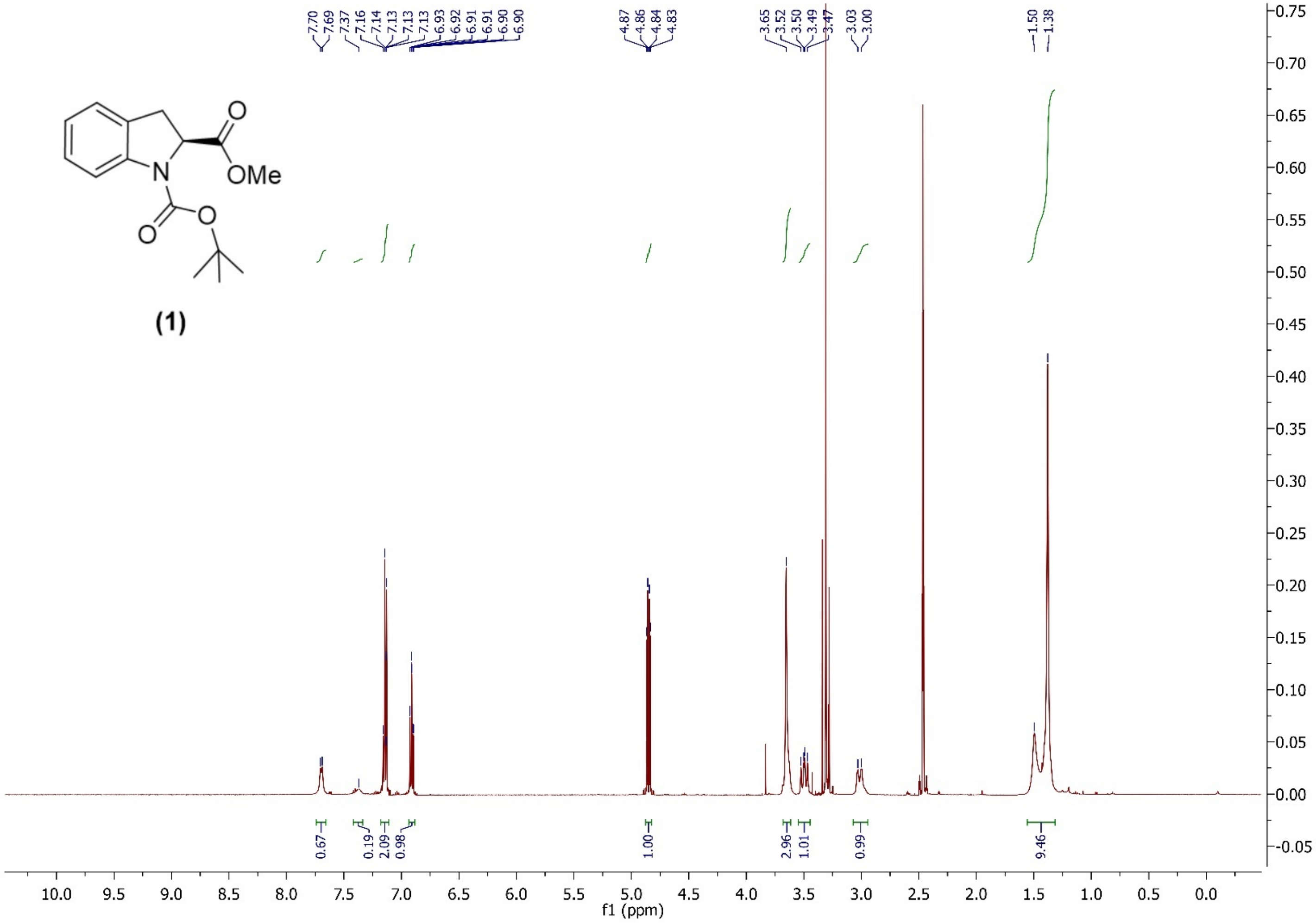

$^{13}C\{^1H\}$NMR spectrum of Boc-(2*S*)-Ind-OMe (**1**) in DMSO-d_6 at 125 MHz

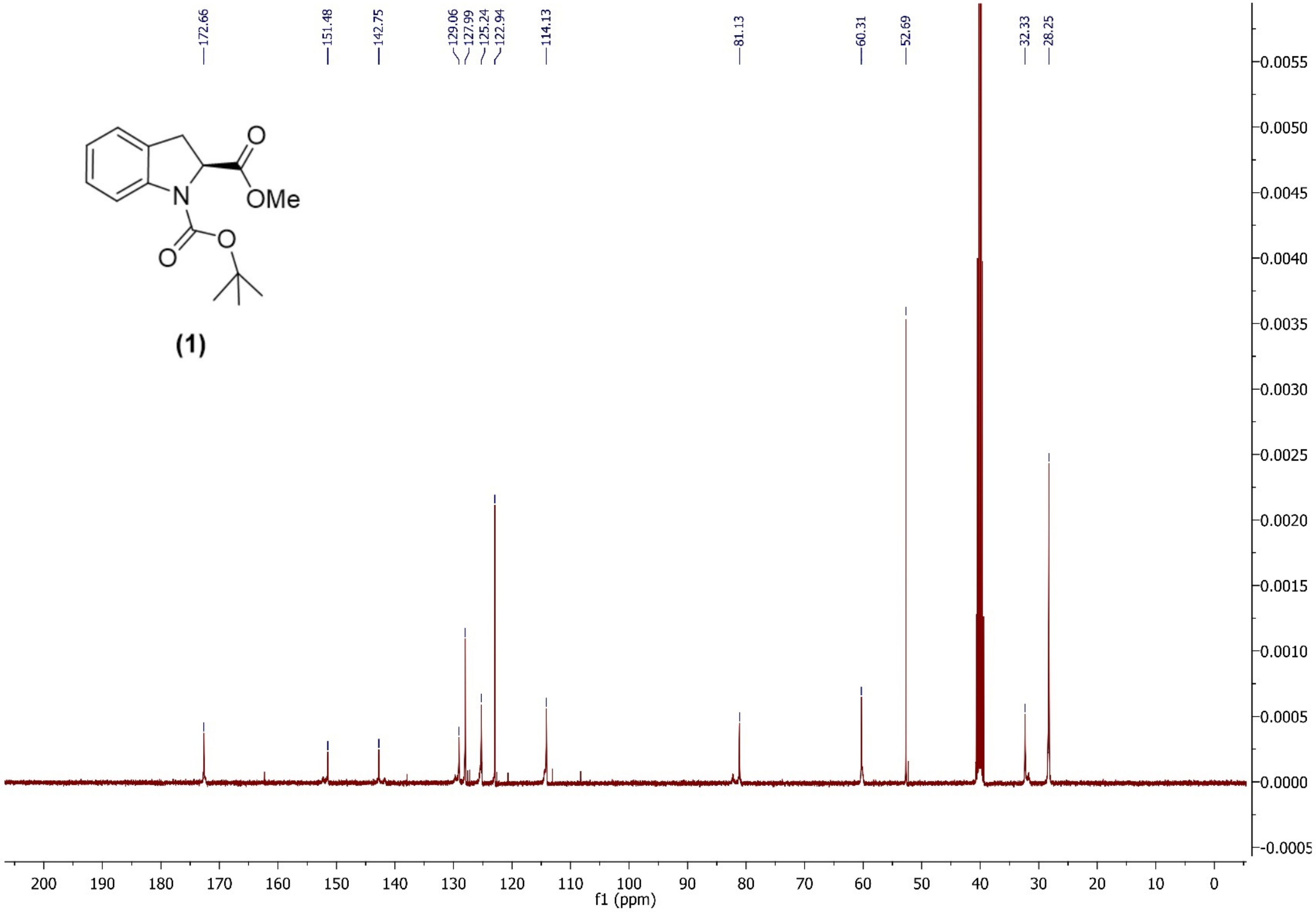

^{1}H NMR spectrum of Boc-((2*S*)-Ind)$_2$-OMe (**2**) in $CDCl_3$ (0.1 M) at 500 MHz

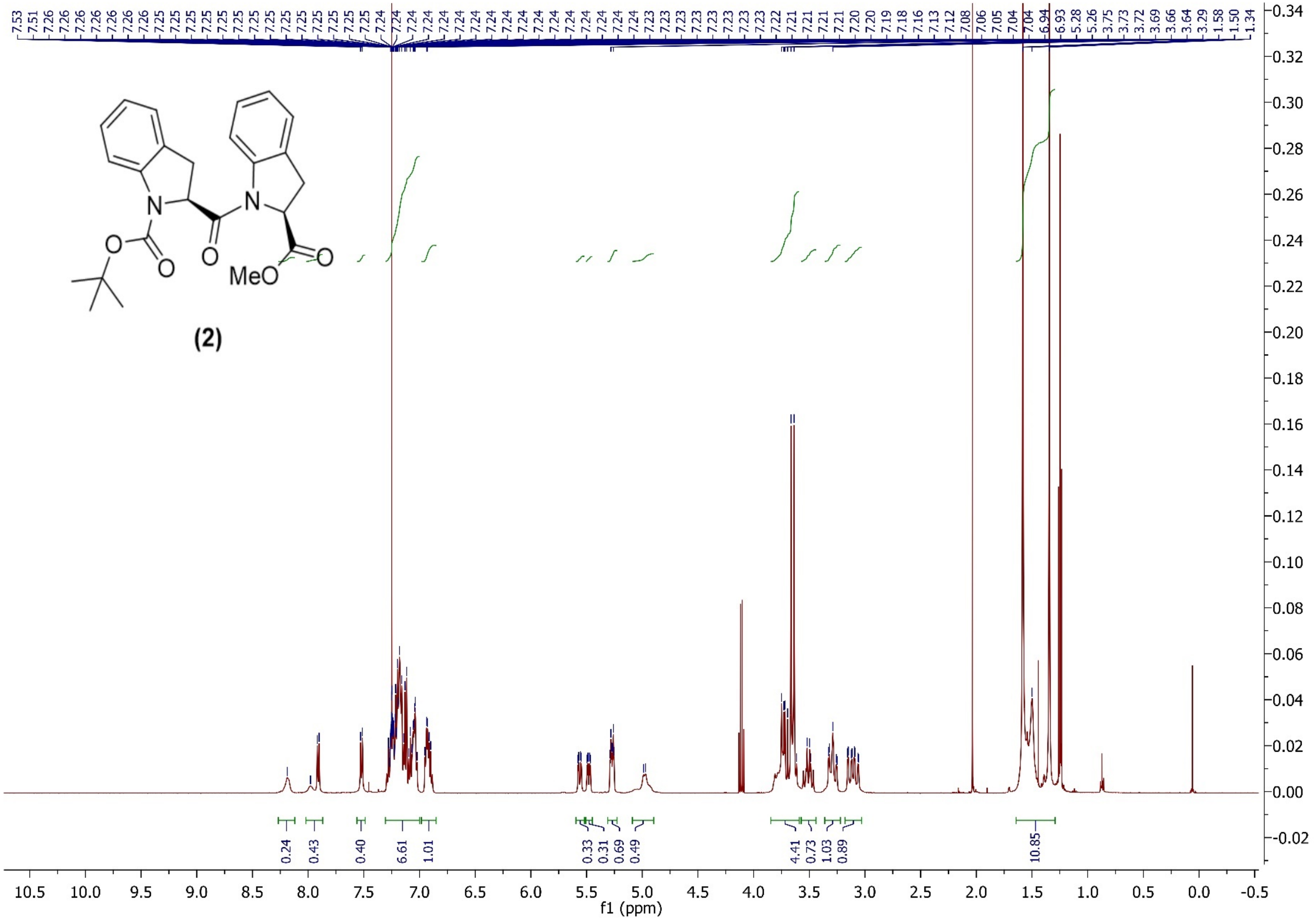

(2)

^1H NMR spectrum of Boc-((2*S*)-Ind)$_2$-OMe (**2**) in DMSO-d_6 (0.1 M) at 500 MHz

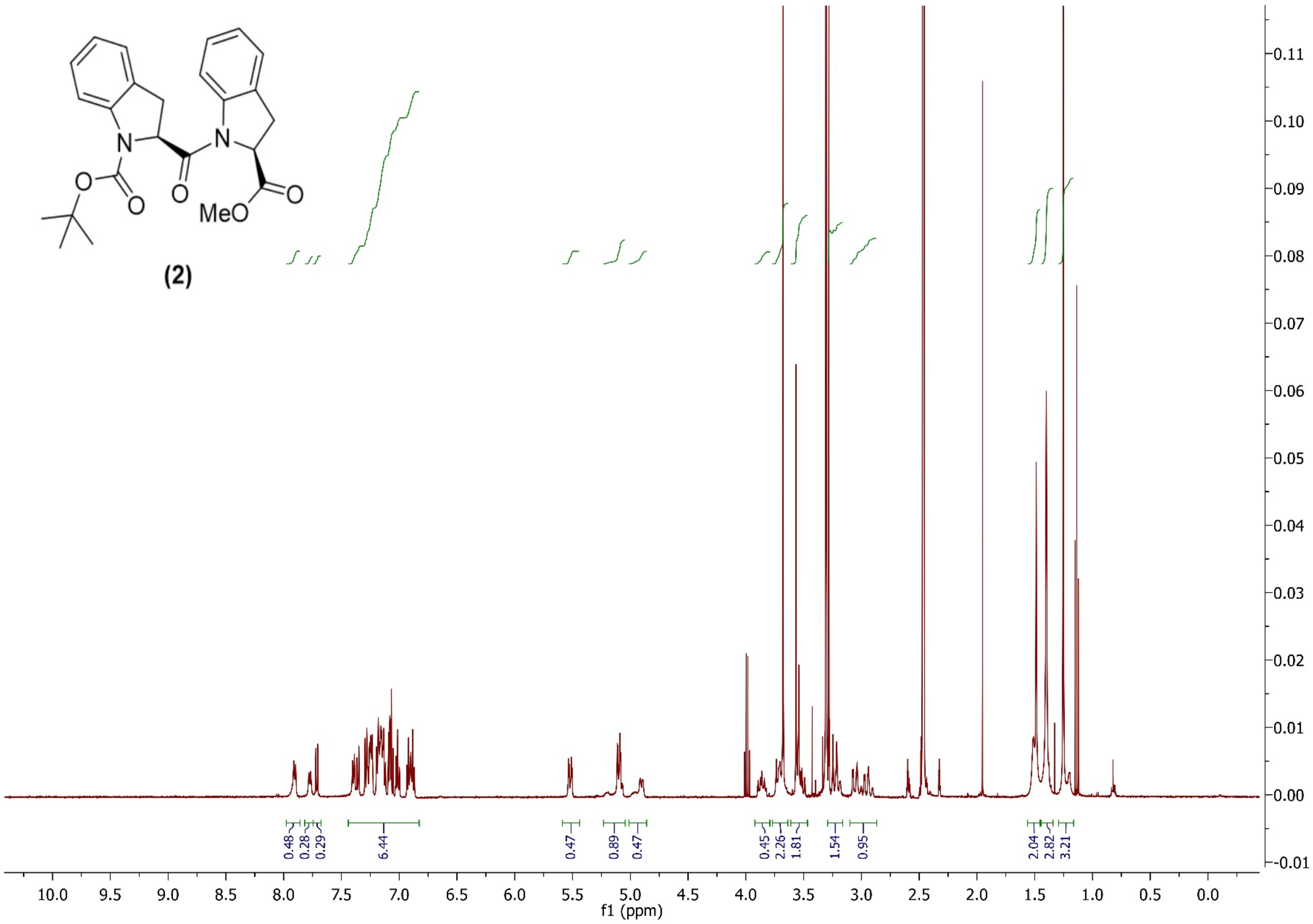

$^{13}C\{^1H\}$NMR spectrum of Boc-((2*S*)-Ind)$_2$-OMe (**2**) in DMSO-d_6 at 150 MHz

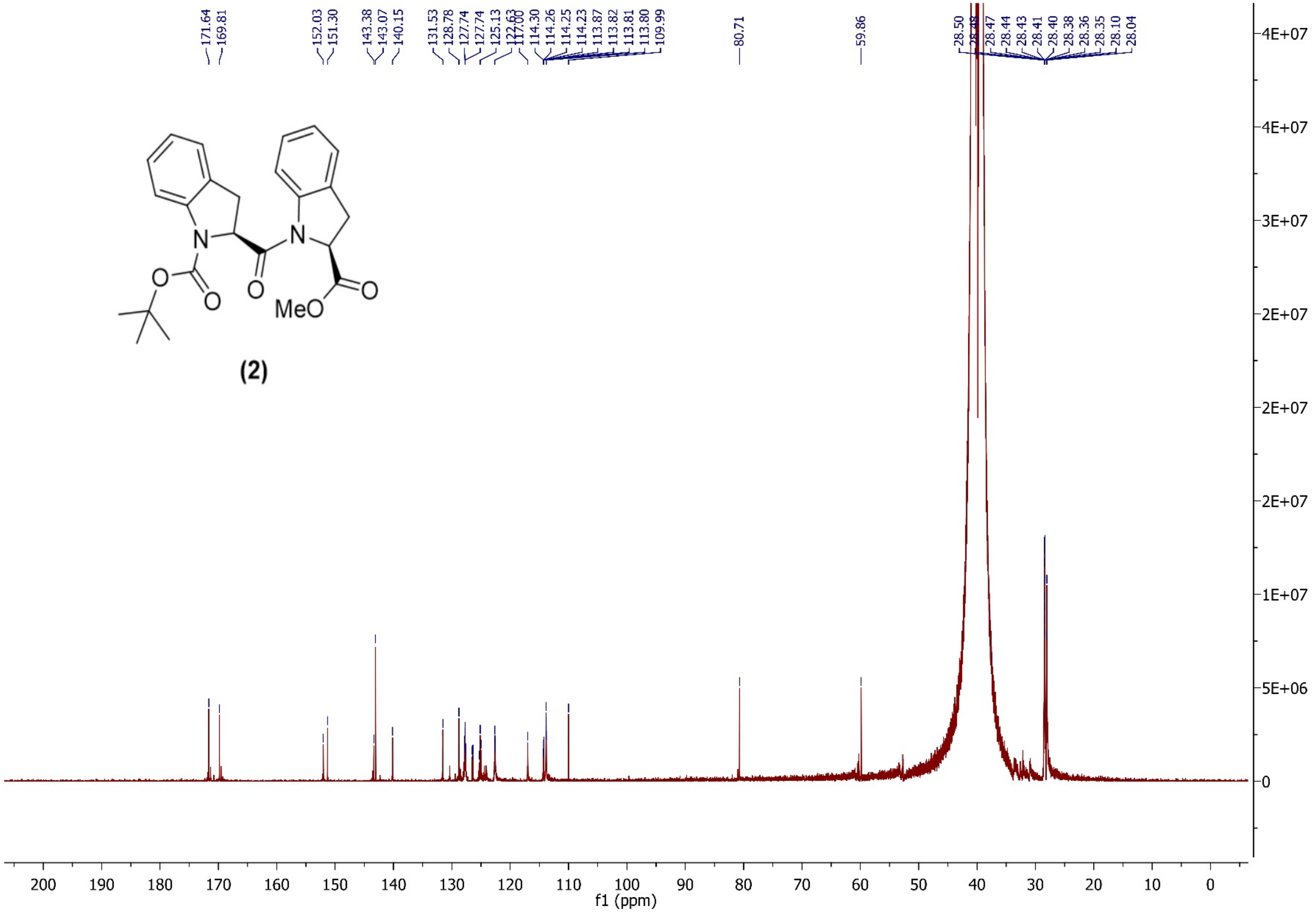

^{1}H NMR spectrum of Boc-((2*S*)-Ind)$_3$-OMe (**3**) in $CDCl_3$ (0.1 M) at 500 MHz

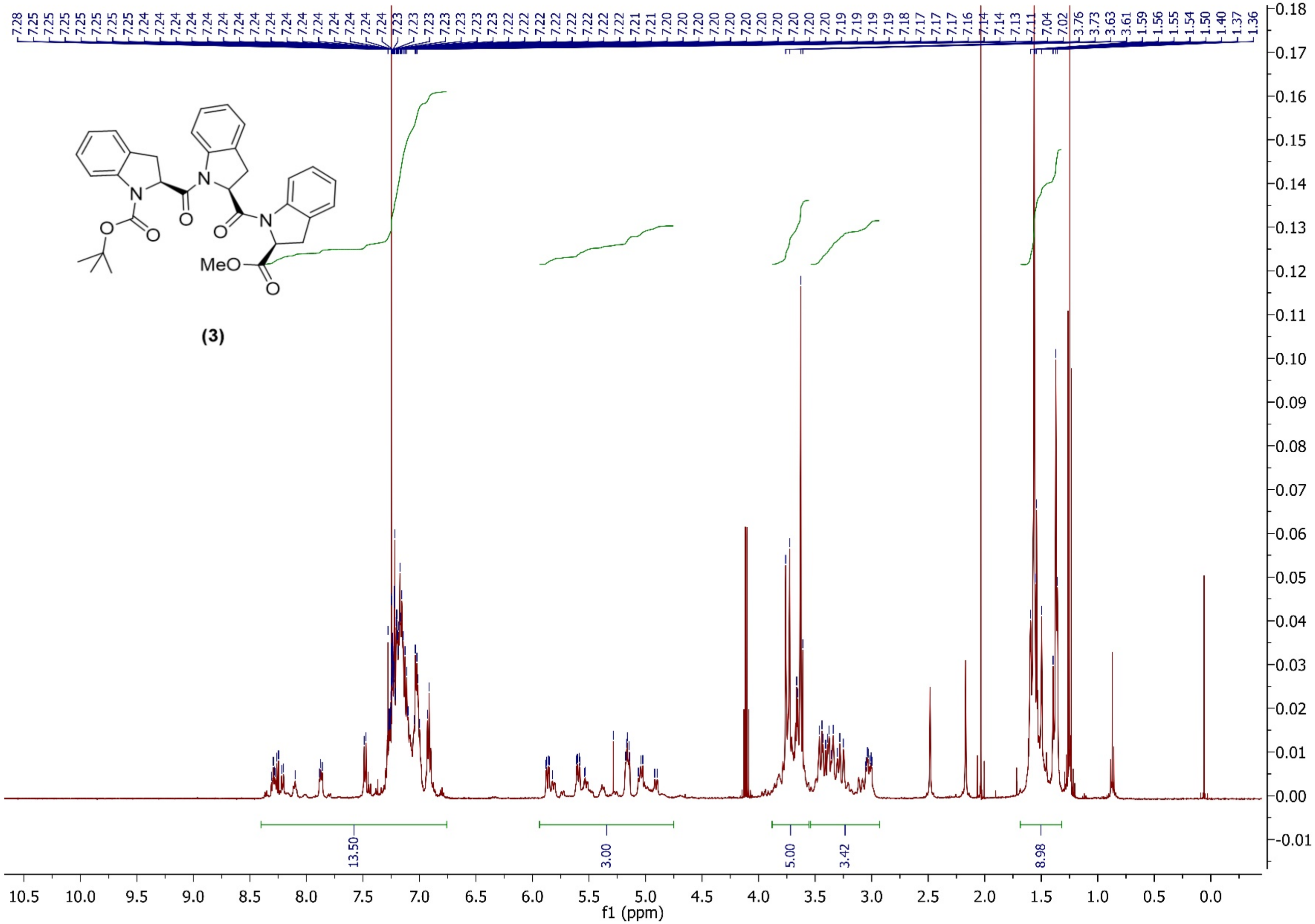

^{1}H NMR spectrum of Boc-((2*S*)-Ind)$_3$-OMe (**3**) in DMSO-d_6 (0.1 M) at 500 MHz

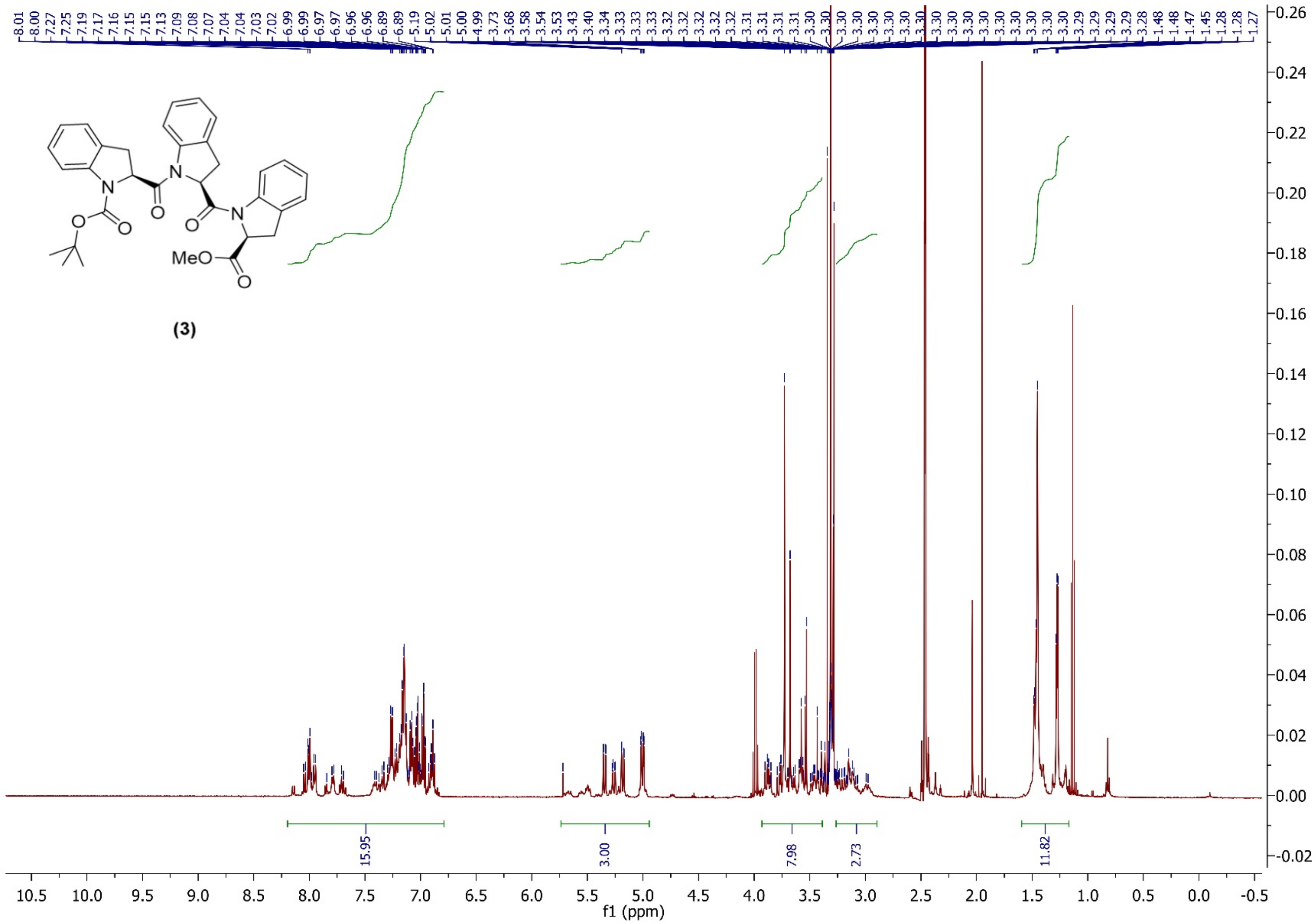

$^{13}C\{^{1}H\}$NMR spectrum of Boc-((2*S*)-Ind)$_3$-OMe (**3**) in DMSO-d_6 at 125 MHz

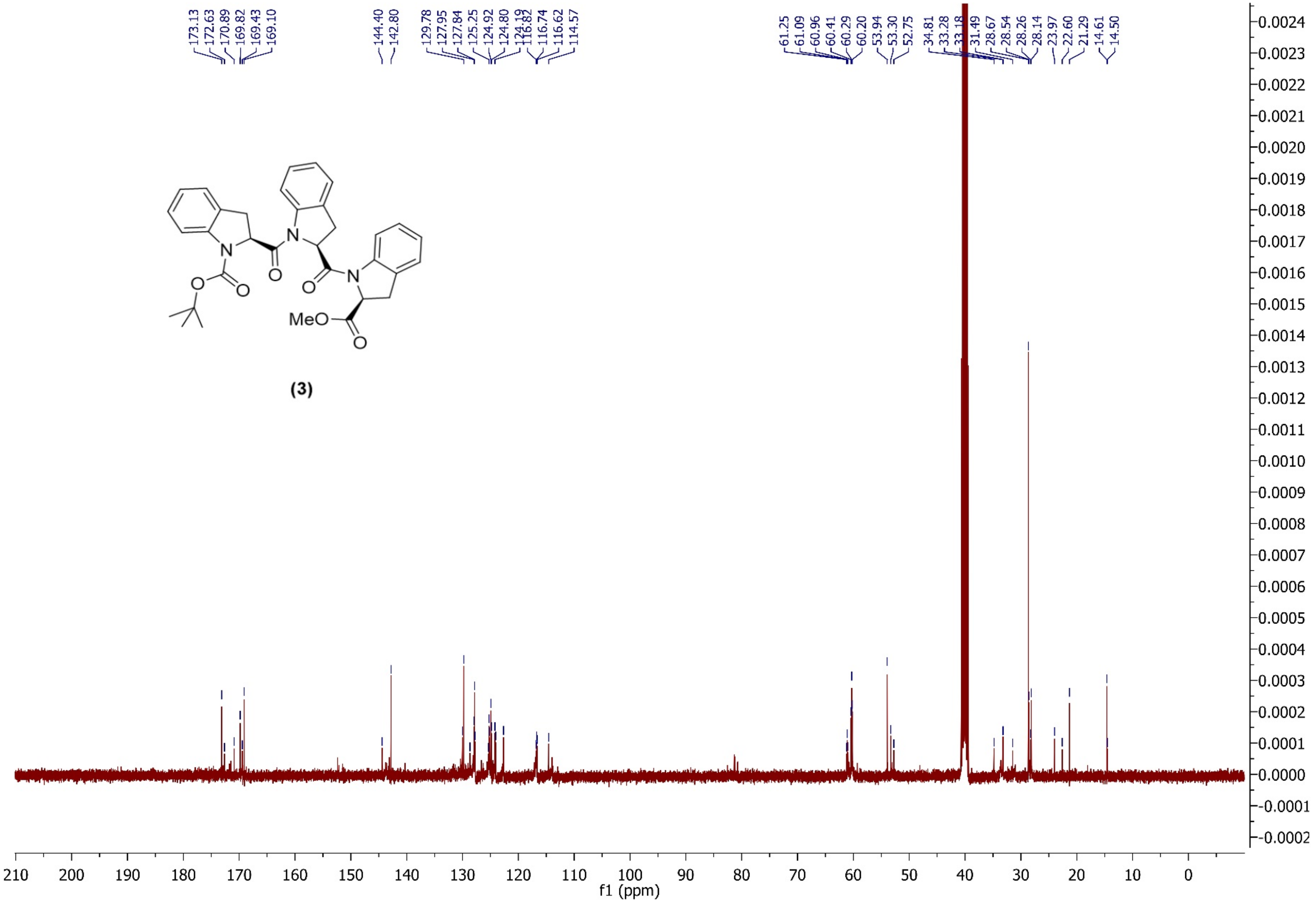

^{1}H NMR spectrum of Boc-((2*S*)-Ind)$_4$-OMe (**4**) in $CDCl_3$ (0.1 M) at 500 MHz

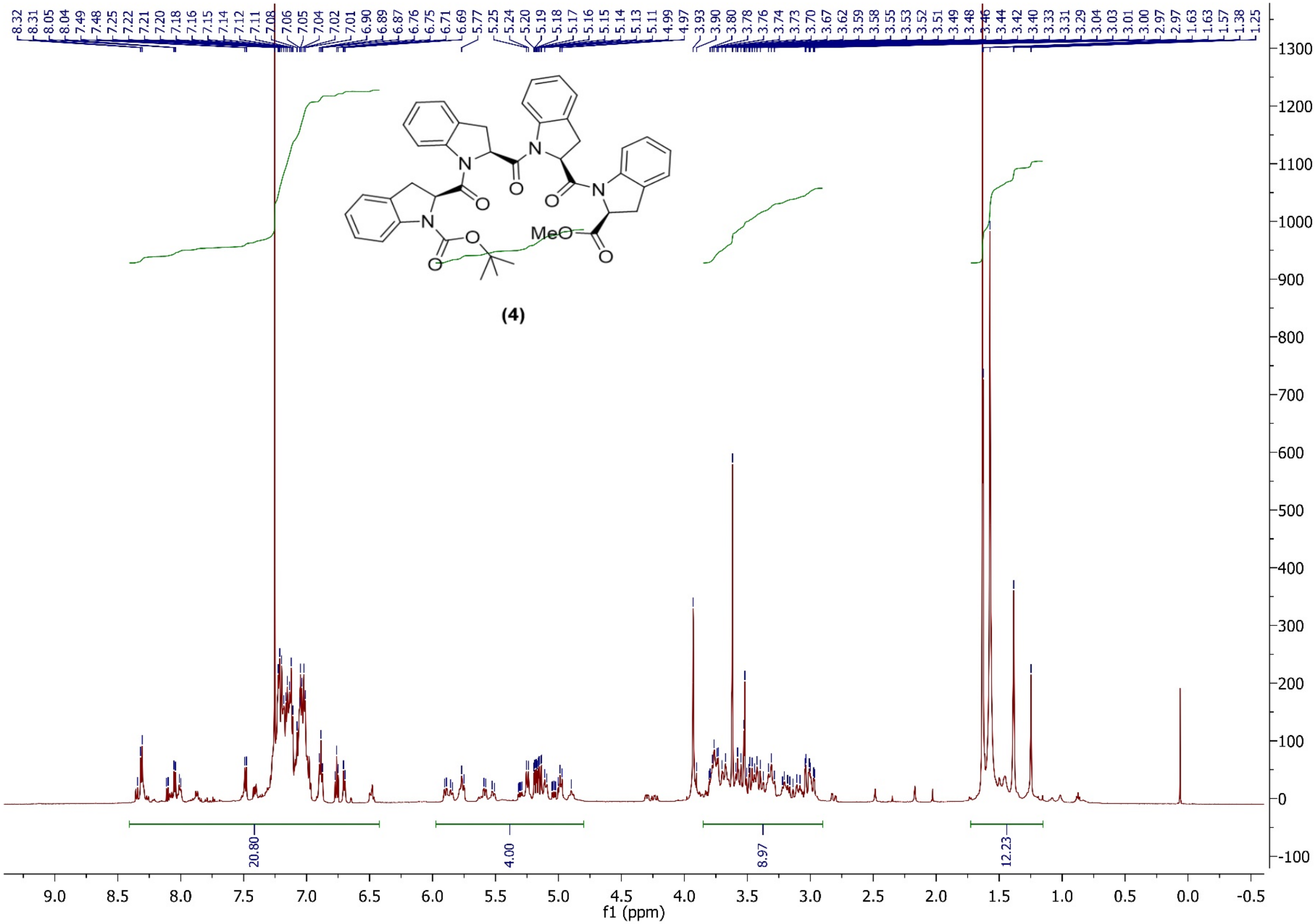

^{1}H NMR spectrum of Boc-((2*S*)-Ind)$_4$-OMe (**4**) in DMSO-d_6 (0.1 M) at 500 MHz

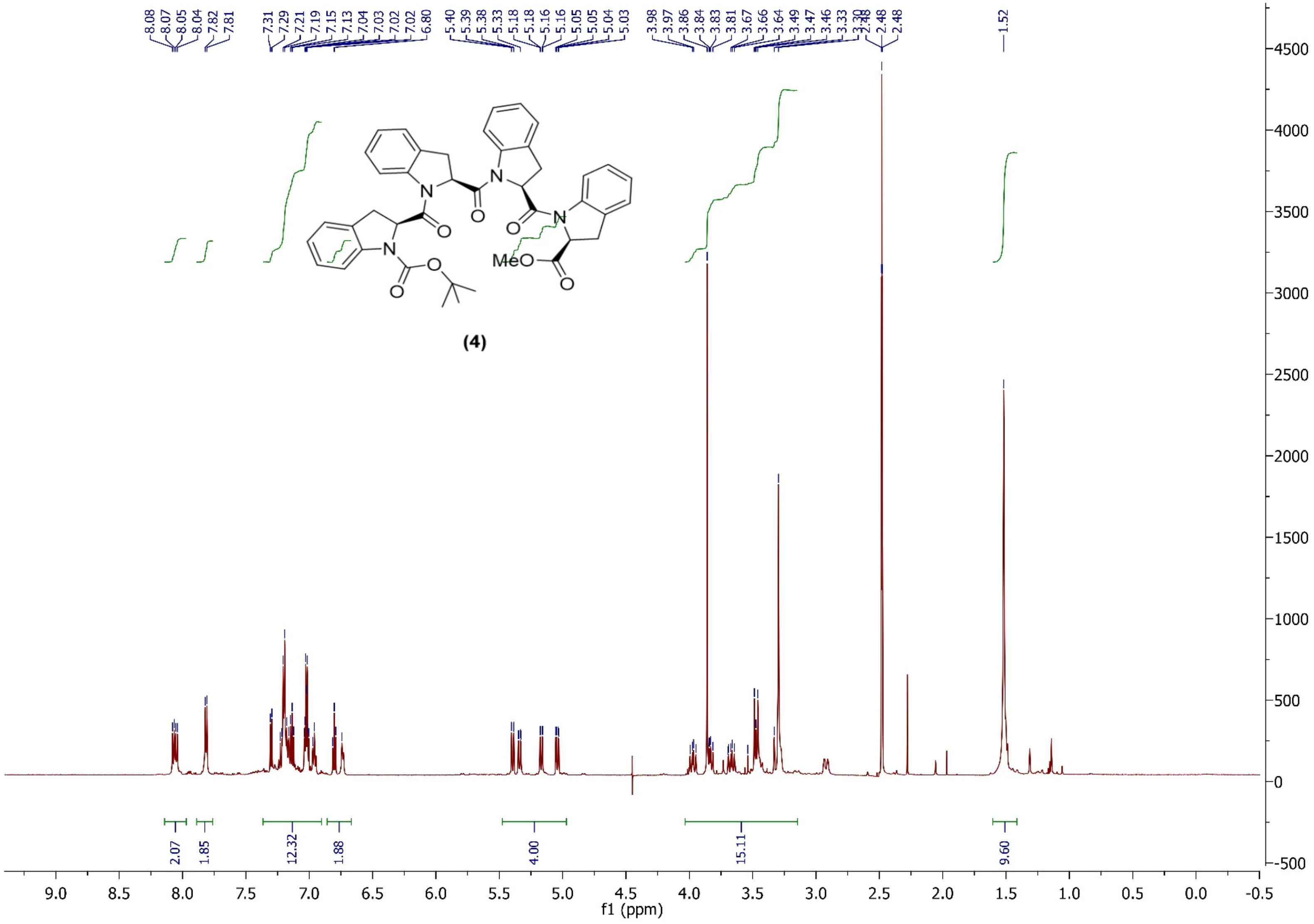

$^{13}C\{^{1}H\}$NMR spectrum of Boc-((2*S*)-Ind)$_4$-OMe (**4**) in DMSO-d_6 at 150 MHz

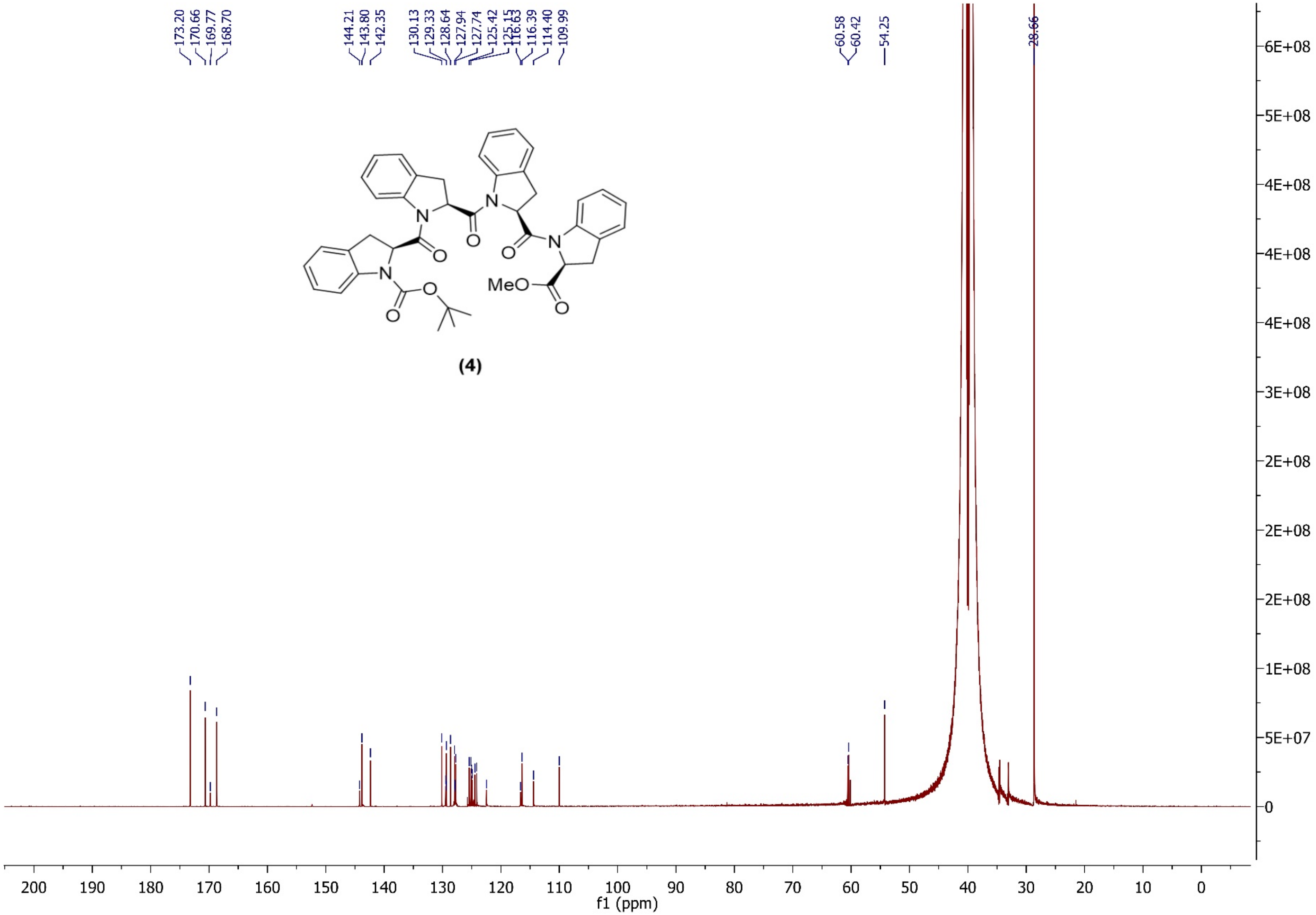

2D HSQC (600 MHz, DMSO-d6, 10 mM, 25 °C) map of Boc-((2*S*)-Ind)$_4$-OMe (**4**)

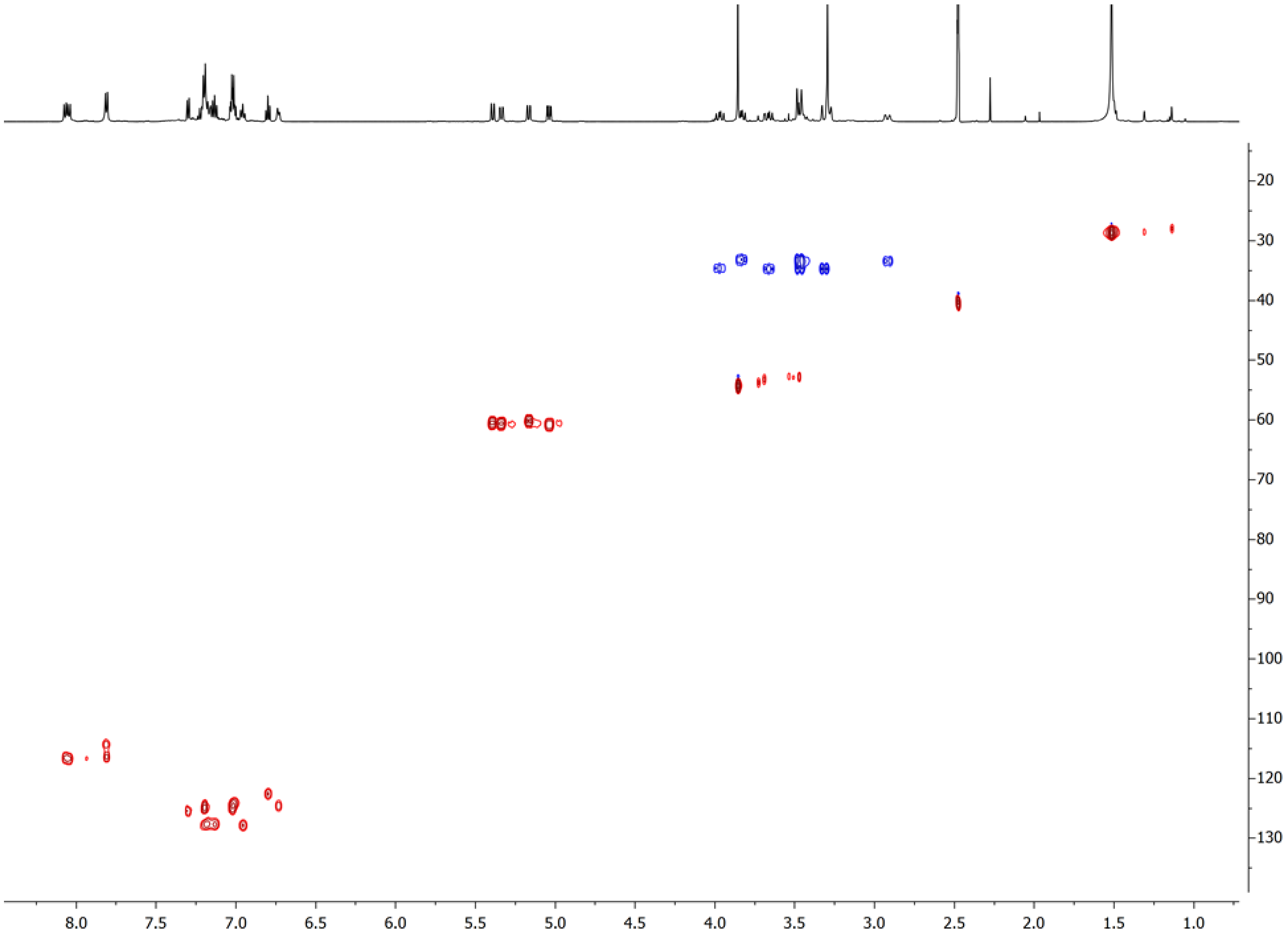

2D ROESY (600 MHz, DMSO-d6, 10 mM, 25 °C, mix 0.6 s) map of Boc-((2*S*)-Ind)$_4$-OMe (**4**)

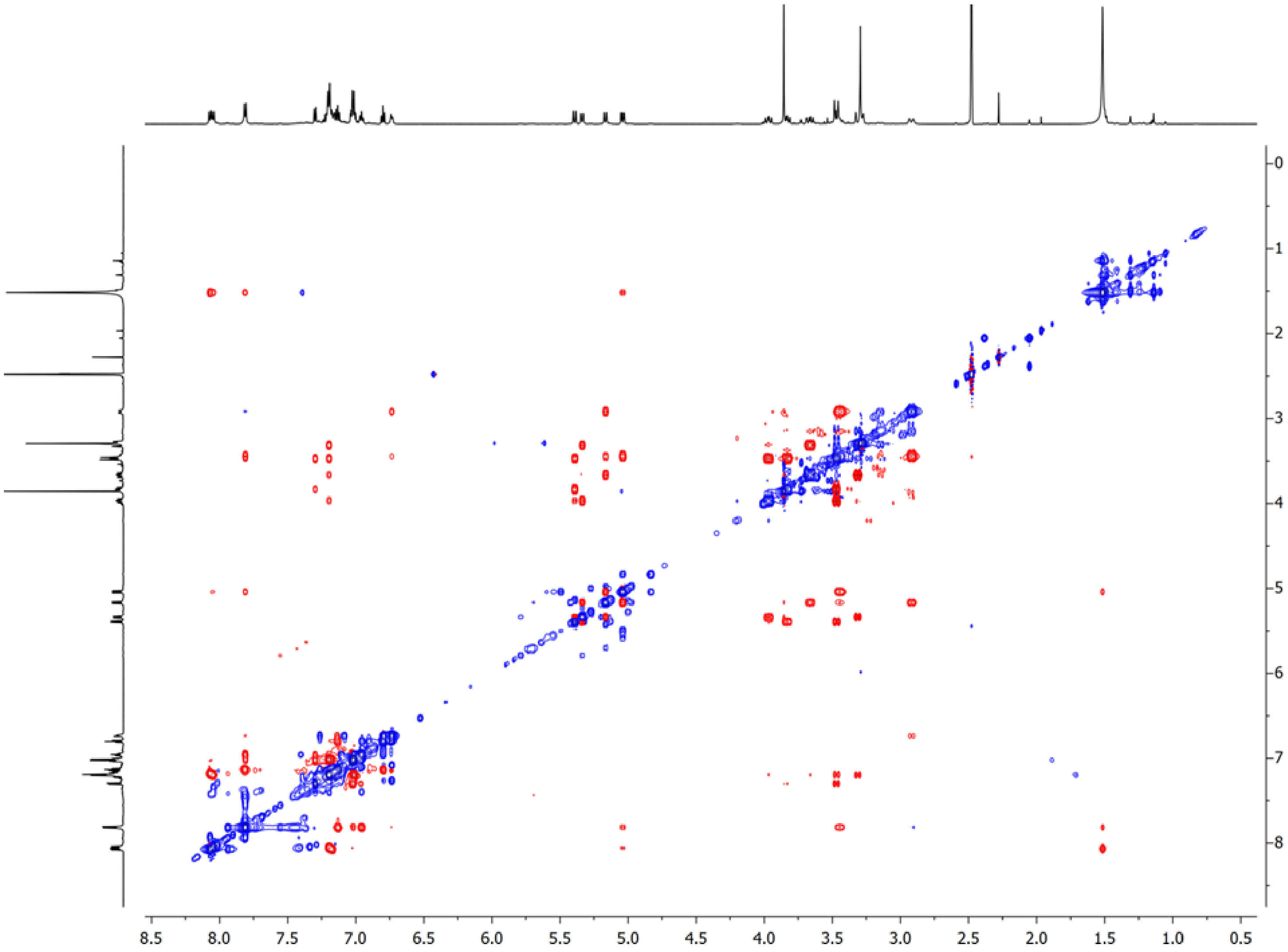

2D COSY (600 MHz, DMSO-d6, 10 mM, 25 °C) map of Boc-((2*S*)-Ind)$_4$-OMe (**4**)

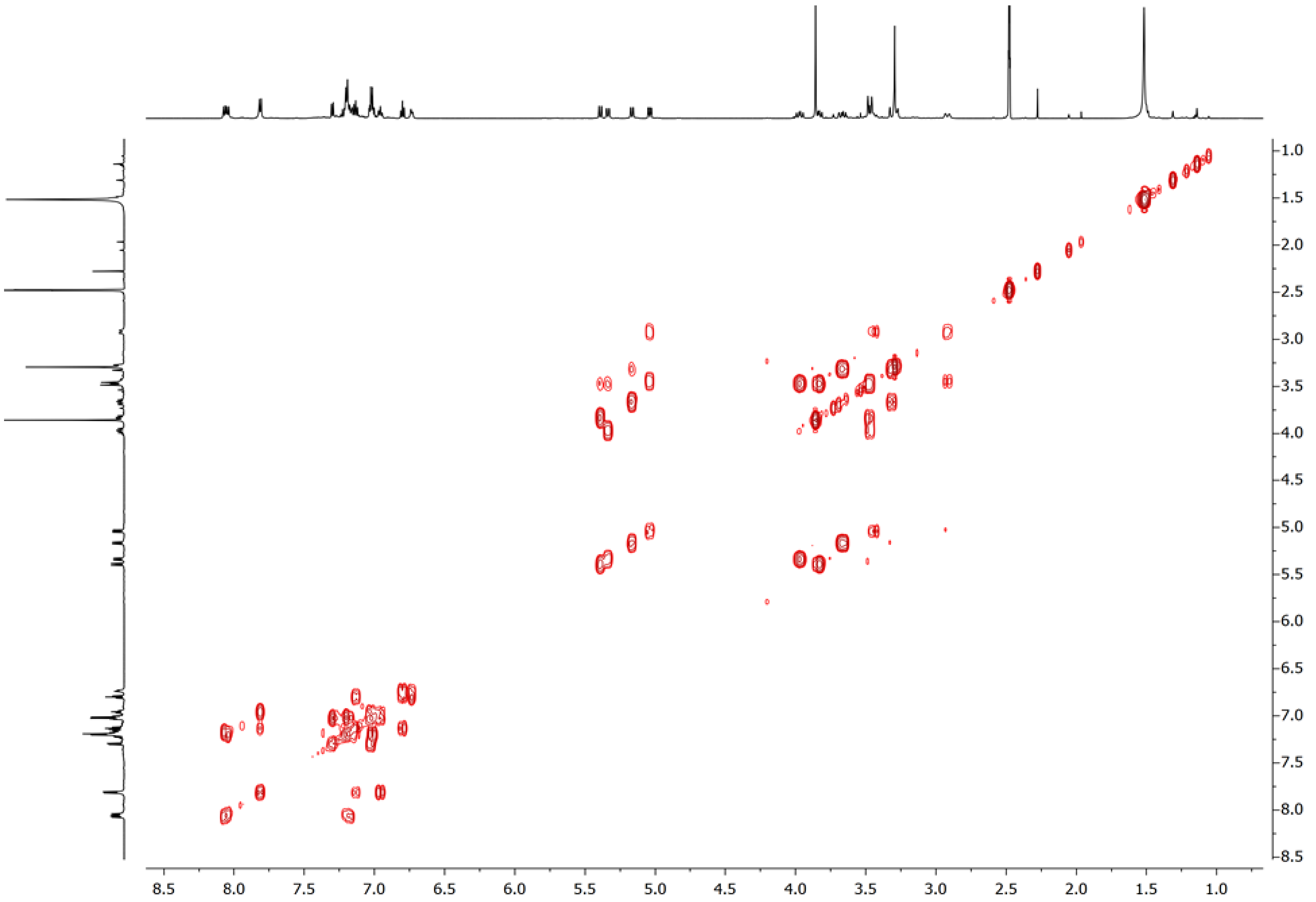

2D TOCSY (600 MHz, DMSO-d6, 10 mM, 25 °C) map of Boc-((2*S*)-Ind)$_4$-OMe (**4**)

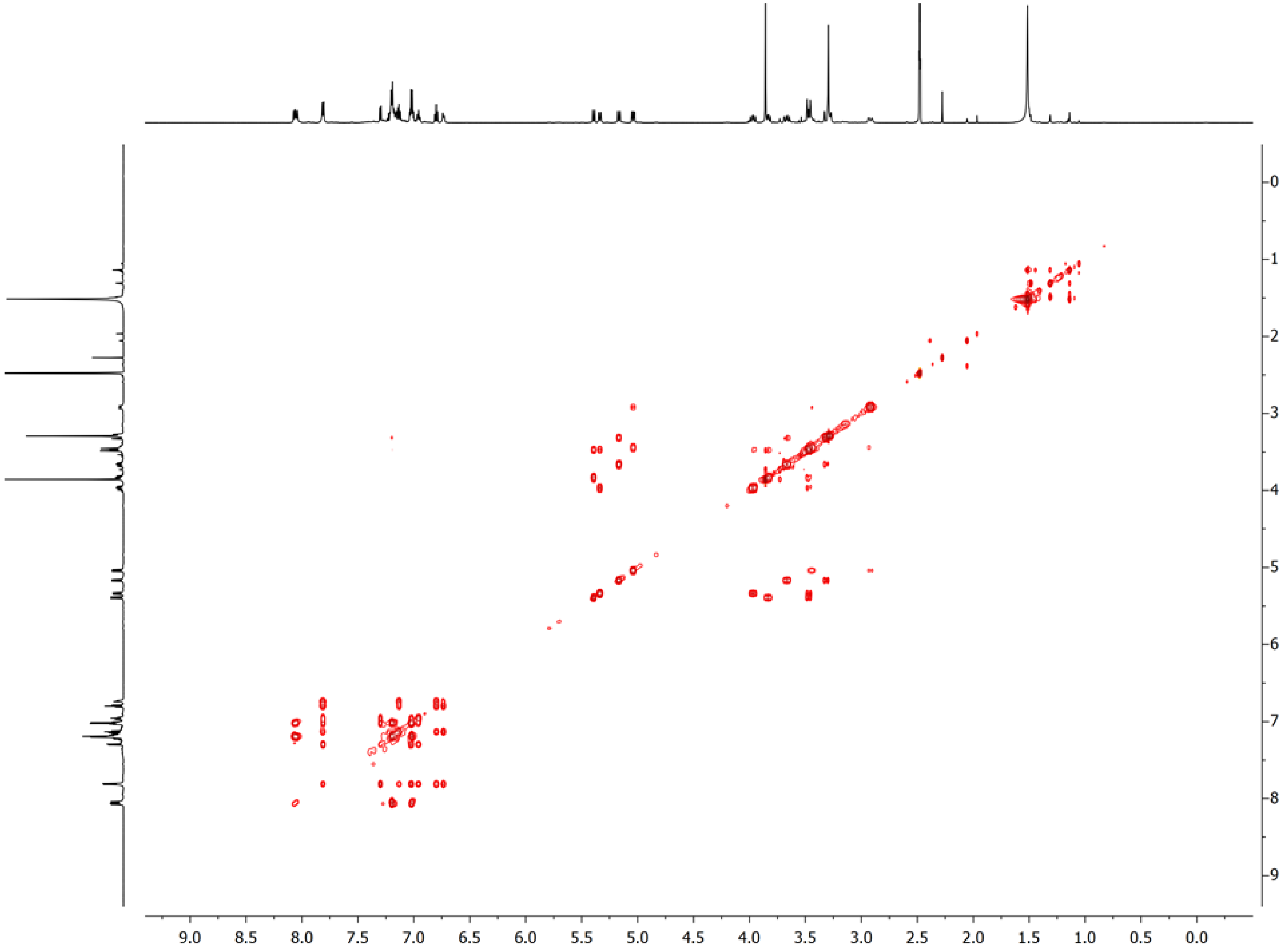

HPLC-MS analysis of (1)

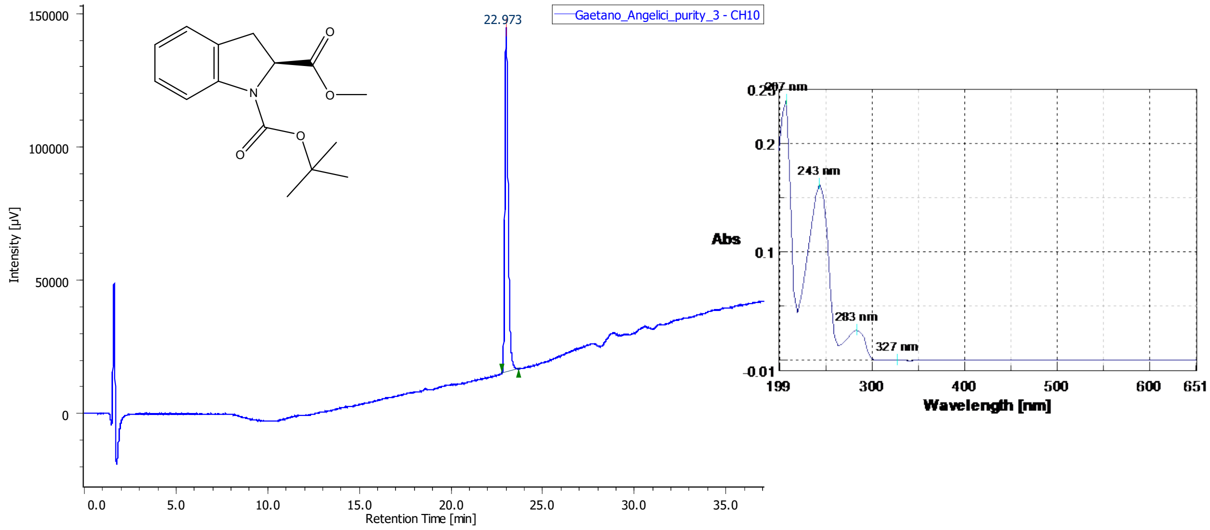

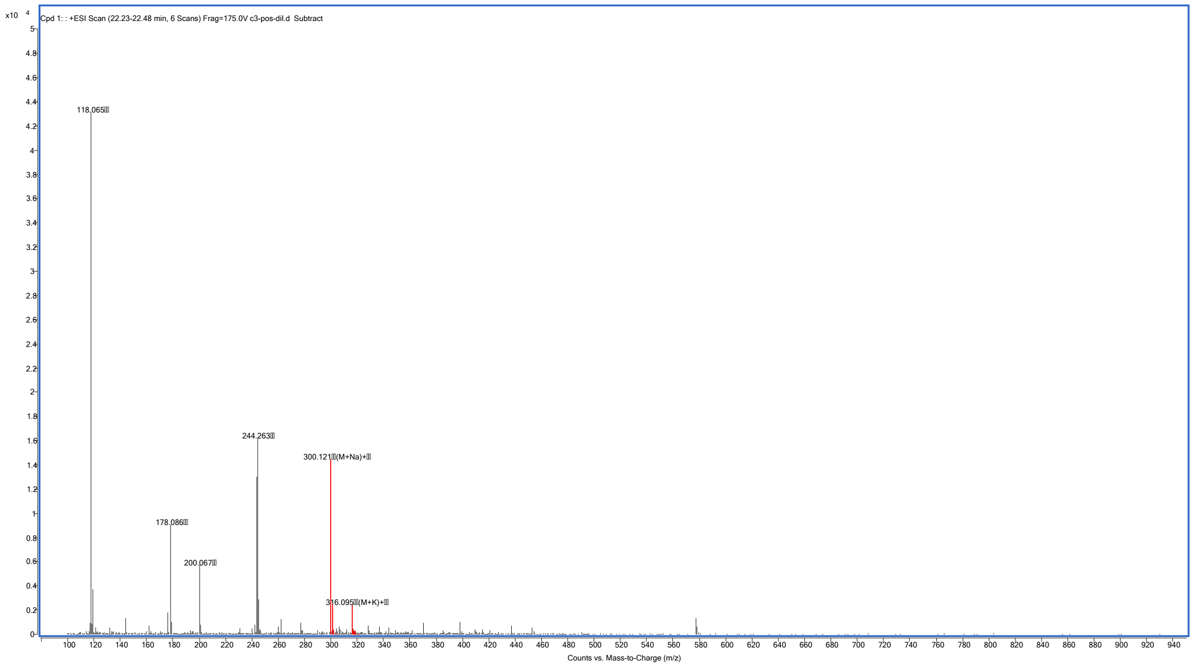

Sample name	Formula	Base peak (ion)	*m/z*		Diff (ppm)
			exact mass	experimental mass	
**1**	C15H19NO4	**$[M+K]^+$**	316.0946	316.0952	2.0

HPLC-MS analysis of (**2**)

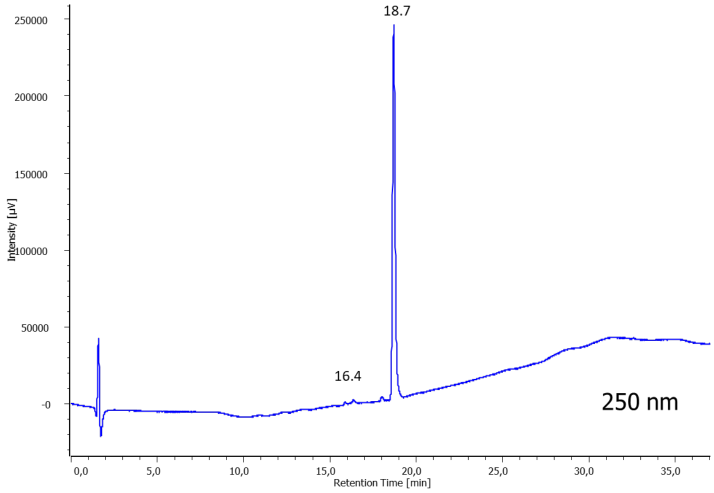

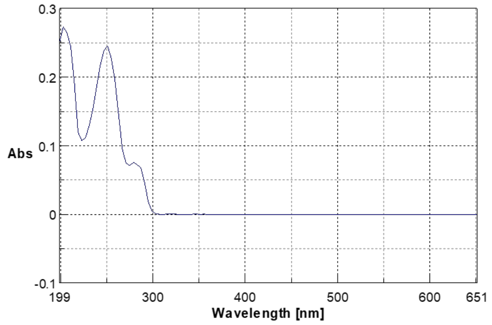

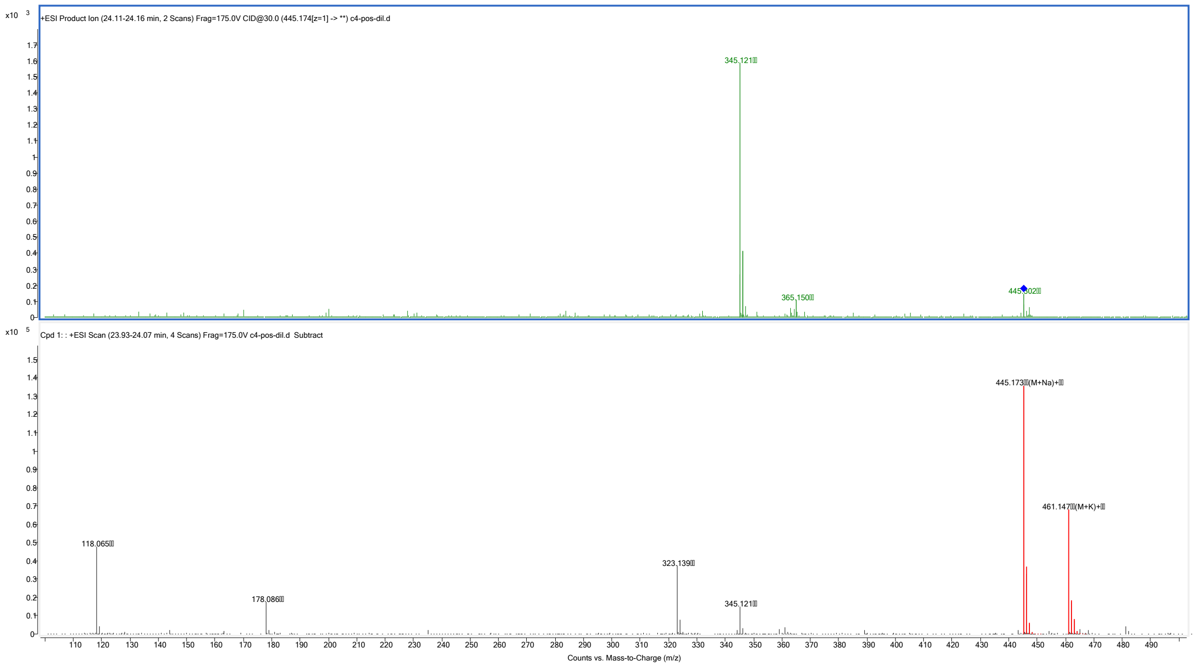

<table>
<tr><th rowspan="2">Sample name</th><th rowspan="2">Formula</th><th rowspan="2">Base peak (ion)</th><th colspan="2">m/z</th><th rowspan="2">Diff (ppm)</th></tr>
<tr><th>exact mass</th><th>experimental mass</th></tr>
<tr><td>2</td><td>C24H26N2O5</td><td>[M+Na]⁺</td><td>445.1734</td><td>445.1733</td><td>0.2</td></tr>
</table>

HPLC-MS analysis of (**3**)

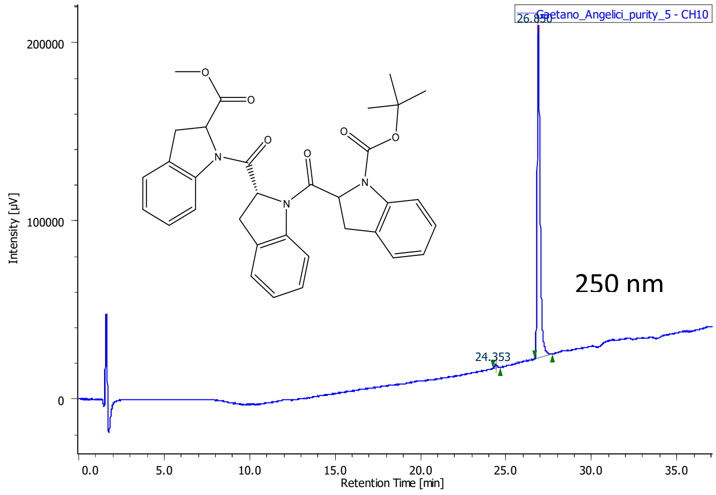

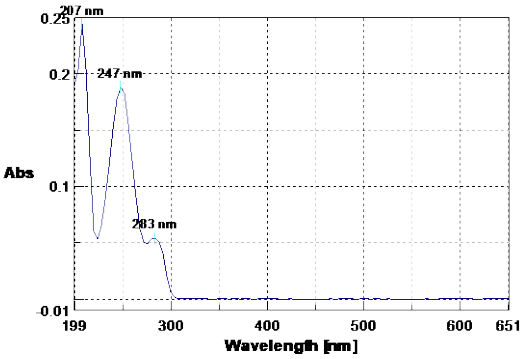

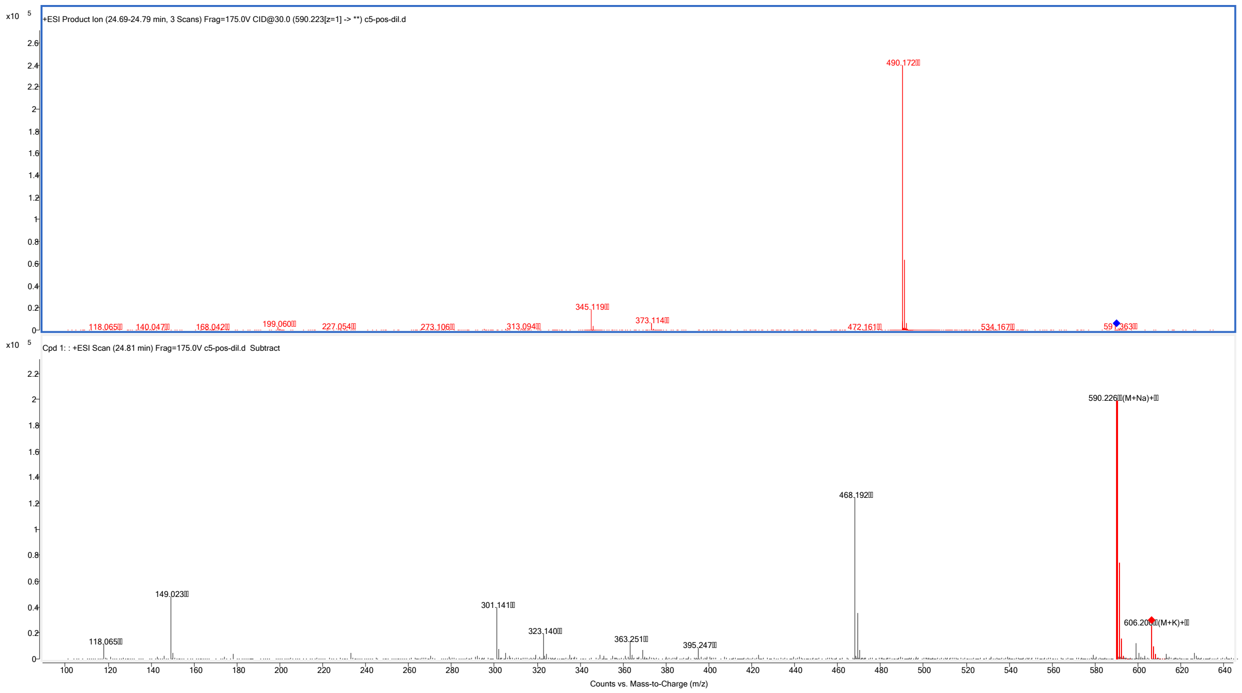

Sample name	Formula	Base peak (ion)	*m/z*		Diff (ppm)
			exact mass	experimental mass	
**3**	C33H33N3O6	**[M+K]$^{+}$**	606.2001	606.1999	0.32

HPLC-MS analysis of (**4**)

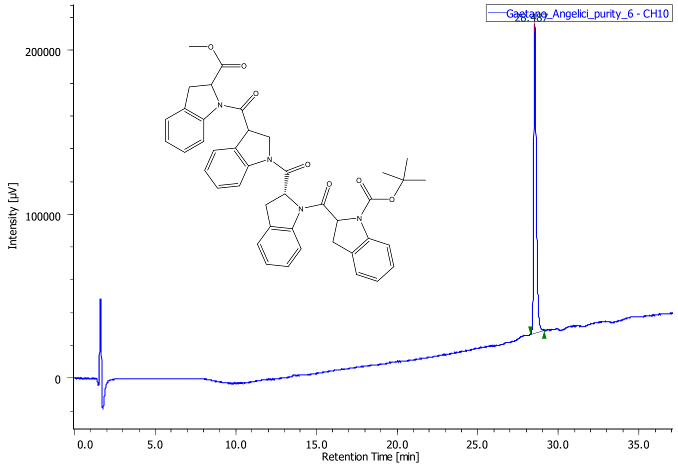

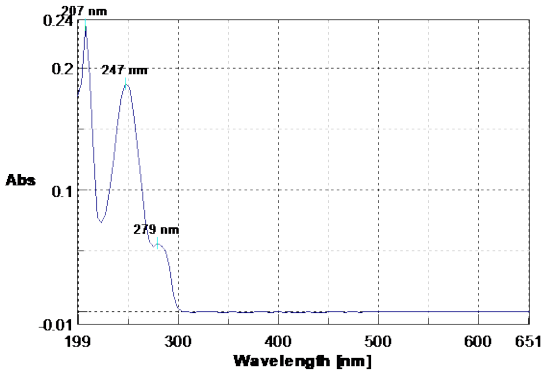

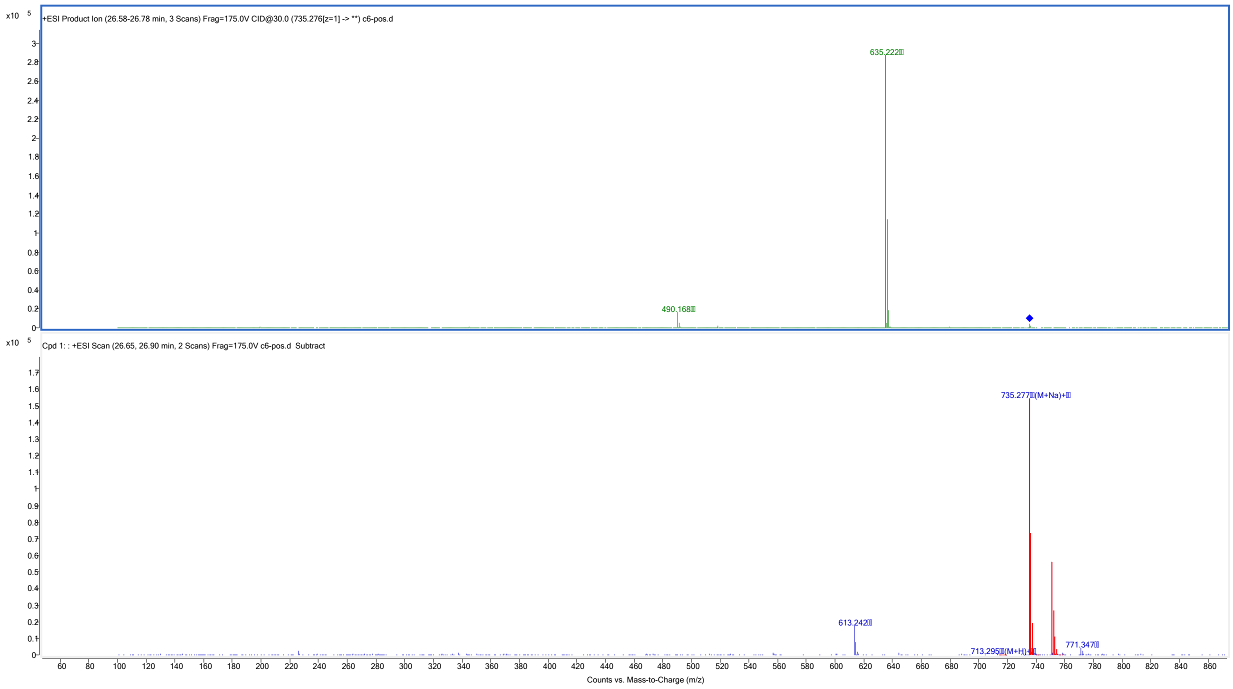

Sample name	Formula	Base peak (ion)	*m/z*		Diff (ppm)
			exact mass	experimental mass	
**4**	$C_{42}H_{40}N_4O_7$	$[M+Na]^+$	735.2789	735.2773	2.2

Table S1. Crystal data and structure refinement of (**4**)

CCDC number	2173109
Empirical formula	$C_{42}H_{40}N_4O_7$
Formula weight	712.78
Crystal system	Monolinic
Space group	$\boldsymbol{P}\,2_1$
a (Å)	10.4762(8)
b (Å)	9.2900(7)
c (Å)	19.1539(13)
β (°)	100.148(3)
Volume (Å^3)	1835.0(2)
Z	2
ρ_{calc} (g cm^{-1})	1.290
μ (mm^{-1})	0.089
F(000)	752
θ range (°)	3.0 to 26.5
Reflections collected	25312
Independent reflections	7460 (R_{int} = 0.0162)
Goodness-of-fit on F^2	1.041
Final R_1 [$I \geq 2\sigma(I)$]	0.0404
Final wR_2 [$I \geq 2\sigma(I)$]	0.1093
Final R_1 [all data]	0.0432
Final wR_2 [all data]	0.1124

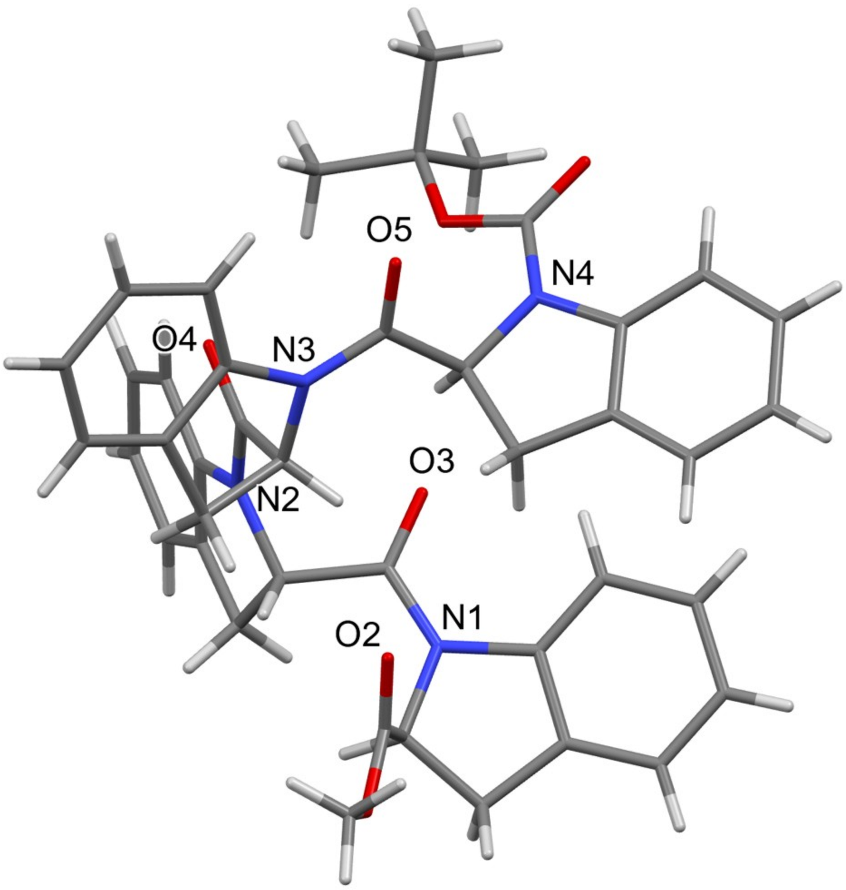

Table S2. Description of main C–H···O interactions

Along the peptide chain, which shows a helical conformation, the peptide groups alternate with the residues of the indoline-2-carboxylic acid which show CO–N–C–CO torsion angles ranging between 70.8 and 85.9 degrees. The peptide groups are essentially planar due both to the resonance effect and to hydrogen interactions with the CH group in position 7 of the indole ring of proline. The strongest C–H···O interactions are listed in the Table and illustrated in the Figure below.

Groups	C···O distance / Å	C–H···O angle / deg
C42–H42B···O6	2.840(8)	120.7
C18–H18···O4	2.853(3)	116.1
C36–H36···O6	2.862(5)	115.2
C27–H27···O5	2.868(4)	115.2
C9–H9···O3	2.903(4)	115.6

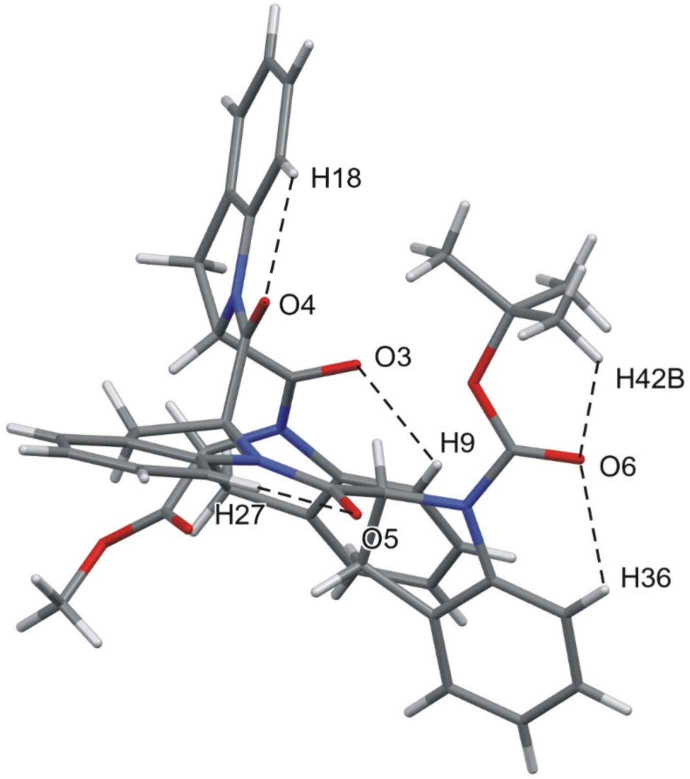

Table S3. Calculated energy differences between conformers

Energies and dihedral angles for the conformations extracted from the PBMetaD simulations in DMSO and chloroform. Some conformations were found in both solvents, while most of them are found only in one of the solvents. The dihedral angles and molecular dipole refer to the DFT-optimized structure (in DMSO where present, otherwise in chloroform). The number of amide bonds in *cis* conformation is also reported.

**Conf.**	**N. cis**	**ω A**	**ω B**	**ω C**	**ω D**	**ψ B**	**ψ C**	**ψ D**	**Solv.**	**G(DMSO)**	**G(CHCl3)**	**Dipole (D)**
**0**	3	2	-3	-17	-176	161	145	157	DMSO	3.35		12.4
**1**	4	3	-11	-13	4	164	160	160	both	0.9	2.78	20.7
**2**	1	164	-176	-14	176	165	110	174	DMSO	4.85		6.6
**3**	3	5	-173	-8	-8	145	116	168	both	5.23	5.98	5.1
**4**	2	-11	179	174	-3	159	172	146	CHCl3		7.05	6.1
**5**	2	11	2	-176	-172	-31	161	146	CHCl3		1.46	3.0
**6**	3	0	-14	-28	178	-32	-17	170	DMSO	2.76		8.8
**7**	2	-13	-172	-17	-178	158	108	174	DMSO	2.81		4.2
**8**	3	-3	-12	-178	-8	-31	166	144	CHCl3		1.02	3.6
**9**	2	-164	-7	-4	177	144	140	166	DMSO	4.12		13.3
**10**	2	-169	0	-178	-15	-31	166	105	CHCl3		5.82	4.7
**11**	3	178	4	13	11	156	-26	169	CHCl3		8.7	6.8
**12**	1	-172	2	-177	-172	-30	162	146	CHCl3		2.82	7.8
**13**	2	-163	34	-4	177	-18	-28	169	DMSO	3.73		5.4
**14**	3	-6	-10	-173	7	-35	174	-28	CHCl3		2.92	8.8
**15**	3	-9	-176	-1	11	150	141	-46	DMSO	1.81		6.4
**16**	3	-178	-7	-15	2	160	163	163	both	1.78	4.21	18.0
**17**	2	178	7	3	175	151	-36	167	both	0	0	5.6
**18**	2	-1	-163	-2	180	142	-35	168	DMSO	1.88		15.1

Torsional parameters.

The parameters for the dihedral ω were obtained by fitting a QM torsional profile. QM energies were obtained at the B3LYP/6-311G(d,p) level of theory by optimizing an indoline dimer with frozen ω dihedrals. The initial parameters for the torsion were taken from the Amber ff14SB force field, and fitted to the QM torsional profile. The final fit is reported in the figure below.

Figure: Final torsional profile obtained at the QM level of theory and at the MM level after fitting the dihedral parameters.

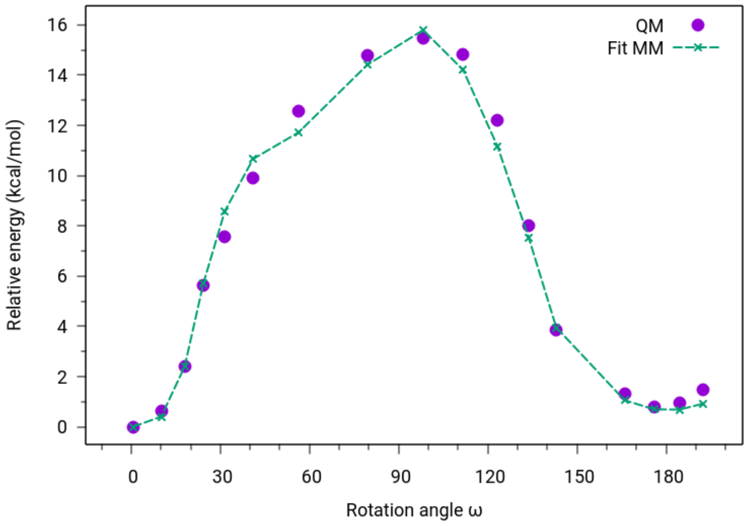

The fitted parameters are reported below in the Amber format:

CT-C	-N	-CT	1	0.33590000	180.000	-1.0
CT-C	-N	-CT	1	2.78650000	180.000	-2.0
CT-C	-N	-CT	1	0.23450000	0.000	-3.0
CT-C	-N	-CT	1	0.71300000	180.000	6.0
CT-C	-N	-CB	1	0.55290000	180.000	-1.0
CT-C	-N	-CB	1	2.30570000	180.000	-2.0
CT-C	-N	-CB	1	1.18040000	0.000	-3.0
CT-C	-N	-CB	1	0.20880000	0.000	6.0
O -C	-N	-CB	1	0.44680000	0.000	-1.0
O -C	-N	-CB	1	2.40780000	180.000	-2.0
O -C	-N	-CB	1	1.08870000	180.000	-3.0
O -C	-N	-CB	1	0.00710000	180.000	6.0
O -C	-N	-CT	1	0.43910000	0.000	-1.0
O -C	-N	-CT	1	2.55570000	180.000	-2.0
O -C	-N	-CT	1	0.40840000	180.000	-3.0
O -C	-N	-CT	1	0.19670000	180.000	6.0

Table S4. Calculated ROE distances along the PBMetaD in DMSO

Calculated distances in all the PBMetaD clusters for the inter-unit ROE interactions shown in **Figure 3** and **Figure SI1**. Distances were calculated for the structures belonging to each cluster in the PBMetaD. Each cluster is assigned the same conformation number as its representative in **Table SI3**. Within each cluster, each “average” distance was calculated by averaging $1/R^6$. Distances involving equivalent tBu atoms were averaged in the same way. A NOE violation was detected every time the average distance was above 4 Å.

Cluster	Dist. HAα - HBα	Dist. HBα - HCα	Dist. HCα-HDα	Dist. HAα - HD1	Dist. HAβ'-HBα	Dist. HBβ'-HCα	Dist. HCβ'-HDα	Dist. HC1-tBu	Dist. HB1-tBu	NOE Violations
**0**	2.52	2.66	4.49	6.07	2.52	2.48	4.69	3.82	3.91	3
**1**	2.45	2.43	2.51	3.68	2.71	2.54	2.45	3.81	3.83	0
**2**	4.53	2.09	4.42	7.64	4.51	3.24	4.65	11.42	7.39	7
**3**	4.39	2.43	2.52	6.81	4.88	2.52	2.43	8.90	5.06	5
**6**	3.86	3.94	4.48	5.88	3.45	3.70	4.67	8.90	4.55	5
**7**	4.54	2.07	4.44	7.51	4.60	3.56	4.72	9.78	5.40	7
**9**	2.32	2.54	4.52	6.16	2.86	2.48	4.76	4.98	6.81	5
**13**	3.73	3.84	4.41	6.23	3.24	3.96	4.85	8.09	7.14	5
**15**	4.32	2.52	3.97	7.25	5.02	2.66	3.64	8.81	5.75	5
**16**	2.36	2.44	2.49	3.64	2.75	2.54	2.48	5.29	6.20	2
**17**	2.32	3.90	4.46	5.14	3.17	3.73	4.71	6.45	6.01	5
**18**	4.35	3.91	4.43	4.42	4.92	3.82	4.71	8.71	5.49	7

Figure S1. Intra-unit Ha-Hb(cis) and inter-unit Ha-Hb'(trans) ROE interactions of 4 shown on the DFT-optimized X-ray structure.

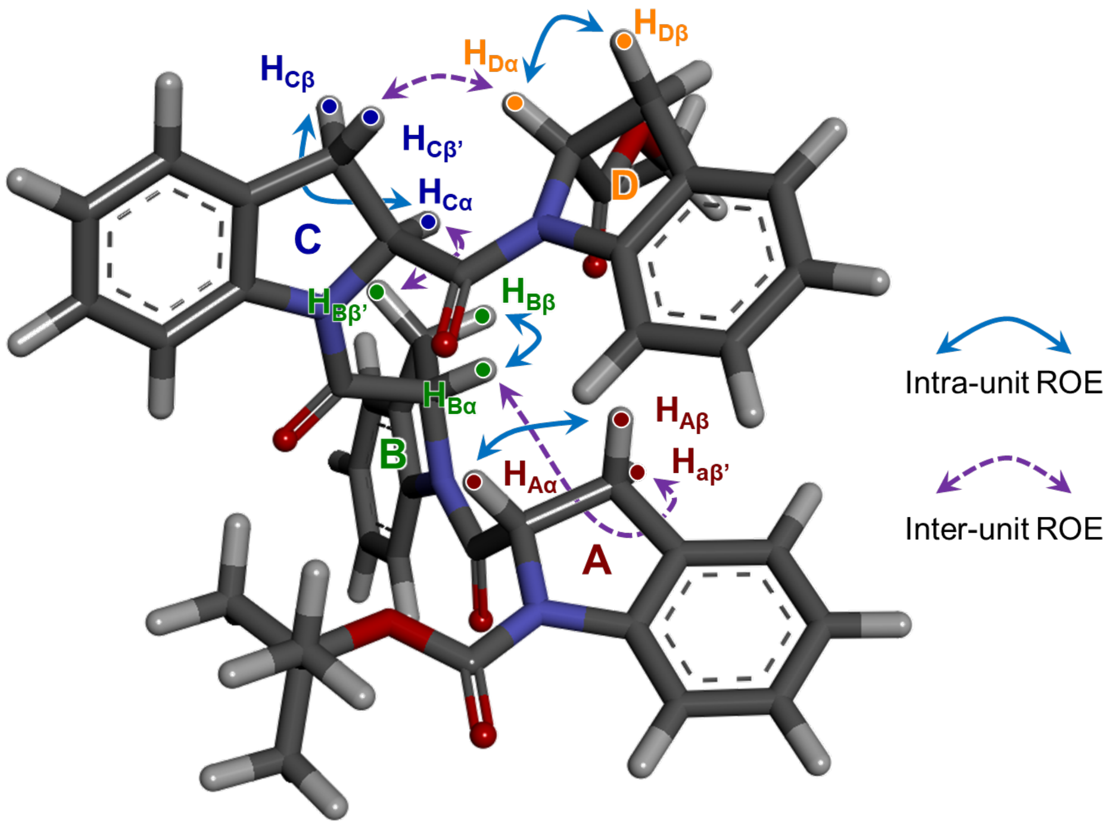

Figure S2. Experimental absorption spectra measured for compounds 1-4 in different solvents.

Cell path length 0.02 cm (MeOH), 0.05 cm (MeCN) and 0.2 cm ($CHCl_3$).

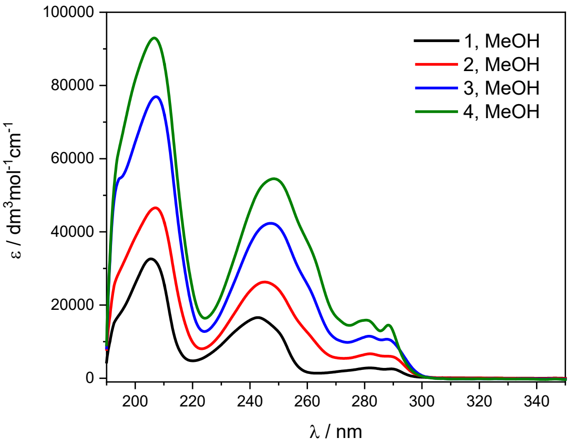

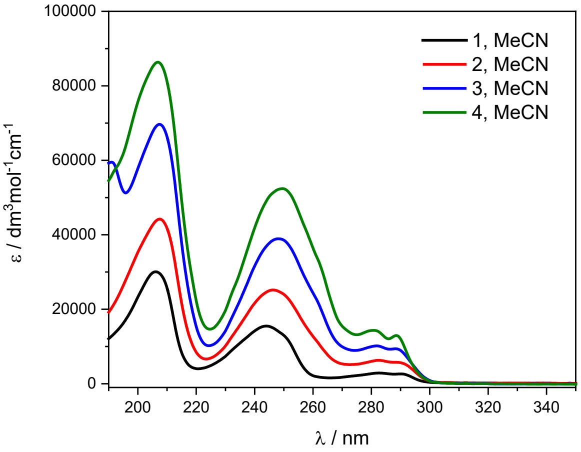

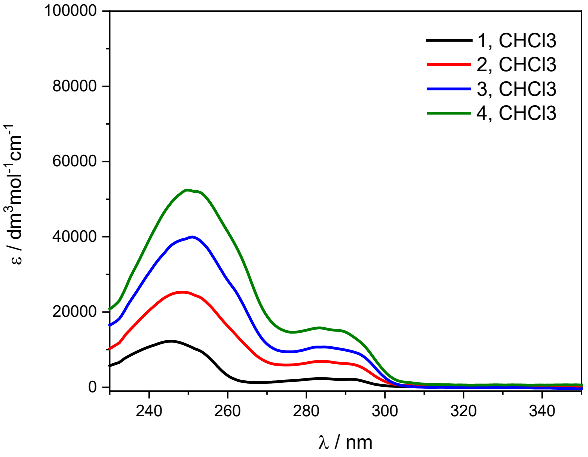

Figure S3. Variable-temperature ECD spectra of **4** measured in MeCN.

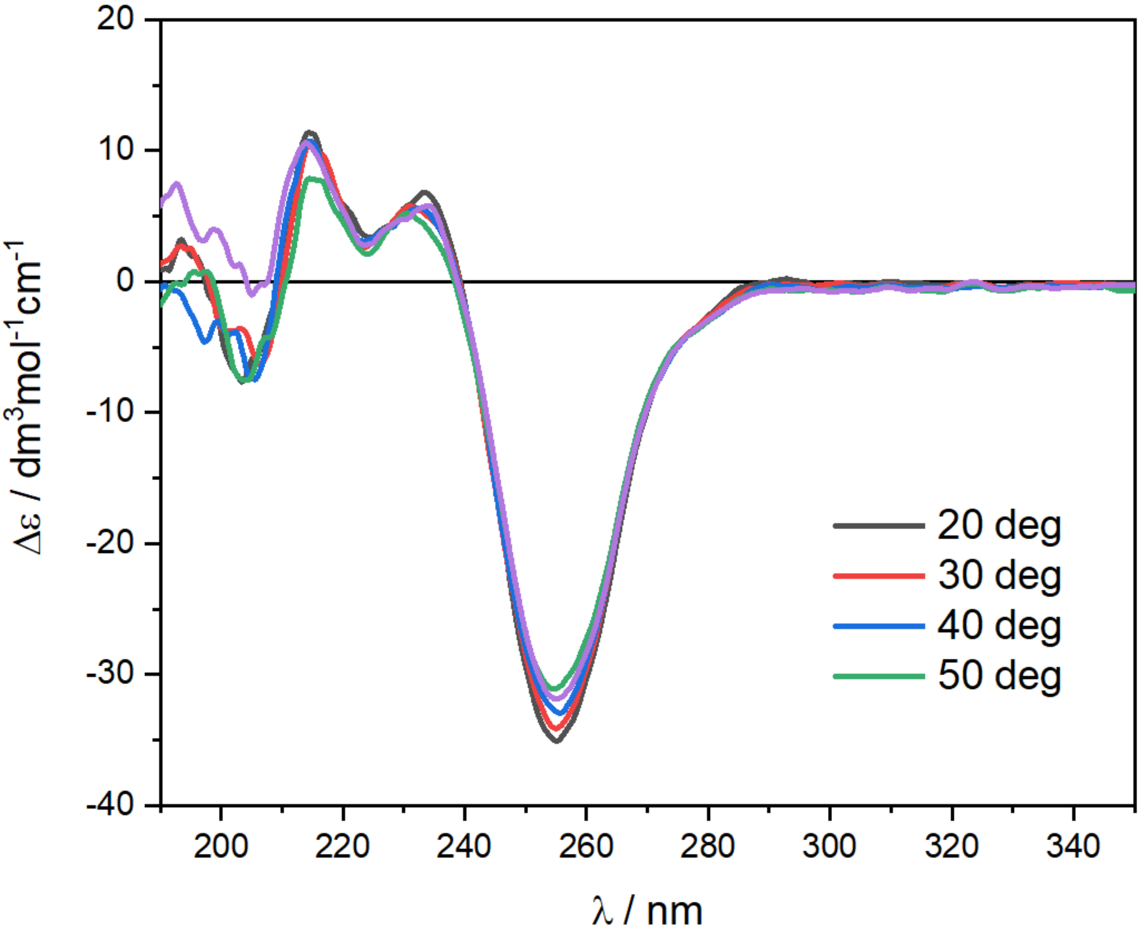

Figure S4. Absorption and ECD spectra of **4** measured in TFE.

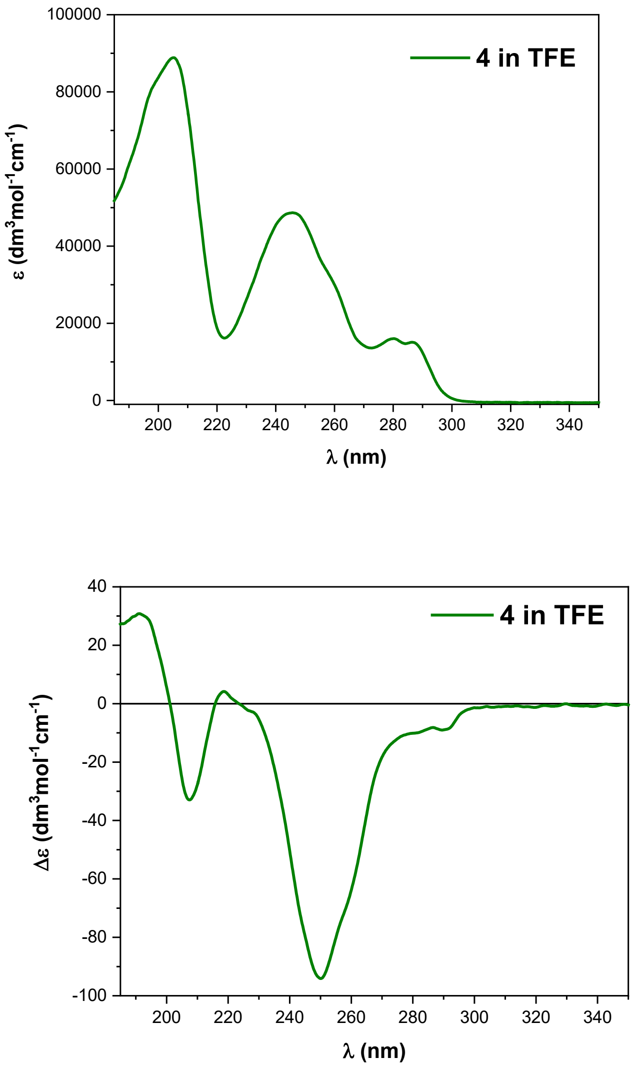

Figure S5. IR (bottom) and VCD (top) spectra of 2 measured in different solvents.

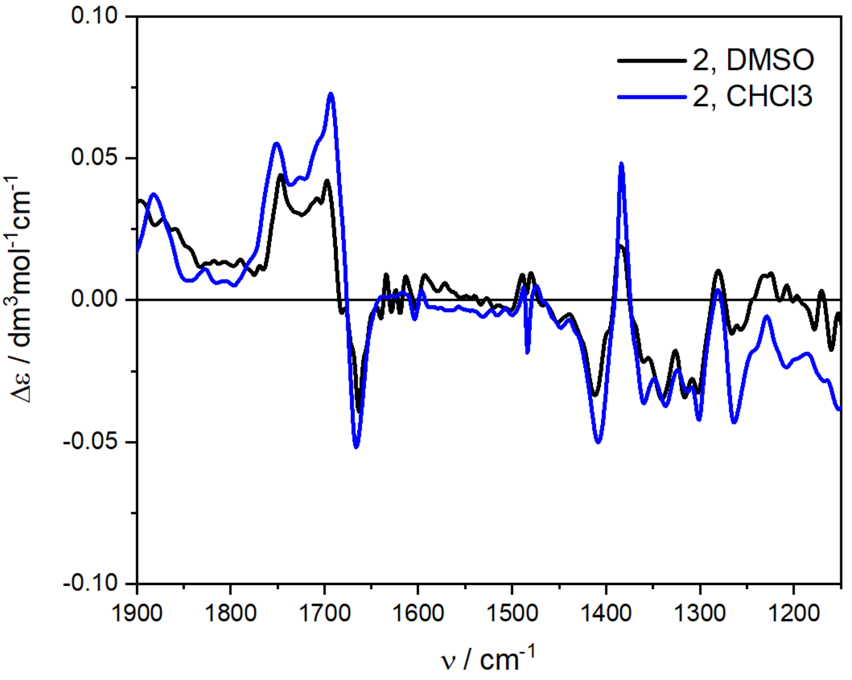

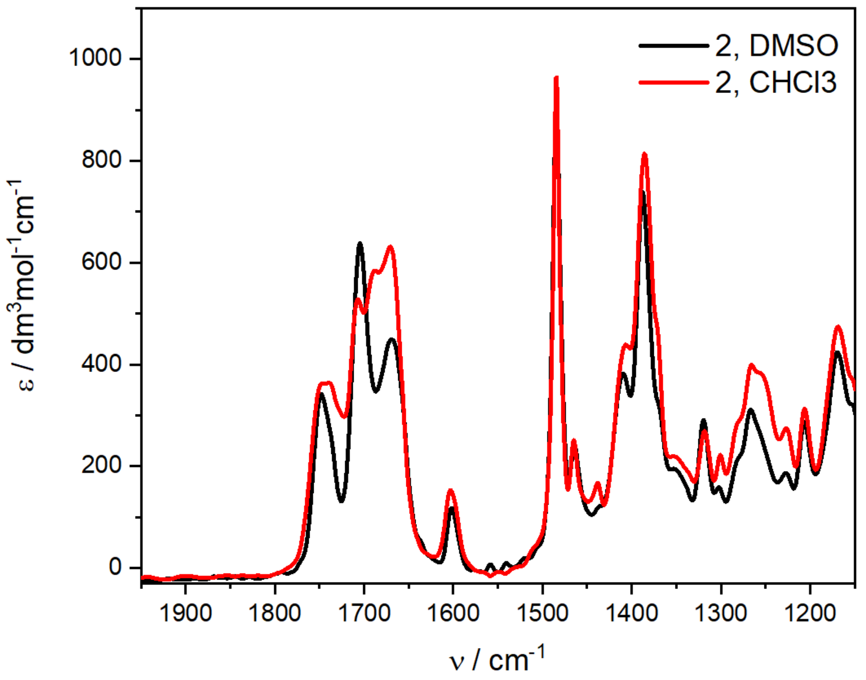

Figure S6. IR (bottom) and VCD (top) spectra of **3** measured in different solvents.

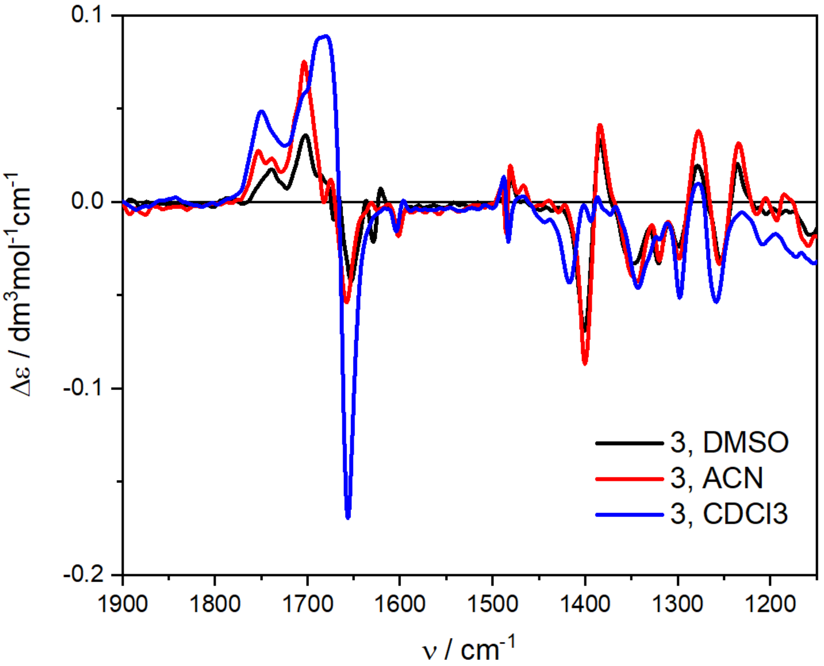

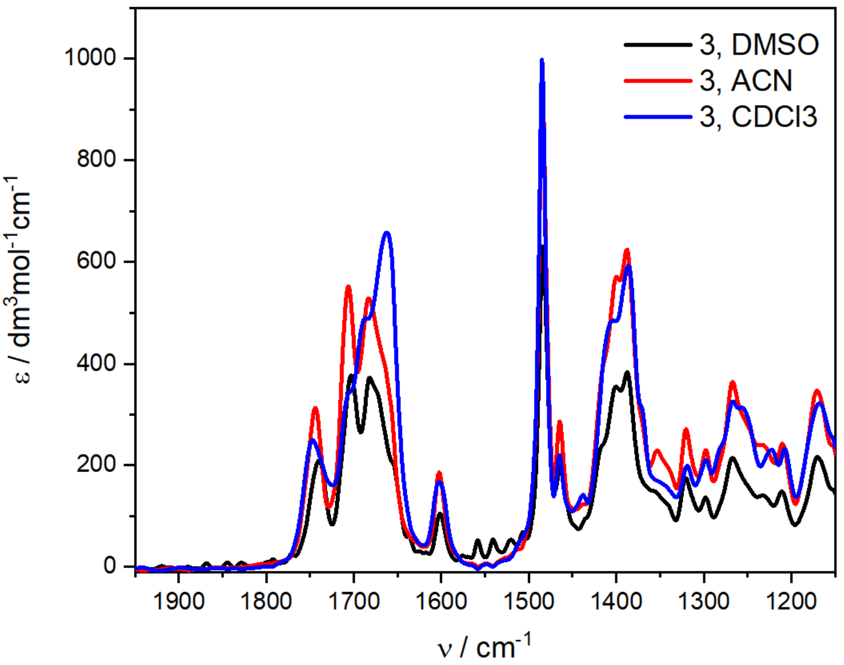

Figure S7. Expansion of the VCD spectrum of (**4**) calculated at B3LYP/6-311+G(d,p)/PCM level using the DFT-optimized X-ray structure.

Plotting parameters: band-width, 8 cm-1, no shift; vertical blue sticks represent the contributing transitions corresponding to the normal modes show below (same order from left to right). Light-blue arrows are the displacement vectors.

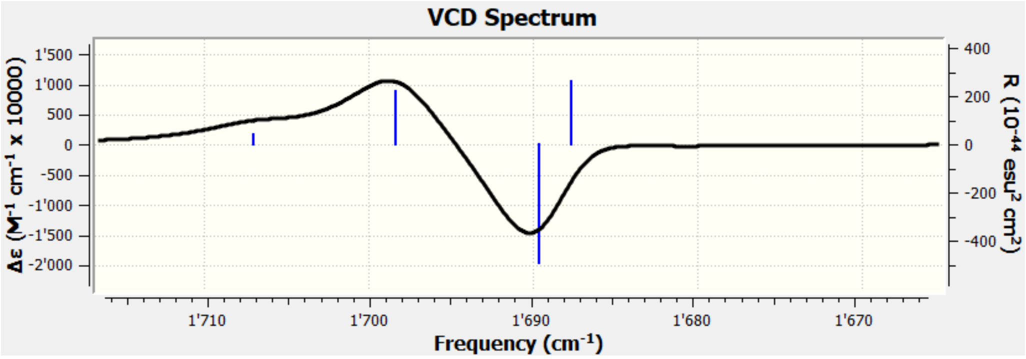

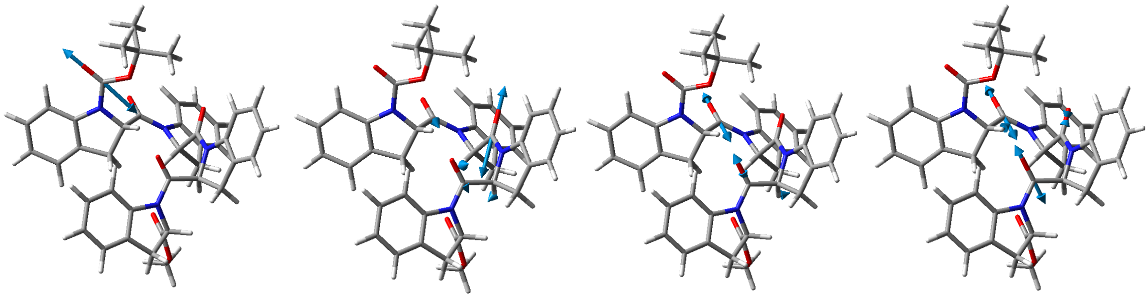

Figure S8. Calculated ECD spectra for **4** at TD-CAM-B3LYP/def2-SVP and at TD-CAM-B3LYP/def2-TZVP level using the structure obtained starting from the X-ray geometry.

Plotting parameters: bandwidth, 0.25 eV; wavelength shift, 20 nm; no scaling.

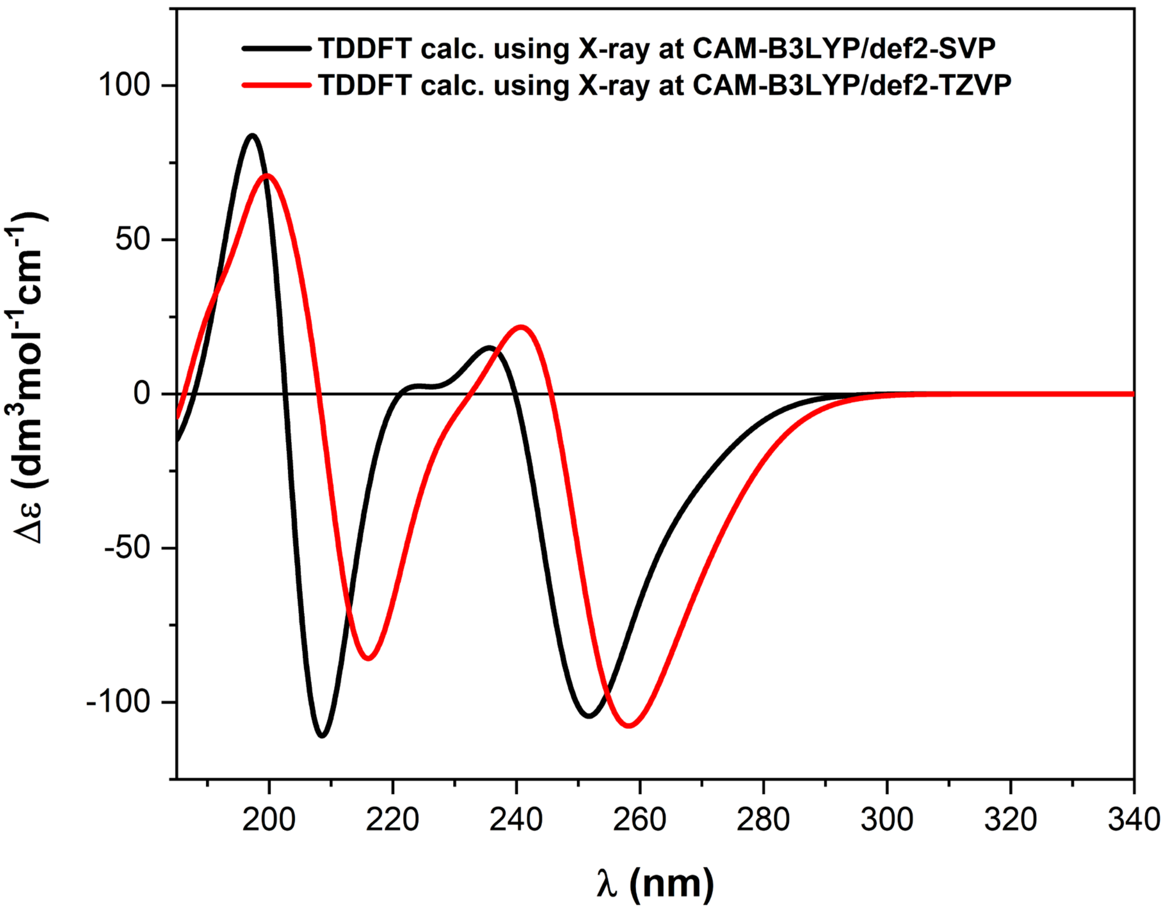

Figure S9. Calculated ECD spectra for 4 at TD-CAM-B3LYP/def2-SVP/PCM for MeOH level using the structures obtained starting from the X-ray geometry and MD simulations.

Plotting parameters: bandwidth, 0.25 eV; wavelength shift, 20 nm; no scaling.

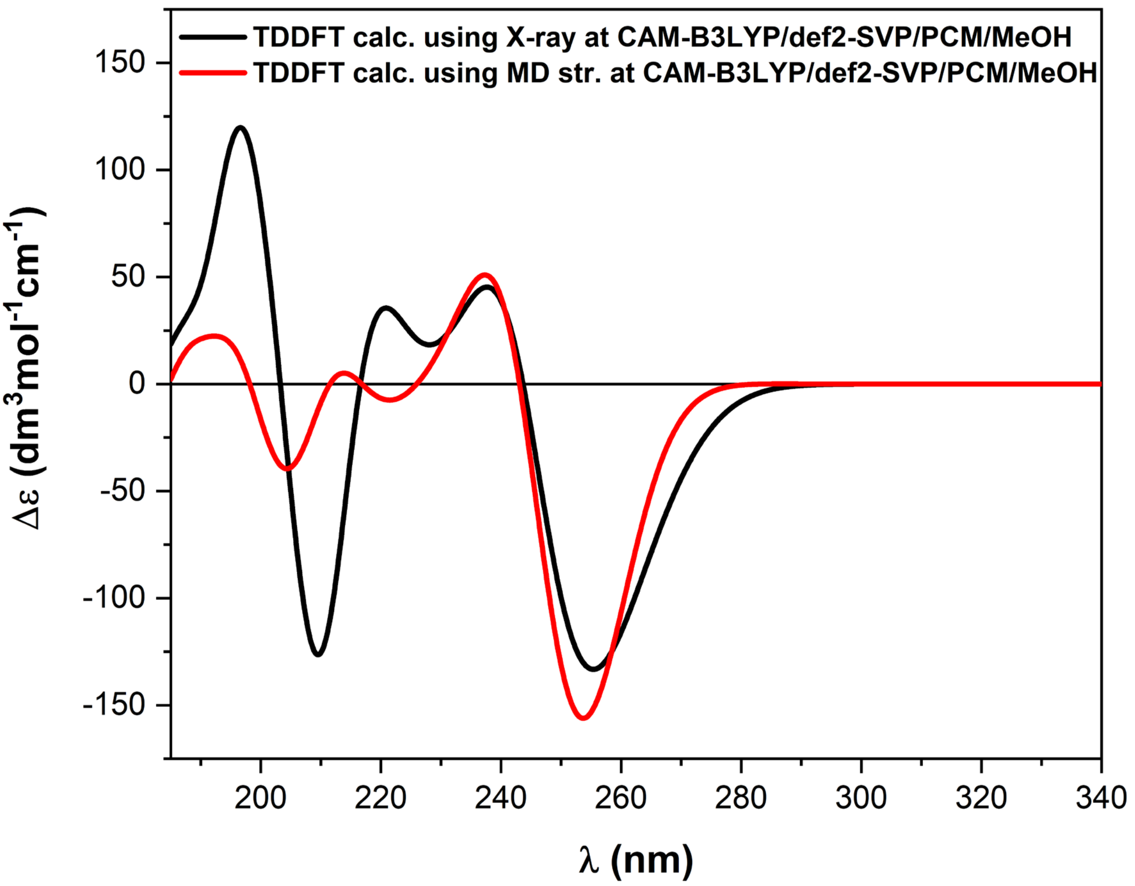

Figure S10. Calculated ECD spectra for **4** at TD-B3LYP/def2-SVP level in vacuum and with PCM solvent model for MeOH using the structures obtained starting from the X-ray geometry and MD simulations.

Plotting parameters: bandwidth, 0.25 eV; wavelength shift, 25 nm; no scaling.

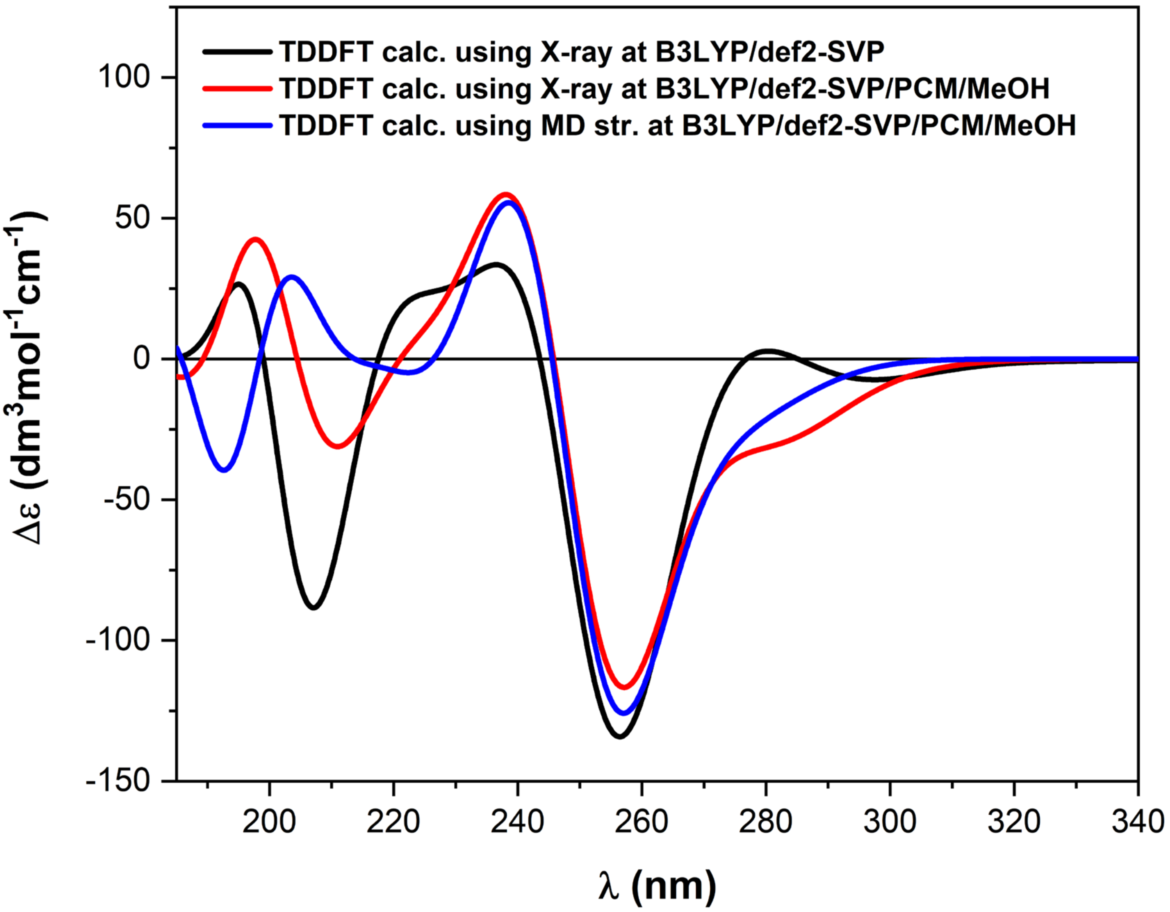

Figure S11. Calculated ECD spectrum (TD-CAM-B3LYP/def2-SVP/PCM for MeCN) using the MD-derived structure with band assignment

Plotting parameters: bandwidth, 0.25 eV; wavelength shift, 20 nm; no scaling.

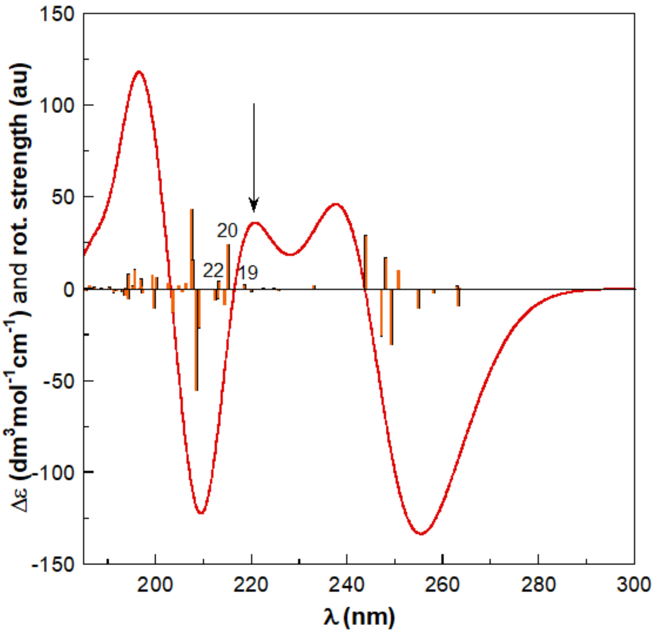

The positive ECD band indicated by the arrow has main contributions from three excited states, for which the major involved single excitations are listed [(occupied MO -> virtual MO (coefficient)], and the MO displayed below.

Excited State 19: 186 ->189 (-0.32), 187 ->189 (0.55)
Excited State 20: 181 ->189 (-0.27), 181 ->192 (0.29), 185 ->198 (-0.22)
Excited State 22: 188 ->190 (0.55)

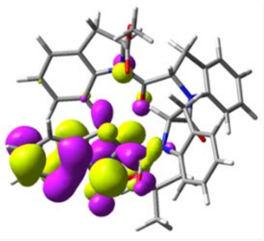
188

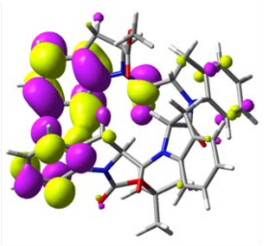
189

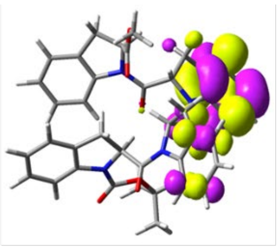
190

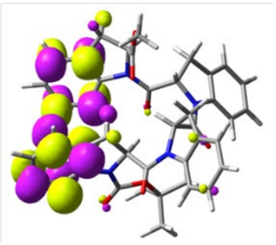
192

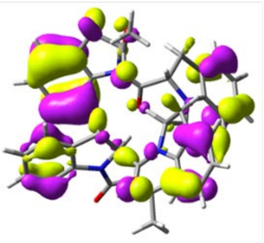
181

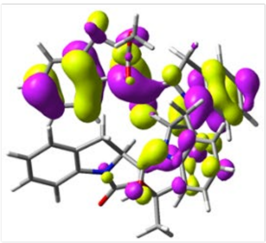
185

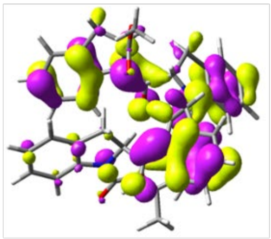
186

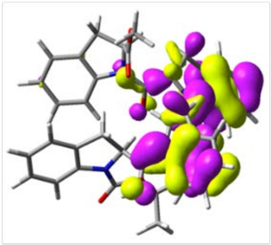
187

Figure S12. View of the molecular structure of (4). Ellipsoids are represented at 20% probability.

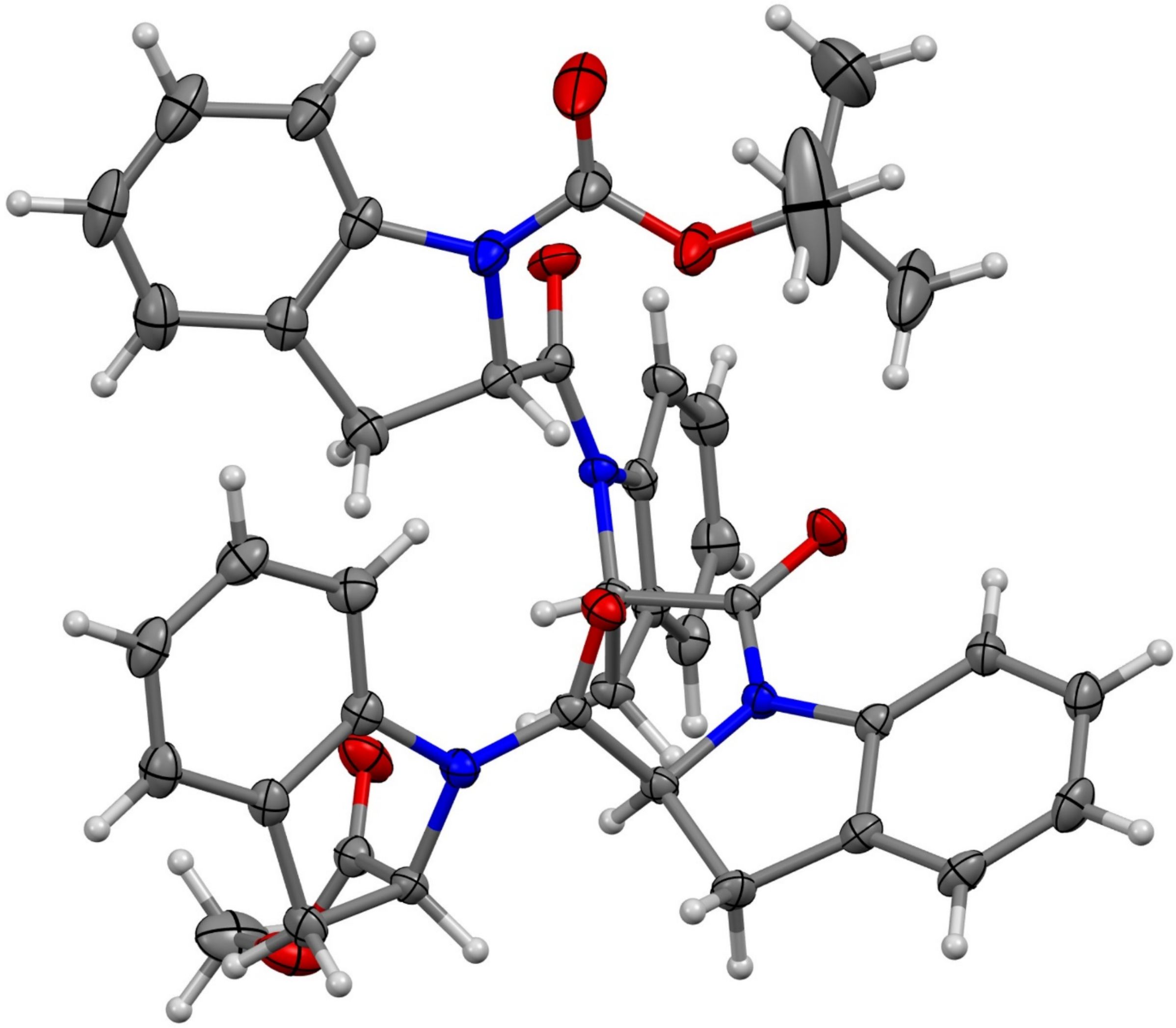

Coordinates of the DFT-optimized geometries

DFT-optimized X-ray structure (**4**)

B3LYP-D3/6-311+G(d,p)/IEFPCM(DMSO)

E=-2370.883667, no imaginary freq

Symbol	X	Y	Z
C	-0.370965	4.483148	3.712077
H	-0.266642	5.496894	4.086986
H	0.520492	3.896067	3.928692
H	-1.249892	4.001053	4.137888
O	-0.532356	4.621826	2.277962
O	-0.662850	2.385398	2.059404
C	-0.676727	3.497688	1.583538
C	-0.861835	3.813001	0.099055
H	-0.047542	4.461995	-0.215654
N	-0.905961	2.579479	-0.709796
C	-2.238280	4.484971	-0.159419
H	-2.631877	4.955418	0.740310
H	-2.134967	5.259778	-0.922961
C	-3.078610	3.340644	-0.662949
C	-4.454195	3.247014	-0.804498
H	-5.092595	4.073058	-0.513449
C	-5.004460	2.069424	-1.321521
H	-6.078697	1.977100	-1.425930
C	-4.175944	1.013226	-1.696036
H	-4.608321	0.098536	-2.082972
C	-2.787927	1.098367	-1.564565
H	-2.151647	0.278404	-1.852756
C	-2.258848	2.271028	-1.032074
C	0.168437	1.813624	-1.046418
O	0.075306	0.821427	-1.758982
C	1.535483	2.240705	-0.482032
H	1.445801	2.568779	0.549222
N	2.469956	1.100414	-0.566598
C	2.187826	3.341517	-1.363765
H	1.451199	3.861658	-1.977419
H	2.684124	4.085432	-0.735929
C	3.183928	2.569507	-2.187856
C	3.916042	2.982013	-3.289253
H	3.791318	3.982733	-3.686689
C	4.816419	2.088297	-3.878497
H	5.394241	2.394351	-4.742216
C	4.968461	0.804549	-3.356460
H	5.665495	0.115652	-3.819132
C	4.233377	0.376890	-2.246581
H	4.344389	-0.618053	-1.849090
C	3.339016	1.278648	-1.674581
C	2.393238	-0.028766	0.200932
O	3.112501	-1.001157	0.022883
C	1.422432	0.014153	1.396303
H	0.524086	0.583852	1.193323
N	1.084510	-1.363281	1.812182
C	2.162744	0.602516	2.628504
H	2.979261	1.263989	2.336535

H	1.463038	1.180284	3.237258
C	2.629306	-0.634115	3.352866
C	3.545875	-0.762600	4.383033
H	4.067407	0.107532	4.765155
C	3.787412	-2.031402	4.922898
H	4.504552	-2.148401	5.726511
C	3.110160	-3.143736	4.425983
H	3.305434	-4.123452	4.846235
C	2.183636	-3.026372	3.383989
H	1.663256	-3.886798	2.995089
C	1.957229	-1.757828	2.859292
C	0.045656	-2.108444	1.326449
O	-0.146974	-3.272518	1.652827
C	-0.940712	-1.348098	0.436652
H	-0.410517	-0.717258	-0.268724
C	-1.926369	-0.533611	1.331007
H	-1.645286	-0.590614	2.384686
H	-1.924240	0.520481	1.060225
C	-3.262230	-1.172798	1.069195
C	-4.496675	-0.867391	1.617434
H	-4.584394	-0.085116	2.362992
C	-5.627242	-1.570474	1.187736
H	-6.599795	-1.339743	1.605728
C	-5.501223	-2.565239	0.219013
H	-6.379704	-3.107100	-0.112116
C	-4.259857	-2.884194	-0.340889
H	-4.162815	-3.650722	-1.093206
C	-3.145773	-2.172820	0.099071
N	-1.802884	-2.267694	-0.321032
C	-1.343398	-3.034739	-1.359510
O	-0.045702	-2.772702	-1.579723
O	-2.033201	-3.824505	-1.983266
C	0.700714	-3.415591	-2.687774
C	2.066489	-2.745796	-2.594429
H	2.507191	-2.895122	-1.608756
H	2.732711	-3.167284	-3.350242
H	1.979885	-1.671869	-2.766219
C	0.804606	-4.915681	-2.425468
H	1.243745	-5.097266	-1.441789
H	-0.172154	-5.394468	-2.474353
H	1.454224	-5.367696	-3.178885
C	0.036826	-3.091678	-4.024859
H	0.692480	-3.422680	-4.833787
H	-0.925550	-3.588634	-4.127734
H	-0.105085	-2.012706	-4.122527

DFT-optimized MD all-cis conformer (**4**)

B3LYP-D3/6-311+G(d,p)/IEFPCM(DMSO)

E=-2370.883667, no imaginary freq

Symbol	X	Y	Z
C	-1.128977	-3.285328	4.403830
C	-3.333840	-1.928323	3.276140
C	-2.419264	-3.493610	4.903839
C	-0.949939	-2.404124	3.348600
C	-2.042373	-1.732894	2.789088

C	-3.502402	-2.818684	4.342321
N	-1.593052	-0.899406	1.725781
C	-0.198990	-1.259872	1.381706
C	0.322017	-1.981497	2.659546
C	-0.182804	-2.236188	0.181881
O	-1.190240	-2.855722	-0.129541
C	-2.335969	0.095536	1.144818
O	-3.510195	0.288730	1.432553
N	1.022374	-2.424088	-0.442349
C	1.262700	-3.369339	-1.479618
C	2.631136	-3.418027	-1.760566
C	3.383541	-2.488472	-0.845237
C	2.252190	-1.641858	-0.188632
C	3.120860	-4.263699	-2.744437
C	2.225465	-5.072956	-3.451317
C	0.862264	-5.022392	-3.162067
C	0.357002	-4.171180	-2.173886
C	2.138156	-0.243265	-0.844015
O	1.228200	0.007222	-1.624460
N	3.118693	0.659072	-0.537395
C	3.257735	1.951512	-1.126470
C	4.473992	2.516070	-0.727468
C	5.234449	1.547371	0.140656
C	4.157450	0.491244	0.501857
C	4.838822	3.780066	-1.166188
C	3.975293	4.480304	-2.014951
C	2.767707	3.907048	-2.411796
C	2.387145	2.633843	-1.975155
C	3.563085	0.702130	1.903642
O	2.384812	0.844795	2.138339
O	4.522787	0.667077	2.825430
C	4.113977	0.821074	4.208421
C	-1.594356	1.032791	0.165134
N	-2.562058	1.883486	-0.552453
C	-2.604830	3.175767	0.023226
C	-1.549617	3.312543	0.932592
C	-0.713876	2.057909	0.943292
C	-1.377310	4.492357	1.639797
C	-2.270631	5.549704	1.430918
C	-3.315644	5.407070	0.518879
C	-3.501388	4.220357	-0.199150
C	-3.376667	1.445167	-1.568484
O	-4.228179	2.139086	-2.099444
O	-3.084889	0.173951	-1.892653
C	-3.813944	-0.539622	-2.978576
C	-3.617276	0.185642	-4.311297
C	-3.121323	-1.900388	-3.004485
C	-5.287815	-0.687691	-2.597986
H	-0.280476	-3.804817	4.835448
H	-4.174792	-1.411285	2.842819
H	-2.575888	-4.181873	5.726245
H	-4.500389	-2.986428	4.731396
H	0.387596	-0.367655	1.183764
H	0.892503	-1.281842	3.276598
H	0.974529	-2.822365	2.419809
H	4.102281	-1.866785	-1.381083
H	3.931351	-3.047588	-0.081458

H	2.414643	-1.543908	0.881684
H	4.183048	-4.297617	-2.960227
H	2.592040	-5.737946	-4.224523
H	0.172170	-5.650307	-3.714234
H	-0.697958	-4.131981	-1.957149
H	6.046824	1.076306	-0.419571
H	5.667044	2.001528	1.030850
H	4.584284	-0.510382	0.481699
H	5.781820	4.216948	-0.857069
H	4.245755	5.470054	-2.363769
H	2.102680	4.455211	-3.069362
H	1.452624	2.194600	-2.284724
H	3.428518	0.020883	4.485158
H	3.635979	1.789845	4.348709
H	5.031958	0.758214	4.785783
H	-1.008414	0.460242	-0.550575
H	-0.476174	1.717832	1.951802
H	0.233200	2.215792	0.420855
H	-0.559021	4.594500	2.344403
H	-2.149726	6.477173	1.978446
H	-4.006420	6.227789	0.360466
H	-4.312904	4.113600	-0.901676
H	-2.553175	0.340485	-4.506984
H	-4.021238	-0.435420	-5.115016
H	-4.125326	1.148063	-4.326133
H	-3.192885	-2.393883	-2.034096
H	-3.593649	-2.535238	-3.757664
H	-2.064915	-1.790952	-3.258377
H	-5.383313	-1.169761	-1.621967
H	-5.792498	0.276894	-2.569535
H	-5.786260	-1.319057	-3.338037

Structures and energies of the optimized clusters

clu_000

B3LYP-D3(BJ)/6-311+G(d,p)/IEFPCM(DMSO)

E= -2371.121968, no imaginary freq

O	0.354227	0.858419	2.517590
C	0.676269	0.048572	3.720222
C	H.092091	0.979713	4.854710
H	0.258476	1.599307	5.182398
H	1.437305	0.381259	5.701255
H	1.913427	1.624879	4.534156
C	-0.512491	-0.840097	4.081003
H	-1.346550	-0.258290	4.468851
H	-0.838976	-1.397053	3.201723
H	-0.196966	-1.552144	4.847498
C	H.846995	-0.796850	3.233130
H	2.203053	-1.431671	4.047428
H	1.533939	-1.427682	2.401503
H	2.669288	-0.163720	2.896567
C	-0.714855	H.659784	2.469142
O	-1.462740	H.931601	3.392083
N	-0.867860	2.142346	H.189336
C	-2.002164	2.823402	0.699232
C	-1.916007	2.939828	-0.691752
C	-0.630267	2.339014	-1.192002
H	0.011542	3.097590	-1.646092
H	-0.808006	1.575125	-1.949211
C	-2.943871	3.522430	-1.414473
H	-2.880176	3.594385	-2.493860
C	-4.070858	3.994282	-0.733986
C	-4.145338	3.879714	0.653998
C	-3.113453	3.294682	H.394626
H	-3.176428	3.195076	2.466452
H	-5.021869	4.244884	1.176263
H	-4.887130	4.444077	-1.285853
C	0.028010	H.748343	0.092752
H	0.072531	0.664731	0.069829
C	H.437491	2.316423	0.272213
O	H.645406	3.318015	0.942289
N	2.411152	H.680828	-0.445246
C	3.723992	2.180191	-0.658927
C	4.277067	H.570740	-1.789196
C	3.276796	0.628176	-2.407994
H	3.704827	-0.335526	-2.684773
H	2.826114	1.064487	-3.303350
C	5.562321	H.887951	-2.195498
H	5.994317	1.415649	-3.070281
C	6.294718	2.826319	-1.458623
C	5.732221	3.428756	-0.334557
C	4.435115	3.114419	0.085853

H	4.000013	3.575919	0.957934
H	C.307653	4.150781	0.232856
H	N.303038	3.081651	-1.761653
C	2.205212	0.483202	-1.291596
H	1.212357	0.493790	-1.718724
C	2.513615	-0.796116	-0.491466
O	3.476256	-0.814307	0.262694
N	H.763184	-1.906619	-0.757400
C	2.053929	-3.219123	-0.293876
C	H.213815	-4.133193	-0.938919
C	0.324603	-3.413413	-1.920585
H	-0.712888	-3.747306	-1.890197
H	0.688888	-3.537516	-2.944153
C	H.297141	-5.485504	-0.653434
H	0.644065	-6.192773	-1.151376
C	2.235590	-5.922638	0.288198
C	3.070022	-5.004305	0.923121
C	2.993190	-3.635595	0.644440
H	3.629593	-2.923643	1.143500
H	3.790365	-5.350024	1.655231
H	2.309816	-6.976815	0.526499
C	0.470125	-1.935551	-1.472539
H	0.487522	-1.259406	-2.317853
C	-0.648084	-1.588866	-0.479186
O	-0.480726	-1.757714	0.723849
N	-1.834063	-1.090966	-0.935816
C	-2.323830	-0.781860	-2.233007
C	-3.527254	-0.076349	-2.113568
C	-3.937171	0.052997	-0.671442
H	-4.913147	-0.403534	-0.495168
H	-3.999003	1.093048	-0.352582
C	-4.187650	0.383788	-3.241077
H	-5.113159	0.938261	-3.140004
C	-3.650171	0.120247	-4.503596
C	-2.474761	-0.618418	-4.617765
C	-1.800318	-1.087685	-3.487389
H	-0.913831	-1.685586	-3.623306
H	-2.072096	-0.846704	-5.597072
H	-4.155479	0.475777	-5.393062
C	-2.813516	-0.691192	0.095672
H	-2.325484	-0.045574	0.820646
C	-3.336492	-1.911643	0.837206
O	-3.436773	-3.020212	0.365951
O	-3.719253	-1.567582	2.068255
C	-4.290611	-2.614308	2.886313
H	-5.182748	-3.018853	2.409097
H	-4.541526	-2.137388	3.829453
H	-3.557749	-3.405967	3.039346

clu_001
B3LYP-D3(BJ)/6-311+G(d,p)/IEFPCM(DMSO)

E= -2371.125946 , no imaginary freq

O	0.463418	2.375947	-1.913068
C	-0.159562	2.633329	-3.234188
C	0.497271	H.748631	-4.292166
H	-0.072574	1.825847	-5.221117
H	1.524808	2.049729	-4.488302
H	0.481656	0.706521	-3.965515
C	-1.602195	2.201220	-3.003868
H	-1.639463	1.145902	-2.734721
H	-2.057008	2.776055	-2.196575
H	-2.181443	2.356260	-3.916758
C	-0.069683	4.120946	-3.561644
H	0.962767	4.430776	-3.714517
H	-0.636536	4.322037	-4.474019
H	-0.504369	4.712434	-2.752360
C	H.787447	2.485575	-1.727960
O	2.582171	2.998972	-2.498618
N	2.134877	H.922618	-0.527616
C	3.450791	H.760083	-0.047307
C	3.441439	0.905875	H.059608
C	2.041727	0.437951	H.351471
H	1.745678	0.637920	2.382992
H	1.936190	-0.633955	1.193335
C	4.625046	0.571039	H.696312
H	4.616313	-0.097799	2.549577
C	5.829976	H.097399	1.217609
C	5.828158	H.949423	0.113905
C	4.639469	2.295786	-0.536661
H	4.638303	2.949190	-1.394619
H	C.763904	2.355157	-0.253110
H	C.763571	0.841986	1.704419
C	H.164597	1.240638	0.341946
H	0.550403	0.596227	-0.276219
C	0.294533	2.242656	H.105625
O	0.654661	3.398846	H.286056
N	-0.842551	H.725216	1.663045
C	-1.631021	2.362717	2.656483
C	-2.451185	H.414138	3.277959
C	-2.182383	0.047519	2.701691
H	-3.090611	-0.519436	2.493527
H	-1.558224	-0.550302	3.370372
C	-3.320771	H.793687	4.286625
H	-3.957276	1.059639	4.767425
C	-3.364160	3.137692	4.676025
C	-2.540082	4.073595	4.053372
C	-1.659548	3.702194	3.031155
H	-1.025968	4.426152	2.544576
H	-2.582153	5.112969	4.357542
H	-4.042234	3.450554	5.460942
C	-1.393670	0.378591	H.405359
H	-0.597037	-0.339580	1.258092

C	-2.383998	0.441128	0.226003
O	-2.961010	H.484971	-0.041778
N	-2.650630	-0.739320	-0.411203
C	-3.586101	-0.909098	-1.464743
C	-3.619944	-2.252622	-1.849024
C	-2.693622	-3.066481	-0.984543
H	-2.052866	-3.730366	-1.566397
H	-3.252340	-3.680420	-0.274240
C	-4.450194	-2.670222	-2.876517
H	-4.471709	-3.712120	-3.174723
C	-5.259068	-1.728133	-3.520415
C	-5.224558	-0.391624	-3.124988
C	-4.388186	0.041519	-2.091823
H	-4.352486	1.076229	-1.794613
H	-5.852019	0.334052	-3.629165
H	-5.911703	-2.038199	-4.327572
C	-1.873747	-1.982505	-0.231732
H	-1.795434	-2.216807	0.825499
C	-0.481622	-1.799049	-0.862072
O	-0.286473	-0.913157	-1.684937
N	0.500237	-2.649361	-0.451053
C	H.873266	-2.538903	-0.812351
C	2.571823	-3.646995	-0.326719
C	H.614978	-4.626251	0.301151
H	1.964837	-5.032861	1.249024
H	1.418334	-5.466265	-0.369262
C	3.947440	-3.725762	-0.476991
H	4.492224	-4.581278	-0.095052
C	4.620504	-2.682889	-1.122174
C	3.910890	-1.589755	-1.616439
C	2.523685	-1.502811	-1.477411
H	1.978152	-0.657590	-1.861434
H	4.437897	-0.780993	-2.107824
H	5.696955	-2.724851	-1.235641
C	0.323198	-3.780575	0.480498
H	-0.560856	-4.362410	0.229381
C	0.192071	-3.301976	H.925604
O	0.356991	-2.162428	2.296905
O	-0.122354	-4.320780	2.719618
C	-0.247797	-4.027317	4.133834
H	-1.035746	-3.291742	4.289931
H	0.698850	-3.650142	4.518390
H	-0.504817	-4.973717	4.600113

clu_002
B3LYP-D3(BJ)/6-311+G(d,p)/IEFPCM(DMSO)

E= -2371.119179, no imaginary freq

O	-3.799416	-1.603324	0.318145
C	-4.527606	-1.601683	H.612705
C	-5.598541	-0.540699	H.377938
H	-5.138884	0.426006	1.164989

H	-6.241188	-0.819911	0.540311
H	-6.217261	-0.440514	2.271808
C	-5.154389	-2.971453	H.854037
H	-5.772465	-3.261856	1.001193
H	-4.391943	-3.730997	2.017267
H	-5.794593	-2.920298	2.738002
C	-3.580656	-1.173977	2.731269
H	-3.090909	-0.232938	2.476070
H	-4.159691	-1.017197	3.644140
H	-2.823480	-1.931573	2.923564
C	-2.699390	-2.335398	0.135205
O	-2.253295	-3.174547	0.900923
N	-2.082457	-2.048696	-1.061308
C	-2.382221	-1.047223	-2.014725
C	-1.331226	-0.960237	-2.937087
C	-0.284664	-1.998920	-2.630613
H	0.729942	-1.598633	-2.640307
H	-0.327543	-2.812706	-3.359768
C	-1.376198	-0.046809	-3.976846
H	-0.552393	0.023291	-4.677921
C	-2.494689	0.782452	-4.107413
C	-3.549512	0.670259	-3.204966
C	-3.512681	-0.244068	-2.147283
H	-4.331469	-0.316177	-1.450886
H	-4.417094	1.311349	-3.310000
H	-2.538536	1.511390	-4.907001
C	-0.694683	-2.508394	-1.223190
H	-0.649307	-3.590144	-1.134861
C	0.168093	-1.851990	-0.140191
O	-0.235836	-0.865704	0.463703
N	H.412010	-2.362939	0.099919
C	H.978506	-3.620907	-0.259403
C	2.868227	-4.026756	0.742446
C	2.925322	-2.992659	H.836564
H	3.945391	-2.753154	2.132357
H	2.382110	-3.329998	2.723466
C	3.551515	-5.225591	0.629277
H	4.238296	-5.538582	1.407290
C	3.353373	-6.020256	-0.505457
C	2.489983	-5.595410	-1.511791
C	H.798470	-4.383610	-1.407944
H	1.170093	-4.064281	-2.223851
H	2.356617	-6.199603	-2.401071
H	3.885338	-6.958189	-0.608804
C	2.206861	-1.775019	H.211773
H	1.519958	-1.307836	1.905846
C	3.233888	-0.783650	0.636391
O	4.216049	-1.218312	0.042182
N	3.052979	0.550945	0.856273
C	4.014704	H.557892	0.561275
C	3.627244	2.754255	H.176477

C	2.352407	2.540619	H.950449
H	1.635751	3.352989	1.835428
H	2.560340	2.420651	3.017797
C	4.400278	3.895356	H.044145
H	4.097143	4.821203	1.519322
C	5.576125	3.833905	0.287840
C	5.956078	2.637541	-0.317862
C	5.181957	H.478720	-0.192783
H	5.472965	0.556357	-0.667645
H	C.865598	2.598604	-0.905915
H	C.189309	4.719276	0.170089
C	H.828414	1.213108	1.346897
H	1.317459	0.589255	2.067050
C	0.908023	H.520476	0.158048
O	H.309444	1.414104	-0.995702
N	-0.339846	2.016316	0.407758
C	-1.111532	2.128217	H.590224
C	-2.416605	2.516657	H.256648
C	-2.565729	2.671884	-0.231105
H	-2.901481	3.675054	-0.501867
H	-3.283728	1.963845	-0.643509
C	-3.376468	2.705215	2.237368
H	-4.381362	3.004474	1.962403
C	-3.037179	2.501875	3.577263
C	-1.740845	2.118097	3.907840
C	-0.763210	H.931135	2.926352
H	0.228120	1.652463	3.240430
H	-1.471142	1.961442	4.945388
H	-3.779189	2.640188	4.353893
C	-1.145222	2.371222	-0.774682
H	-1.152729	1.540323	-1.476430
C	-0.549403	3.589555	-1.464193
O	0.111839	4.443226	-0.918647
O	-0.912782	3.620958	-2.748620
C	-0.489945	4.771663	-3.513565
H	0.598079	4.828911	-3.532316
H	-0.902082	5.682771	-3.080184
H	-0.882118	4.611793	-4.514135

clu_003
B3LYP-D3(BJ)/6-311+G(d,p)/IEFPCM(DMSO)
E= -2371.119916, no imaginary freq

O	-3.551407	0.712708	-0.605382
C	-4.510548	H.361499	-1.529870
C	-5.912340	H.305893	-0.928320
H	-6.289264	0.284849	-0.890594
H	-5.907767	1.723898	0.081071
H	-6.588535	1.904059	-1.543117
C	-3.991989	2.795099	-1.577355
H	-2.973851	2.818922	-1.968469
H	-4.628637	3.395573	-2.230107

H	-3.989904	3.240031	-0.580890
C	-4.435915	0.708551	-2.907293
H	-4.752470	-0.332273	-2.869792
H	-5.091247	1.250494	-3.593255
H	-3.418398	0.761704	-3.299486
C	-3.699576	-0.558081	-0.185064
O	-4.458950	-1.388270	-0.654542
N	-2.845242	-0.785990	0.858242
C	-2.633196	-2.009898	H.523186
C	-1.824765	-1.795747	2.645015
C	-1.476199	-0.332689	2.768636
H	-0.402073	-0.168814	2.870143
H	-1.962842	0.119548	3.636231
C	-1.479985	-2.854526	3.468138
H	-0.851021	-2.691245	4.335916
C	-1.947189	-4.136912	3.160733
C	-2.744288	-4.339738	2.035857
C	-3.101270	-3.279116	H.196693
H	-3.722088	-3.432497	0.327534
H	-3.094604	-5.337543	1.798497
H	-1.679228	-4.975040	3.792474
C	-2.026462	0.285139	H.451598
H	-2.659046	1.145034	1.629808
C	-0.880082	0.628977	0.504366
O	-0.334974	-0.258330	-0.140105
N	-0.456304	H.926882	0.428730
C	-1.133517	3.119684	0.820627
C	-0.919568	4.109158	-0.144517
C	-0.010009	3.585285	-1.226840
H	0.784978	4.285358	-1.480984
H	-0.567965	3.360120	-2.140254
C	-1.500613	5.357610	-0.004196
H	-1.343724	C.122900	-0.755631
C	-2.281932	5.622455	H.126670
C	-2.443013	4.648984	2.109899
C	-1.854553	3.386495	H.977262
H	-1.945213	2.666008	2.777402
H	-3.015762	4.871243	3.002190
H	-2.741008	C.595877	1.250086
C	0.544958	2.276298	-0.614383
H	0.576996	1.467473	-1.332466
C	H.895200	2.536100	0.071972
O	2.007250	3.497379	0.827730
N	2.942849	H.709674	-0.209057
C	4.267576	H.891695	0.275904
C	5.131049	H.024125	-0.401120
C	4.362911	0.231273	-1.424500
H	4.629527	-0.825795	-1.427260
H	4.529747	0.622887	-2.431892
C	6.483718	H.002901	-0.104271
H	N.149755	0.327982	-0.629503

C	6.975112	H.863794	0.883096
C	6.108372	2.726941	H.551337
C	4.740488	2.756255	H.260145
H	4.071443	3.421135	1.780660
H	C.494546	3.388603	2.317899
H	O.029937	1.856530	1.130110
C	2.888015	0.460372	-0.993622
H	2.238556	0.575089	-1.852960
C	2.429086	-0.700496	-0.087994
O	2.457048	-0.599496	H.130253
N	2.096712	-1.856427	-0.733206
C	H.755872	-3.083807	-0.106852
C	H.718203	-4.101184	-1.066131
C	2.081295	-3.544857	-2.419433
H	1.419336	-3.883159	-3.215325
H	3.104229	-3.819016	-2.691688
C	H.398967	-5.397401	-0.699108
H	1.366484	-6.186025	-1.442230
C	H.115238	-5.672106	0.643962
C	H.151816	-4.649907	1.589891
C	H.469699	-3.336509	1.230856
H	1.482090	-2.542976	1.959652
H	0.912553	-4.865469	2.623313
H	0.857527	-6.680212	0.946078
C	H.989113	-2.017761	-2.207507
H	2.790716	-1.489356	-2.713816
C	0.670950	-1.405985	-2.690269
O	0.603336	-0.327374	-3.242560
O	-0.370070	-2.183004	-2.421164
C	-1.675788	-1.636517	-2.713032
H	-2.387681	-2.346489	-2.303592
H	-1.804612	-1.535465	-3.790499
H	-1.774506	-0.669890	-2.227076

clu_004
B3LYP-D3(BJ)/6-311+G(d,p)/IEFPCM(Chloroform)
E= -2371.105182, no imaginary freq

O	4.981413	-0.363934	0.366334
C	5.673459	-1.631061	0.053957
C	5.265051	-2.109112	-1.338048
H	5.651427	-3.118660	-1.496945
H	4.176686	-2.134467	-1.422634
H	5.662487	-1.457715	-2.114668
C	N.181899	-1.446837	0.196367
H	N.676221	-2.415030	0.083829
H	N.566091	-0.765903	-0.561148
H	N.423680	-1.052123	1.186087
C	5.127537	-2.567322	H.129095
H	4.041953	-2.652577	1.044197
H	5.567217	-3.560258	1.014796
H	5.370103	-2.191296	2.125202

C	5.064022	0.711795	-0.440297
O	5.873213	0.880971	-1.331840
N	4.097841	H.616582	-0.076419
C	3.768943	2.799418	-0.771095
C	2.631027	3.375200	-0.194333
C	2.187225	2.560321	0.994169
H	1.116919	2.352020	1.001570
H	2.430948	3.076321	1.927309
C	2.103231	4.547923	-0.707857
H	1.224907	4.998196	-0.259270
C	2.721556	5.147586	-1.810678
C	3.857071	4.567442	-2.373593
C	4.401797	3.384811	-1.863292
H	5.275907	2.929990	-2.302401
H	4.330255	5.035306	-3.229126
H	2.316513	C.062128	-2.226735
C	3.021882	H.256759	0.859317
H	3.453785	0.932906	1.801046
C	2.185180	0.127087	0.253653
O	2.183197	-0.082798	-0.952087
N	H.435129	-0.648760	1.092803
C	H.175240	-0.597292	2.486289
C	0.444664	-1.735370	2.854785
C	0.219495	-2.623178	H.660043
H	-0.813377	-2.961448	1.574741
H	0.858575	-3.509069	1.707399
C	0.042883	-1.928222	4.165407
H	-0.526939	-2.809812	4.435708
C	0.374210	-0.971888	5.129414
C	H.091401	0.162212	4.759054
C	H.494659	0.369755	3.436703
H	2.018364	1.278196	3.192357
H	1.335394	0.912849	5.501320
H	0.063550	-1.106740	C.158162
C	0.630685	-1.714460	0.475102
H	1.241347	-2.216758	-0.269362
C	-0.590601	-1.088855	-0.201011
O	-0.891393	0.082807	-0.008692
N	-1.387325	-1.876876	-0.983258
C	-1.313606	-3.229782	-1.397056
C	-2.434655	-3.536580	-2.179265
C	-3.332405	-2.336939	-2.324190
H	-4.338025	-2.542110	-1.950996
H	-3.423486	-2.024291	-3.366308
C	-2.603312	-4.803439	-2.713091
H	-3.476988	-5.027650	-3.314484
C	-1.637748	-5.783731	-2.470834
C	-0.520731	-5.474600	-1.699680
C	-0.342901	-4.200684	-1.154195
H	0.537128	-4.011472	-0.563579
H	0.231794	-6.230985	-1.511322

H	-1.757056	-6.778511	-2.882042
C	-2.625835	-1.243632	-1.482325
H	-2.340666	-0.393265	-2.093043
C	-3.487294	-0.824238	-0.281620
O	-3.538080	-1.532340	0.714539
N	-4.224284	0.315852	-0.424970
C	-5.090370	0.864524	0.555744
C	-5.790086	H.945667	0.012849
C	-5.425388	2.125230	-1.437875
H	-5.189658	3.159018	-1.693630
H	-6.238488	1.800250	-2.092046
C	-6.684161	2.663438	0.789752
H	-7.224758	3.503909	0.369531
C	-6.877236	2.289075	2.123743
C	-6.176109	H.207646	2.654509
C	-5.269981	0.476397	H.880568
H	-4.723173	-0.357583	2.289529
H	-6.327907	0.925104	3.689699
H	-7.570923	2.842754	2.744975
C	-4.195196	H.194481	-1.616865
H	-4.275403	0.609469	-2.529007
C	-2.900386	H.998908	-1.718004
O	-2.271452	2.104147	-2.747300
O	-2.583261	2.583630	-0.568161
C	-1.322458	3.288825	-0.538400
H	-0.517885	2.597708	-0.782269
H	-1.224466	3.654429	0.480138
H	-1.336637	4.116068	-1.247533

clu_005

B3LYP-D3(BJ)/6-311+G(d,p)/IEFPCM(Chloroform)

E= -2371.115279, no imaginary freq

O	4.631238	-0.447941	-0.614569
C	5.878465	-0.172352	0.134629
C	6.406119	H.074779	-0.567470
H	N.333240	1.400828	-0.091612
H	C.607801	0.866742	-1.619914
H	5.677019	1.885112	-0.506989
C	5.539237	0.127084	H.593100
H	5.107727	-0.741690	2.087901
H	C.451835	0.411616	2.121591
H	4.840739	0.964938	1.654168
C	6.844270	-1.344034	-0.017825
H	N.809483	-1.070570	0.415037
H	C.471555	-2.233081	0.487466
H	C.996887	-1.571800	-1.075262
C	3.806338	-1.457608	-0.289137
O	4.009751	-2.315915	0.547712
N	2.653208	-1.344194	-1.030618
C	H.605603	-2.294299	-1.062040
C	0.730475	-1.993065	-2.110569

C	H.183774	-0.758071	-2.843575
H	0.459180	0.049237	-2.729943
H	1.328267	-0.934016	-3.909795
C	-0.387761	-2.781292	-2.332645
H	-1.072710	-2.544321	-3.138347
C	-0.621831	-3.882101	-1.501296
C	0.254813	-4.166291	-0.455407
C	H.379602	-3.373621	-0.212625
H	2.049914	-3.583076	0.605536
H	0.061651	-5.011752	0.194322
H	-1.488096	-4.511219	-1.668880
C	2.538498	-0.397656	-2.163018
H	3.363029	-0.586976	-2.848541
C	2.649804	H.111988	-1.903176
O	3.075245	H.777879	-2.841395
N	2.242091	H.694352	-0.734459
C	2.216738	3.100311	-0.513873
C	H.860768	3.362485	0.812155
C	H.684400	2.076468	1.569072
H	0.765727	2.045864	2.156443
H	2.520441	1.913075	2.254925
C	H.745363	4.664231	1.271221
H	1.464813	4.858261	2.300230
C	H.993183	5.719648	0.388801
C	2.352698	5.451779	-0.930612
C	2.471171	4.141914	-1.404635
H	2.746841	3.939651	-2.425631
H	2.542444	C.272332	-1.612785
H	1.903716	C.743778	0.730633
C	H.682352	1.004408	0.445867
H	2.303394	0.161365	0.706885
C	0.258353	0.533836	0.164899
O	-0.401792	H.020256	-0.747445
N	-0.278158	-0.434494	0.961379
C	0.230187	-1.189802	2.046949
C	-0.676410	-2.208868	2.362622
C	-1.887370	-2.145159	H.474133
H	-2.808434	-2.038582	2.050360
H	-1.974965	-3.047023	0.866060
C	-0.396597	-3.120713	3.365909
H	-1.103119	-3.910280	3.595259
C	0.802791	-3.012431	4.074662
C	H.689367	-1.981627	3.776947
C	H.412569	-1.054017	2.770190
H	2.117915	-0.260524	2.596105
H	2.613686	-1.884991	4.333516
H	1.036742	-3.721246	4.859446
C	-1.625555	-0.906435	0.582656
H	-1.595499	-1.157171	-0.473448
C	-2.636241	0.207686	0.833376
O	-2.502111	0.982053	H.773163

N	-3.696917	0.317369	-0.019312
C	-4.105973	-0.459685	-1.132990
C	-5.004206	0.285266	-1.907900
C	-5.216762	H.644425	-1.291071
H	-6.268592	1.923754	-1.225911
H	-4.701161	2.422495	-1.859668
C	-5.556929	-0.254080	-3.057529
H	-6.248904	0.327591	-3.655785
C	-5.217332	-1.558057	-3.432132
C	-4.351327	-2.305674	-2.637968
C	-3.796019	-1.773240	-1.471556
H	-3.165447	-2.396258	-0.858622
H	-4.110313	-3.325663	-2.913070
H	-5.642646	-1.992480	-4.328549
C	-4.567985	H.493099	0.109994
H	-3.973091	2.359627	0.387531
C	-5.610885	H.263741	1.198128
O	-5.893096	0.195417	H.683560
O	-6.198296	2.423177	H.519656
C	-7.246624	2.359150	2.511942
H	-8.057016	1.720270	2.160966
H	-7.589903	3.382686	2.635348
H	-6.850010	1.970135	3.449419

clu_006
B3LYP-D3(BJ)/6-311+G(d,p)/IEFPCM(DMSO)
E= -2371.122935, no imaginary freq

O	3.826441	-2.294475	0.302806
C	3.936671	-3.639239	-0.315451
C	3.155184	-4.510638	0.662435
H	3.159001	-5.545097	0.313406
H	3.608552	-4.476154	1.655159
H	2.120358	-4.171765	0.736469
C	3.261083	-3.622986	-1.684468
H	3.267964	-4.634486	-2.096450
H	2.220883	-3.303928	-1.591321
H	3.780117	-2.963445	-2.378401
C	5.401247	-4.062086	-0.381076
H	5.959189	-3.450403	-1.087454
H	5.862870	-3.979256	0.605582
H	5.456658	-5.106310	-0.697555
C	4.320674	-1.193217	-0.285965
O	5.068901	-1.160154	-1.246775
N	3.829117	-0.080905	0.350759
C	4.158775	H.263831	0.061804
C	3.498401	2.109508	0.958276
C	2.689569	H.310601	1.946796
H	1.637161	1.591936	1.928861
H	3.044776	1.450373	2.968609
C	3.636190	3.483643	0.853306
H	3.112355	4.139081	1.538846

C	4.449795	4.011270	-0.153996
C	5.108765	3.157918	-1.038014
C	4.972998	H.769158	-0.947271
H	5.473884	1.108063	-1.636770
H	5.736703	3.573020	-1.817792
H	4.563765	5.084306	-0.249839
C	2.908773	-0.164983	H.500772
H	3.394137	-0.726216	2.296625
C	H.606634	-0.946781	1.289241
O	H.153139	-1.529457	2.269403
N	0.967197	-0.989750	0.075644
C	-0.044071	-1.945996	-0.244725
C	-0.371725	-1.847742	-1.599975
C	0.444144	-0.780772	-2.269951
H	-0.174292	-0.057743	-2.800117
H	1.148020	-1.210502	-2.985389
C	-1.316540	-2.694043	-2.158343
H	-1.566805	-2.613818	-3.209865
C	-1.934132	-3.652209	-1.349599
C	-1.597927	-3.746953	-0.001221
C	-0.651623	-2.895988	0.574279
H	-0.413517	-2.959138	1.621512
H	-2.089578	-4.480010	0.626401
H	-2.675577	-4.319520	-1.772083
C	H.210339	-0.110689	-1.095483
H	2.268055	-0.084471	-1.323568
C	0.851045	H.380896	-0.985125
O	H.389132	2.110213	-1.807407
N	0.022521	H.862908	-0.004901
C	-0.201230	3.254205	0.209257
C	-1.203223	3.427155	H.166709
C	-1.684738	2.094324	H.665564
H	-2.771622	2.010269	1.686690
H	-1.319246	1.908625	2.678563
C	-1.579557	4.696848	H.575887
H	-2.358631	4.823588	2.318718
C	-0.934945	5.806350	H.021775
C	0.076241	5.625298	0.078959
C	0.459687	4.349714	-0.343710
H	1.241218	4.216232	-1.071650
H	0.577899	C.488070	-0.343518
H	-1.219471	C.805996	1.327436
C	-1.052141	H.090694	0.663693
H	-0.643419	0.228352	1.172787
C	-2.084205	0.646349	-0.375053
O	-2.074787	H.113365	-1.510633
N	-3.040982	-0.252758	-0.008979
C	-3.287850	-0.981023	H.185634
C	-4.335046	-1.883177	0.963595
C	-4.836084	-1.787910	-0.450906
H	-5.913094	-1.618346	-0.498668

H -4.620326 -2.700304 -1.009052
C -4.751955 -2.745195 H.963936
H -5.557819 -3.445441 1.776642
C -4.117146 -2.707663 3.208187
C -3.081488 -1.803546 3.427671
C -2.656319 -0.928317 2.425441
H -1.848864 -0.252785 2.646923
H -2.587441 -1.769435 4.391134
H -4.431054 -3.377596 3.999209
C -4.036414 -0.599001 -1.039133
H -3.524232 -0.869122 -1.959222
C -4.938647 0.595460 -1.316628
O -5.163024 H.492769 -0.538308
O -5.494348 0.481776 -2.525837
C -6.446293 H.505352 -2.899045
H -5.960875 2.480742 -2.905597
H -7.282575 1.510547 -2.200463
H -6.781245 1.236793 -3.896916

clu_007
B3LYP-D3(BJ)/6-311+G(d,p)/IEFPCM(DMSO)
E= -2371.120577, no imaginary freq

O 3.752352 -0.170518 0.673075
C 4.190899 0.091403 2.063584
C 3.095491 -0.350638 3.031943
H 3.357657 -0.023658 4.040701
H 2.140927 0.100594 2.755642
H 2.981754 -1.433793 3.037540
C 5.528144 -0.594755 2.328115
H 5.422962 -1.677996 2.324970
H C.259257 -0.304664 1.569779
H 5.904635 -0.279161 3.304222
C 4.345484 H.609018 2.070537
H 5.088932 1.923630 1.335399
H 3.395759 2.090015 1.833001
H 4.667504 1.943793 3.058428
C 3.380374 -1.396781 0.267765
O 3.606932 -2.444688 0.845505
N 2.717334 -1.296705 -0.929594
C 2.121981 -2.365573 -1.628322
C H.367050 -1.864715 -2.694964
C H.482783 -0.363069 -2.756916
H 0.510473 0.130474 -2.807319
H 2.056517 -0.053264 -3.633722
C 0.676172 -2.726577 -3.530439
H 0.079784 -2.336080 -4.346854
C 0.754089 -4.104937 -3.302595
C H.523346 -4.594691 -2.248613
C 2.217692 -3.734601 -1.391449
H 2.799678 -4.112412 -0.565795
H 1.579318 -5.663266 -2.075831

H	0.213319	-4.790030	-3.944068
C	2.245797	-0.000891	-1.449674
H	3.096529	0.647606	-1.635418
C	H.306407	0.638951	-0.426612
O	0.719084	-0.054622	0.394147
N	H.120454	1.992256	-0.471817
C	H.890673	2.989435	-1.140405
C	H.957913	4.134590	-0.339611
C	H.165044	3.928688	0.925253
H	0.510741	4.769873	1.147399
H	1.822205	3.774099	1.785275
C	2.665041	5.245415	-0.768403
H	2.721402	C.131844	-0.147138
C	3.291763	5.213656	-2.019345
C	3.182894	4.084850	-2.828203
C	2.467857	2.959786	-2.404167
H	2.357746	2.116517	-3.068693
H	3.642292	4.075967	-3.809267
H	3.844039	C.077600	-2.368774
C	0.361014	2.639354	0.632830
H	0.355840	1.961717	1.477295
C	-1.059052	2.996378	0.156646
O	-1.218683	3.955812	-0.591549
N	-2.108056	2.270098	0.640899
C	-3.480256	2.588915	0.442175
C	-4.264529	H.763482	1.257223
C	-3.377207	0.856931	2.070650
H	-3.738620	-0.169697	2.123831
H	-3.270992	1.231347	3.093187
C	-5.644460	H.878487	1.253389
H	-6.249834	1.237010	1.883462
C	-6.243205	2.833585	0.424163
C	-5.454320	3.654165	-0.380207
C	-4.059365	3.546062	-0.386292
H	-3.450718	4.175535	-1.014203
H	-5.925210	4.388945	-1.022842
H	-7.321958	2.932276	0.405466
C	-2.030673	0.958951	H.312924
H	-1.170948	0.912980	1.967214
C	-1.942378	-0.142662	0.249503
O	-2.129235	0.108121	-0.936419
N	-1.740226	-1.432211	0.654965
C	-1.224221	-1.965980	H.863469
C	-0.812855	-3.287991	H.648843
C	-1.131937	-3.725970	0.246094
H	-1.908419	-4.495758	0.250572
H	-0.267928	-4.127633	-0.279870
C	-0.220758	-4.021656	2.663887
H	0.103586	-5.039522	2.479645
C	-0.048481	-3.436618	3.921486
C	-0.492813	-2.135921	4.143000

C	-1.091381	-1.388119	3.125110
H	-1.435717	-0.394060	3.355122
H	-0.378043	-1.684414	5.121200
H	0.418822	-3.996071	4.722648
C	-1.627750	-2.424483	-0.431611
H	-0.907585	-2.068071	-1.165381
C	-2.967431	-2.621491	-1.117996
O	-4.046718	-2.513458	-0.582116
O	-2.788970	-2.997546	-2.387528
C	-3.980985	-3.303007	-3.145023
H	-4.620851	-2.422988	-3.206197
H	-4.523571	-4.124197	-2.677013
H	-3.629016	-3.589811	-4.132167

clu_008
B3LYP-D3(BJ)/6-311+G(d,p)/IEFPCM(Chloroform)
E= -2371.118157, no imaginary freq

O	-4.603154	0.415077	0.931328
C	-5.688388	0.973354	0.091917
C	-5.871279	2.367041	0.684122
H	-6.653001	2.898887	0.137883
H	-6.161984	2.302370	1.734503
H	-4.943773	2.938091	0.613681
C	-5.210180	H.059586	-1.356101
H	-5.031667	0.070424	-1.775999
H	-5.974415	1.557522	-1.956847
H	-4.294688	1.651880	-1.415760
C	-6.953968	0.135217	0.248452
H	-6.826925	-0.856977	-0.180260
H	-7.211971	0.036026	1.305330
H	-7.780967	0.637703	-0.259179
C	-4.054691	-0.785450	0.679003
O	-4.505047	-1.645005	-0.057245
N	-2.870092	-0.893063	H.362682
C	-2.035233	-2.034224	H.413096
C	-0.929805	-1.766633	2.227011
C	-1.013697	-0.376472	2.800425
H	-0.133756	0.213296	2.549730
H	-1.097274	-0.395252	3.888161
C	0.051197	-2.728699	2.409555
H	0.912583	-2.510666	3.029443
C	-0.086959	-3.967964	H.775339
C	-1.195069	-4.223626	0.969842
C	-2.187565	-3.260611	0.772700
H	-3.035601	-3.449120	0.134512
H	-1.287653	-5.179557	0.469006
H	0.671168	-4.730580	1.909214
C	-2.311428	0.210416	2.170938
H	-3.026206	0.469842	2.948645
C	-2.075073	H.556998	1.468049
O	-2.184461	2.556298	2.169215

N	-1.756783	H.639516	0.137570
C	-1.575703	2.867647	-0.558860
C	-1.306979	2.608208	-1.906176
C	-1.333809	H.128750	-2.168680
H	-0.476550	0.779899	-2.745603
H	-2.237719	0.846230	-2.715010
C	-1.099102	3.645381	-2.800323
H	-0.887160	3.434535	-3.842293
C	-1.165145	4.963083	-2.338862
C	-1.440420	5.215591	-0.996475
C	-1.649123	4.176647	-0.084572
H	-1.852923	4.376583	0.953464
H	-1.488834	C.238432	-0.641318
H	-0.999845	5.785802	-3.024034
C	-1.346861	0.521998	-0.740345
H	-2.046946	-0.296897	-0.663578
C	0.049408	0.042893	-0.354481
O	0.772114	0.719851	0.370220
N	0.498574	-1.145273	-0.845182
C	-0.090980	-2.154326	-1.646414
C	0.768244	-3.257742	-1.701210
C	2.027816	-2.998163	-0.920317
H	2.914783	-3.077497	-1.552126
H	2.138777	-3.702720	-0.094201
C	0.406225	-4.402807	-2.389232
H	1.075645	-5.255071	-2.416973
C	-0.830217	-4.449667	-3.038715
C	-1.673869	-3.343544	-2.997327
C	-1.315411	-2.182409	-2.308976
H	-2.001884	-1.353405	-2.310581
H	-2.630900	-3.373224	-3.504038
H	-1.128105	-5.340893	-3.577348
C	H.839006	-1.557980	-0.383496
H	1.822545	-1.539535	0.701027
C	2.890752	-0.604907	-0.962096
O	2.793043	-0.213775	-2.117200
N	3.951701	-0.300327	-0.158125
C	5.071497	0.486457	-0.535542
C	6.025981	0.463361	0.485486
C	5.557911	-0.419201	H.613278
H	5.674086	0.042492	2.594562
H	C.105363	-1.365411	1.625699
C	N.209210	H.170905	0.355655
H	N.948068	1.155328	1.148855
C	N.435350	H.906944	-0.812417
C	6.478478	H.921098	-1.825527
C	5.279133	H.212242	-1.705054
H	4.534716	1.226902	-2.484279
H	C.658223	2.496659	-2.726012
H	O.354423	2.468954	-0.927086
C	4.065479	-0.673263	H.271008

H	3.798442	-1.714769	1.424685
C	3.146531	0.166169	2.159677
O	2.451564	-0.313542	3.027873
O	3.255852	H.462365	1.893143
C	2.381453	2.357196	2.618008
H	1.354925	2.184264	2.300438
H	2.702893	3.357497	2.341365
H	2.484699	2.198863	3.690853

clu_009

B3LYP-D3(BJ)/6-311+G(d,p)/IEFPCM(DMSO)

E= -2371.117590, no imaginary freq

O	-0.574441	3.523302	-0.042308
C	-0.596941	4.265353	H.244209
C	-1.867984	5.098800	H.111119
H	-2.740872	4.451787	1.002018
H	-1.999741	5.712525	2.004325
H	-1.807437	5.758387	0.242793
C	-0.713938	3.281514	2.406110
H	-0.901525	3.842624	3.324705
H	-1.547254	2.596721	2.239764
H	0.195546	2.697786	2.530745
C	0.636674	5.157799	H.345206
H	0.546133	5.793283	2.229467
H	1.545639	4.565226	1.431502
H	0.711772	5.803139	0.466769
C	0.354593	2.601795	-0.297955
O	H.330097	2.346962	0.385286
N	0.113543	H.932651	-1.480585
C	-1.055262	H.925546	-2.279804
C	-1.026408	0.818139	-3.138915
C	0.257002	0.052103	-2.956670
H	0.926175	0.216131	-3.805808
H	0.105852	-1.023082	-2.852975
C	-2.075255	0.570352	-4.009345
H	-2.048076	-0.293475	-4.663840
C	-3.169275	H.441560	-4.024229
C	-3.185498	2.547890	-3.176891
C	-2.131017	2.810909	-2.297167
H	-2.161329	3.663063	-1.638931
H	-4.034324	3.221641	-3.189399
H	-4.002337	1.254681	-4.690921
C	0.849599	0.670364	-1.667089
H	0.612881	0.055457	-0.808251
C	2.356558	0.858656	-1.784812
O	2.834964	H.776521	-2.439511
N	3.113030	-0.122603	-1.207413
C	4.518788	-0.260322	-1.334343
C	4.914821	-1.510533	-0.848554
C	3.712344	-2.294715	-0.392881
H	3.848319	-2.754308	0.586576

H	3.467587	-3.088931	-1.102711
C	6.250545	-1.875646	-0.858420
H	C.557010	-2.844086	-0.479758
C	N.197388	-0.976627	-1.362989
C	6.792468	0.265919	-1.847496
C	5.446017	0.646124	-1.841021
H	5.132472	1.608664	-2.211427
H	N.530919	0.958865	-2.233665
H	O.246468	-1.247005	-1.373948
C	2.581020	-1.230561	-0.374576
H	1.682928	-1.631615	-0.826157
C	2.372652	-0.741621	H.070856
O	3.241807	-0.065556	H.603966
N	H.256476	-1.159101	1.738882
C	H.000055	-0.904844	3.115222
C	0.011898	-1.783323	3.570705
C	-0.392078	-2.718423	2.459420
H	-1.467715	-2.883772	2.402350
H	0.095859	-3.691335	2.566764
C	-0.439984	-1.713687	4.878043
H	-1.208695	-2.391552	5.230640
C	0.109120	-0.751408	5.732930
C	H.094215	0.119266	5.268596
C	H.556974	0.057904	3.950112
H	2.313480	0.734968	3.588267
H	1.508693	0.865960	5.935747
H	-0.236616	-0.680048	C.757196
C	0.149114	-1.977821	H.205958
H	0.510369	-2.661678	0.447790
C	-0.955592	-1.059675	0.663571
O	-0.988058	0.128498	0.967942
N	-1.938241	-1.584027	-0.123145
C	-2.204482	-2.889896	-0.613901
C	-3.346831	-2.851000	-1.423650
C	-3.907022	-1.456290	-1.499049
H	-4.955379	-1.422445	-1.196643
H	-3.841782	-1.047846	-2.508124
C	-3.819761	-4.001201	-2.034554
H	-4.704976	-3.959923	-2.658682
C	-3.146546	-5.209230	-1.834628
C	-2.017998	-5.245678	-1.019694
C	-1.534368	-4.092388	-0.395874
H	-0.670479	-4.178331	0.241571
H	-1.500830	-6.182865	-0.853152
H	-3.505833	-6.115834	-2.305772
C	-3.001905	-0.645266	-0.539292
H	-2.557183	0.211611	-1.036331
C	-3.760635	-0.148289	0.682993
O	-3.954215	-0.794405	H.687064
O	-4.227598	H.083742	0.469915
C	-5.008630	H.672771	1.534245

H	-5.287692	2.659073	1.174239
H	-4.404415	1.750900	2.437705
H	-5.894355	1.068755	1.729589

clu_010
B3LYP-D3(BJ)/6-311+G(d,p)/IEFPCM(Chloroform)
E= -2371.112403, no imaginary freq

O	-4.499704	-0.621487	-0.435124
C	-5.514505	-0.571443	-1.518240
C	-4.937859	0.156050	-2.730296
H	-4.785659	1.213615	-2.523296
H	-3.986688	-0.292106	-3.022309
H	-5.632845	0.058805	-3.567595
C	-5.735214	-2.050390	-1.820418
H	-6.464385	-2.155620	-2.626199
H	-4.801249	-2.522390	-2.128810
H	-6.115894	-2.569292	-0.938205
C	-6.794222	0.076027	-0.996120
H	-7.131481	-0.430316	-0.088685
H	-6.646395	1.132719	-0.781564
H	-7.576606	-0.022514	-1.752455
C	-4.023649	0.491500	0.124082
O	-4.331879	H.635257	-0.163446
N	-3.070410	0.225855	H.084915
C	-2.770337	-1.004211	H.729785
C	-2.045525	-0.750645	2.900817
C	-1.792652	0.724496	3.060295
H	-0.735673	0.955647	2.918427
H	-2.092136	1.102386	4.037711
C	-1.660710	-1.794886	3.727119
H	-1.105879	-1.593916	4.636636
C	-2.003081	-3.104900	3.377338
C	-2.705702	-3.347540	2.198549
C	-3.091928	-2.305322	H.352890
H	-3.620411	-2.505682	0.436539
H	-2.951658	-4.364881	1.917989
H	-1.715277	-3.929882	4.017944
C	-2.658488	H.362287	1.938976
H	-3.560171	1.799798	2.368241
C	-1.961795	2.572282	H.308194
O	-2.127366	3.638612	H.891795
N	-1.151227	2.471228	0.211081
C	-0.431144	3.568063	-0.340418
C	0.194284	3.172213	-1.525816
C	-0.142469	H.744012	-1.841129
H	0.737554	1.152971	-2.095585
H	-0.846500	1.687983	-2.675977
C	0.982155	4.056956	-2.242707
H	1.472567	3.735562	-3.154213
C	H.145396	5.360387	-1.766029
C	0.515029	5.752064	-0.586337

C	-0.282437	4.866960	0.145248
H	-0.763402	5.172625	1.058569
H	0.645056	C.762889	-0.217642
H	1.763730	C.063344	-2.311245
C	-0.812648	H.239906	-0.532663
H	-1.706120	0.669663	-0.744152
C	0.177442	0.386341	0.258855
O	0.815327	0.859379	H.192651
N	0.364184	-0.903185	-0.155618
C	-0.474311	-1.738296	-0.957697
C	-0.407004	-3.049552	-0.472651
C	0.524288	-3.114667	0.707295
H	1.194928	-3.970919	0.666897
H	-0.043219	-3.154386	1.639655
C	-1.138055	-4.057332	-1.074316
H	-1.089603	-5.070143	-0.691678
C	-1.930744	-3.754762	-2.186290
C	-1.964327	-2.456332	-2.684757
C	-1.229487	-1.430319	-2.081750
H	-1.243763	-0.444743	-2.518485
H	-2.552629	-2.231689	-3.566007
H	-2.503018	-4.535816	-2.672199
C	H.289193	-1.778556	0.614893
H	1.456378	-1.325069	1.584469
C	2.593430	-1.978087	-0.184210
O	2.612079	-2.777501	-1.111771
N	3.712749	-1.300814	0.210486
C	5.013122	-1.499902	-0.335776
C	5.950740	-0.822357	0.453590
C	5.250705	-0.153472	H.608498
H	5.603869	0.854412	1.818056
H	5.354359	-0.746109	2.522507
C	N.297703	-0.862962	0.134137
H	O.022592	-0.337497	0.745413
C	N.706906	-1.590324	-0.988541
C	6.766331	-2.262492	-1.766904
C	5.403299	-2.229164	-1.455537
H	4.675427	-2.743809	-2.060610
H	N.090282	-2.819598	-2.638220
H	O.756343	-1.627102	-1.255281
C	3.778521	-0.157016	H.137344
H	3.081229	-0.257372	1.962951
C	3.430356	H.133583	0.385416
O	3.103269	H.189768	-0.775702
O	3.567905	2.183554	H.194715
C	3.210556	3.472553	0.644680
H	3.794339	3.672878	-0.252647
H	3.441068	4.192600	1.425046
H	2.149850	3.486219	0.408624

clu_011

B3LYP-D3(BJ)/6-311+G(d,p)/IEFPCM(Chloroform)

E= -2371.106804, no imaginary freq

O	3.862856	0.324170	H.410232
C	4.130897	H.377068	2.422629
C	2.932330	H.541371	3.353944
H	2.650823	0.583729	3.794426
H	3.207092	2.222861	4.162793
H	2.074221	1.949052	2.825105
C	5.328077	0.802711	3.173827
H	5.634367	1.494084	3.961294
H	5.070877	-0.153805	3.633979
H	C.170741	0.650868	2.496490
C	4.501882	2.677316	H.714001
H	4.832707	3.407248	2.456425
H	5.322489	2.506784	1.013321
H	3.651728	3.091512	1.174289
C	2.901451	0.471898	0.493649
O	2.052609	H.350415	0.483121
N	2.961107	-0.490391	-0.477917
C	3.909867	-1.516286	-0.701822
C	3.730408	-2.028552	-1.992457
C	2.627922	-1.279621	-2.700281
H	1.926689	-1.944465	-3.207231
H	3.032172	-0.589902	-3.446038
C	4.526037	-3.063387	-2.454195
H	4.383549	-3.460191	-3.453004
C	5.513001	-3.590390	-1.614121
C	5.683834	-3.073759	-0.331361
C	4.884489	-2.030838	0.146945
H	5.017198	-1.640828	1.142915
H	C.446009	-3.488980	0.317778
H	C.140499	-4.402843	-1.959936
C	H.951417	-0.489300	-1.544505
H	1.727394	0.536976	-1.816307
C	0.703067	-1.257179	-1.076857
O	0.802832	-2.131107	-0.230765
N	-0.482925	-0.955741	-1.693640
C	-1.620262	-1.821511	-1.683911
C	-2.491043	-1.470304	-2.715729
C	-1.985892	-0.265023	-3.454570
H	-2.647208	0.586683	-3.293353
H	-1.895534	-0.429036	-4.528567
C	-3.658130	-2.189908	-2.921507
H	-4.335069	-1.915471	-3.722564
C	-3.950284	-3.266532	-2.079667
C	-3.081473	-3.595758	-1.041451
C	-1.903162	-2.877681	-0.823233
H	-1.247544	-3.120823	-0.005612
H	-3.322667	-4.416599	-0.376651
H	-4.860753	-3.835436	-2.226056
C	-0.585354	-0.001742	-2.822470

H	0.183714	-0.240676	-3.555663
C	-0.412751	H.507825	-2.605187
O	-0.107276	2.123014	-3.624520
N	-0.661718	2.147552	-1.424639
C	-0.677134	3.570229	-1.318112
C	-0.780548	3.945540	0.021020
C	-0.811334	2.737898	0.906316
H	-1.673006	2.757620	1.576084
H	0.095380	2.667342	1.509809
C	-0.839816	5.281737	0.384521
H	-0.921417	5.560149	1.429179
C	-0.794787	6.259389	-0.611830
C	-0.695818	5.880140	-1.949244
C	-0.634525	4.535822	-2.325072
H	-0.553092	4.253447	-3.360313
H	-0.664694	C.638964	-2.722427
H	-0.839584	N.308575	-0.345450
C	-0.875787	H.538633	-0.085958
H	-0.061141	0.860687	0.134492
C	-2.222627	0.823246	0.003641
O	-3.084073	0.974931	-0.848143
N	-2.408427	0.027658	H.107630
C	-3.673886	-0.529874	H.454651
C	-3.782496	-0.621347	2.843730
C	-2.541076	-0.056211	3.488322
H	-2.172122	-0.656187	4.320297
H	-2.717467	0.959225	3.855845
C	-4.922453	-1.157962	3.419474
H	-5.012686	-1.234565	4.497002
C	-5.953309	-1.605525	2.585355
C	-5.828863	-1.511170	H.199731
C	-4.682656	-0.970795	0.609758
H	-4.577372	-0.910525	-0.461158
H	-6.628005	-1.871349	0.562490
H	-6.848877	-2.034255	3.019195
C	-1.534849	-0.024842	2.306285
H	-0.876449	0.831878	2.355351
C	-0.684188	-1.296171	2.354676
O	-1.121401	-2.418042	2.275109
O	0.594735	-0.988933	2.593503
C	H.505542	-2.101294	2.766333
H	1.204852	-2.692642	3.631443
H	2.480068	-1.648487	2.925715
H	1.509273	-2.706903	1.864672

clu_012

B3LYP-D3(BJ)/6-311+G(d,p)/IEFPCM(Chloroform)

E= -2371.113844, no imaginary freq

O	-4.309015	-1.379758	0.038530
C	-5.582213	-1.491299	-0.716991
C	-5.424447	-2.839911	-1.412637

H	-5.320416	-3.640828	-0.677728
H	-6.305544	-3.042829	-2.024648
H	-4.543744	-2.838042	-2.057318
C	-6.756300	-1.510286	0.257798
H	-6.874167	-0.549800	0.756300
H	-7.673038	-1.734176	-0.292873
H	-6.611223	-2.289131	1.010008
C	-5.682336	-0.358828	-1.735951
H	-6.557041	-0.526933	-2.368557
H	-5.784788	0.607848	-1.246936
H	-4.798542	-0.345390	-2.375817
C	-4.034935	-0.315491	0.795556
O	-4.740273	0.666997	0.947958
N	-2.796331	-0.409842	H.391194
C	-1.933688	-1.531353	H.503164
C	-0.977741	-1.284052	2.495009
C	-1.167967	0.084202	3.092218
H	-0.312386	0.723995	2.872652
H	-1.297959	0.053662	4.174288
C	-0.011214	-2.233053	2.789489
H	0.733751	-2.034712	3.551172
C	-0.006546	-3.443371	2.089205
C	-0.955349	-3.674369	H.095187
C	-1.926652	-2.721322	0.779340
H	-2.644218	-2.902794	-0.002661
H	-0.941672	-4.606895	0.543170
H	0.737309	-4.197968	2.315397
C	-2.457606	0.615523	2.400859
H	-3.277914	0.667434	3.115606
C	-2.284825	2.079669	H.970453
O	-2.496948	2.911449	2.846531
N	-1.846720	2.441791	0.725876
C	-1.508999	3.775882	0.360261
C	-1.183808	3.823170	-0.998327
C	-1.369127	2.469981	-1.624609
H	-0.532372	2.169659	-2.256659
H	-2.274719	2.448540	-2.237438
C	-0.788481	5.010348	-1.592151
H	-0.533666	5.036447	-2.645532
C	-0.722056	6.169436	-0.813560
C	-1.054660	6.117404	0.538662
C	-1.453572	4.924202	H.148340
H	-1.709187	4.888987	2.193721
H	-1.002229	N.017801	1.139698
H	-0.411641	N.105825	-1.261412
C	-1.525182	H.534697	-0.395024
H	-2.327488	0.826578	-0.538469
C	-0.215169	0.800632	-0.121885
O	0.582374	H.216654	0.711590
N	0.067197	-0.326717	-0.836921
C	-0.635112	-1.045927	-1.832949

C	0.030765	-2.252019	-2.085449
C	H.271094	-2.366450	-1.243196
H	2.163702	-2.511029	-1.854722
H	1.200057	-3.206517	-0.550438
C	-0.469863	-3.159497	-3.003165
H	0.051356	-4.092481	-3.184791
C	-1.646465	-2.856933	-3.693812
C	-2.289823	-1.644913	-3.461358
C	-1.794124	-0.724072	-2.535294
H	-2.314480	0.209371	-2.406341
H	-3.189447	-1.398231	-4.012182
H	-2.048339	-3.556168	-4.416709
C	H.317682	-1.023770	-0.473301
H	1.312092	-1.162571	0.603431
C	2.505191	-0.161767	-0.891974
O	2.456535	0.528803	-1.902776
N	3.623375	-0.179793	-0.109270
C	3.953568	-0.910956	H.060103
C	5.026726	-0.284073	H.706259
C	5.447586	0.943372	0.938750
H	C.526194	1.007524	0.794011
H	5.125694	1.856673	1.445467
C	5.545892	-0.808932	2.877994
H	C.374148	-0.318513	3.376530
C	4.993857	-1.980996	3.404789
C	3.950148	-2.618153	2.738236
C	3.425827	-2.101098	H.550781
H	2.647809	-2.643899	1.039327
H	3.541888	-3.541222	3.132369
H	5.390543	-2.402935	4.320097
C	4.688932	0.787224	-0.405339
H	4.249468	1.722068	-0.744411
C	5.593782	0.258866	-1.512854
O	5.630642	-0.882609	-1.903106
O	C.372531	H.245012	-1.975231
C	N.320334	0.886375	-3.005138
H	O.011167	0.130110	-2.632469
H	N.848548	1.805474	-3.243755
H	C.794326	0.506048	-3.880441

clu_013
B3LYP-D3(BJ)/6-311+G(d,p)/IEFPCM(DMSO)
E= -2371.122296, no imaginary freq

O	-4.834620	-0.535189	0.403319
C	-5.833265	-0.779369	H.477531
C	-6.523014	0.576883	H.585705
H	-6.964187	0.861553	0.628339
H	-7.317016	0.525718	2.333152
H	-5.810449	1.346991	1.888130
C	-6.810205	-1.858970	H.022129
H	-7.608910	-1.953139	1.761736

H	-7.260666	-1.585714	0.065074
H	-6.312920	-2.822318	0.921110
C	-5.121532	-1.131972	2.781205
H	-4.332615	-0.406372	2.984888
H	-5.847995	-1.103397	3.596673
H	-4.683120	-2.127467	2.739969
C	-3.931337	-1.447523	0.065680
O	-3.871599	-2.599199	0.472090
N	-2.999736	-0.957391	-0.825089
C	-3.000585	0.297888	-1.496038
C	-2.260383	0.198938	-2.676069
C	-1.761204	-1.206116	-2.871507
H	-0.681730	-1.249912	-3.022248
H	-2.219104	-1.679689	-3.741988
C	-2.072290	H.311888	-3.481963
H	-1.494082	1.233405	-4.395742
C	-2.631856	2.533322	-3.096551
C	-3.367981	2.620331	-1.916846
C	-3.561319	H.506798	-1.097538
H	-4.107723	1.587614	-0.173335
H	-3.779579	3.573553	-1.608646
H	-2.483010	3.414025	-3.709302
C	-2.192132	-1.945126	-1.564282
H	-2.822325	-2.801085	-1.797567
C	-0.945332	-2.568277	-0.924058
O	-0.472408	-3.516752	-1.541066
N	-0.301319	-2.027081	0.165071
C	0.969066	-2.526542	0.598192
C	H.166092	-2.241279	1.948401
C	-0.032338	-1.548700	2.525344
H	0.239505	-0.613033	3.014133
H	-0.545385	-2.173291	3.259237
C	2.356371	-2.583788	2.573718
H	2.507753	-2.356320	3.622495
C	3.353207	-3.221653	H.832007
C	3.150624	-3.495762	0.479798
C	H.958219	-3.151634	-0.158151
H	1.814849	-3.358494	-1.205206
H	3.933547	-3.972903	-0.096833
H	4.287314	-3.497060	2.306799
C	-0.954160	-1.296013	H.291899
H	-1.922010	-1.749975	1.468732
C	-1.258779	0.205072	H.200795
O	-2.172435	0.593590	H.924539
N	-0.534999	H.063473	0.431146
C	-0.768369	2.466773	0.357944
C	0.026169	3.014748	-0.651514
C	0.824060	H.934728	-1.327353
H	1.868580	2.203590	-1.484105
H	0.386174	1.689176	-2.297679
C	-0.033049	4.367912	-0.939777

H	0.579596	4.786684	-1.729851
C	-0.897946	5.181121	-0.201319
C	-1.682682	4.627134	0.808782
C	-1.631158	3.262491	H.106222
H	-2.247417	2.834135	1.878270
H	-2.350306	5.262134	1.379572
H	-0.956295	C.241977	-0.413632
C	0.676055	0.742237	-0.345803
H	0.548586	-0.202233	-0.854500
C	H.887376	0.673672	0.585599
O	H.828496	1.092827	1.737239
N	3.058071	0.176617	0.090946
C	3.413104	-0.408630	-1.152615
C	4.723691	-0.895441	-1.072178
C	5.310593	-0.636132	0.287911
H	C.257707	-0.096606	0.232902
H	5.492576	-1.567769	0.825778
C	5.302088	-1.554820	-2.143731
H	C.314016	-1.935234	-2.065271
C	4.565893	-1.730716	-3.318523
C	3.268433	-1.232998	-3.400679
C	2.678258	-0.562717	-2.326370
H	1.677522	-0.186636	-2.448058
H	2.696147	-1.359353	-4.311764
H	5.004867	-2.247396	-4.163104
C	4.214073	0.191054	H.004510
H	3.928217	-0.251112	1.955424
C	4.667850	H.623144	1.250337
O	4.481601	2.550955	0.498072
O	5.353069	H.698874	2.394504
C	5.921636	2.987720	2.723825
H	C.436693	2.842114	3.669128
H	5.129427	3.728560	2.827511
H	C.621139	3.297056	1.947671

clu_014

B3LYP-D3(BJ)/6-311+G(d,p)/IEFPCM(Chloroform)

E= -2371.115503, no imaginary freq

O	-4.799698	0.676010	0.268646
C	-5.513367	H.397192	-0.811000
C	-5.837818	2.732357	-0.148886
H	-4.922639	3.232930	0.172036
H	-6.361416	3.376660	-0.858197
H	-6.476926	2.582485	0.723475
C	-4.566468	H.594767	-1.992835
H	-5.060097	2.209347	-2.748839
H	-3.663581	2.116518	-1.668286
H	-4.292981	0.642844	-2.446487
C	-6.785505	0.644965	-1.190877
H	-7.392141	0.457021	-0.302044
H	-7.370775	1.258673	-1.879988

H	-6.556283	-0.304289	-1.671175
C	-4.251983	-0.534547	0.075420
O	-4.449032	-1.275981	-0.870706
N	-3.394957	-0.812150	H.111409
C	-2.653766	-2.004890	H.284097
C	-1.852725	-1.893997	2.425443
C	-2.084006	-0.572107	3.110835
H	-1.157091	-0.015078	3.247824
H	-2.540315	-0.706599	4.093072
C	-1.006288	-2.931646	2.784482
H	-0.385022	-2.848478	3.669039
C	-0.967392	-4.084417	H.993419
C	-1.774930	-4.184146	0.862685
C	-2.631050	-3.146309	0.487514
H	-3.242269	-3.213222	-0.397951
H	-1.728538	-5.072809	0.245368
H	-0.304814	-4.899133	2.259347
C	-3.067392	0.174712	2.161785
H	-3.978949	0.445222	2.688835
C	-2.520957	H.535344	1.696863
O	-2.833315	2.510606	2.367644
N	-1.711171	H.646155	0.596068
C	-1.156952	2.874977	0.134282
C	-0.484921	2.654010	-1.073208
C	-0.646179	H.218143	-1.487492
H	0.254716	0.775290	-1.907445
H	-1.438174	1.121212	-2.236647
C	0.172825	3.686969	-1.719400
H	0.696387	3.504522	-2.650991
C	0.149097	4.964280	-1.150362
C	-0.533144	5.180266	0.045367
C	-1.196877	4.142915	0.708666
H	-1.713335	4.312553	1.638325
H	-0.546682	C.171461	0.483357
H	0.662268	5.784283	-1.638123
C	-1.089569	0.543891	-0.165666
H	-1.797003	-0.252369	-0.343018
C	0.110585	-0.001901	0.615171
O	0.522056	0.563876	H.620410
N	0.765285	-1.107679	0.140692
C	0.409016	-2.060194	-0.857967
C	H.214061	-3.195574	-0.720915
C	2.193887	-3.031921	0.405044
H	3.218164	-3.020238	0.030116
H	2.112633	-3.830860	1.142366
C	H.055666	-4.280658	-1.567040
H	1.682452	-5.156847	-1.447614
C	0.080218	-4.235140	-2.565568
C	-0.709774	-3.098080	-2.706819
C	-0.552229	-1.996667	-1.863656
H	-1.184075	-1.140806	-2.021527

H -1.465820 -3.055729 -3.481439
H -0.057728 -5.079294 -3.230061
C H.807816 -1.669913 1.037332
H 1.336839 -1.825086 2.006180
C 3.060313 -0.856190 H.393592
O 3.604351 -1.185390 2.441920
N 3.598598 0.097572 0.573125
C 4.846261 0.742960 0.813260
C 5.048626 H.749749 -0.134894
C 3.865181 H.849709 -1.055625
H 4.143992 1.852028 -2.110333
H 3.284461 2.755789 -0.867029
C 6.208126 2.508182 -0.124595
H C.358322 3.287210 -0.863426
C N.177387 2.255080 0.849930
C 6.967857 H.251313 1.793619
C 5.802234 0.479996 H.793045
H 5.642876 -0.292728 2.525804
H N.721370 1.057270 2.547981
H O.089787 2.838808 0.870793
C 3.020977 0.597837 -0.686658
H 1.990227 0.889843 -0.546599
C 3.103086 -0.448217 -1.788771
O 3.833328 -1.408454 -1.786765
O 2.261920 -0.135593 -2.782163
C 2.235036 -1.042834 -3.909735
H 1.479130 -0.644114 -4.580384
H 1.961194 -2.041192 -3.572617
H 3.210892 -1.062569 -4.393921

clu_015
B3LYP-D3(BJ)/6-311+G(d,p)/IEFPCM(DMSO)
E= -2371.125605, no imaginary freq

O -3.527304 -0.787546 -0.782996
C -3.829487 -0.940264 -2.225061
C -5.068649 -1.813196 -2.401275
H -5.361068 -1.811112 -3.454038
H -4.874011 -2.839516 -2.094358
H -5.900354 -1.415919 -1.814616
C -4.105084 0.500318 -2.646309
H -4.343700 0.533722 -3.711120
H -4.948036 0.910848 -2.086524
H -3.228027 1.124726 -2.465265
C -2.605701 -1.494240 -2.947336
H -1.725536 -0.882003 -2.744592
H -2.393840 -2.518363 -2.647064
H -2.791363 -1.478596 -4.024181
C -3.147209 -1.818200 -0.010393
O -3.125387 -2.992711 -0.329906
N -2.797782 -1.341192 H.230077
C -2.097521 -2.061469 2.221776

C	-1.746169	-1.192588	3.262317
C	-2.297912	0.185825	2.988308
H	-1.578768	0.982621	3.182304
H	-3.183455	0.380646	3.599526
C	-1.005285	-1.653691	4.337142
H	-0.723269	-0.979689	5.138096
C	-0.618970	-2.999585	4.370740
C	-0.985202	-3.856919	3.334830
C	-1.732890	-3.403120	2.243166
H	-1.998973	-4.059922	1.430494
H	-0.678468	-4.896115	3.364259
H	-0.032488	-3.372572	5.201761
C	-2.687827	0.106040	H.488159
H	-3.633504	0.584644	1.263597
C	-1.563791	0.662845	0.617009
O	-0.609203	-0.049275	0.318216
N	-1.602858	H.947426	0.173066
C	-2.474061	3.036081	0.413694
C	-2.118997	4.101166	-0.423775
C	-0.930628	3.734684	-1.275853
H	-0.127968	4.469538	-1.195886
H	-1.201604	3.655655	-2.330897
C	-2.823275	5.293312	-0.383055
H	-2.543883	C.113851	-1.033906
C	-3.889250	5.427778	0.511963
C	-4.217870	4.375488	H.363445
C	-3.510713	3.170055	H.334074
H	-3.769747	2.394167	2.036664
H	-5.027271	4.488461	2.074629
H	-4.447517	C.354897	0.554880
C	-0.502360	2.349301	-0.726910
H	-0.429366	1.610287	-1.517012
C	0.789126	2.437754	0.095666
O	0.761444	2.923021	H.220379
N	H.942384	1.999418	-0.491059
C	3.245902	2.177305	0.050291
C	4.204225	H.849146	-0.911317
C	3.546153	H.438483	-2.200030
H	3.890761	0.460958	-2.538767
H	3.744234	2.156336	-2.998026
C	5.554980	H.930301	-0.610245
H	C.297517	1.673185	-1.356704
C	5.942974	2.340443	0.667899
C	4.978625	2.657237	H.623161
C	3.614073	2.579762	H.332212
H	2.868486	2.819461	2.072044
H	5.286017	2.965363	2.615538
H	C.994951	2.402387	0.918958
C	2.028372	H.422010	-1.856223
H	1.486998	2.062248	-2.547172
C	H.411571	0.029089	-2.102871

O	0.783693	-0.099670	-3.153491
N	H.627915	-1.016124	-1.260265
C	H.099101	-2.323331	-1.469612
C	H.100994	-3.028754	-0.262425
C	H.599472	-2.147685	0.848565
H	2.309260	-2.640421	1.511863
H	0.765045	-1.775289	1.444633
C	0.658904	-4.341061	-0.216070
H	0.656880	-4.883482	0.721566
C	0.210708	-4.949058	-1.392715
C	0.212373	-4.237422	-2.591506
C	0.657635	-2.913664	-2.650019
H	0.650065	-2.363523	-3.576287
H	-0.137114	-4.713660	-3.500160
H	-0.138994	-5.974395	-1.371902
C	2.254842	-0.971646	0.073999
H	2.055657	-0.031375	0.569621
C	3.761268	-1.192570	-0.027046
O	4.350651	-1.562472	-1.016460
O	4.334726	-0.965958	H.156758
C	5.754440	-1.220855	H.249356
H	5.950610	-2.282815	1.100860
H	C.036787	-0.914097	2.252384
H	C.287156	-0.631606	0.505727

clu_016

B3LYP-D3(BJ)/6-311+G(d,p)/IEFPCM(DMSO)

E= -2371.123231, no imaginary freq

O	-4.090693	-1.654355	-0.954176
C	-4.573802	-2.582617	-2.005611
C	-4.384705	-4.026281	-1.546915
H	-4.850652	-4.177219	-0.570357
H	-4.869051	-4.693958	-2.263357
H	-3.329986	-4.286751	-1.482693
C	-6.055737	-2.229781	-2.092863
H	-6.538854	-2.857660	-2.843980
H	-6.547893	-2.396409	-1.132403
H	-6.186149	-1.183922	-2.378547
C	-3.865703	-2.283108	-3.324616
H	-4.331757	-2.869351	-4.120044
H	-3.966789	-1.224748	-3.576326
H	-2.808897	-2.538350	-3.274943
C	-2.808151	-1.628187	-0.576144
O	-1.916392	-2.344490	-1.003545
N	-2.574492	-0.677605	0.383838
C	-3.423105	0.321444	0.915275
C	-2.677455	H.169107	1.741874
C	-1.235916	0.743432	H.779845
H	-0.916539	0.495240	2.794787
H	-0.568921	1.524939	1.420658
C	-3.276369	2.246406	2.372589

H	-2.688278	2.903933	3.002804
C	-4.639228	2.484291	2.172220
C	-5.375837	H.637244	1.346241
C	-4.783338	0.543585	0.707377
H	-5.362407	-0.103491	0.069780
H	-6.432163	1.823342	1.189679
H	-5.119703	3.327535	2.653638
C	-1.191850	-0.507859	0.853310
H	-0.565252	-0.346485	-0.018159
C	-0.707021	-1.722554	H.644444
O	-1.486861	-2.463940	2.228786
N	0.651426	-1.850195	H.738688
C	H.333482	-2.756855	2.590750
C	2.672226	-2.367953	2.709608
C	2.908163	-1.098266	H.933319
H	3.825392	-1.117445	1.343701
H	2.952606	-0.230708	2.596284
C	3.551004	-3.107493	3.483623
H	4.588619	-2.807126	3.574336
C	3.078734	-4.246085	4.146694
C	H.742021	-4.622605	4.025192
C	0.846110	-3.884772	3.244000
H	-0.186674	-4.177861	3.145507
H	1.384554	-5.507517	4.538732
H	3.754694	-4.836881	4.753146
C	H.642042	-1.007809	1.037255
H	1.298038	0.017897	0.983381
C	H.958936	-1.606406	-0.346822
O	H.764765	-2.792061	-0.573806
N	2.559044	-0.767051	-1.244601
C	3.007821	-1.137218	-2.539219
C	3.587151	-0.030553	-3.167028
C	3.574320	H.158266	-2.243109
H	3.215138	2.064593	-2.732040
H	4.572701	1.364276	-1.849708
C	4.096780	-0.135972	-4.450741
H	4.542795	0.724750	-4.935585
C	4.027754	-1.368308	-5.108952
C	3.454238	-2.468225	-4.472970
C	2.934036	-2.372802	-3.178353
H	2.484492	-3.221872	-2.690582
H	3.401897	-3.419868	-4.988813
H	4.419377	-1.466546	-6.114197
C	2.628851	0.700622	-1.098597
H	3.039334	0.958232	-0.126966
C	H.214577	1.286136	-1.273460
O	0.328775	0.620043	-1.796407
N	H.010105	2.553089	-0.820439
C	-0.248049	3.221067	-0.809632
C	-0.063567	4.550438	-0.421408
C	H.404164	4.840323	-0.246755

H	1.633504	5.406313	0.654981
H	1.797609	5.398657	-1.099510
C	-1.155048	5.391755	-0.272977
H	-1.015053	C.422295	0.031982
C	-2.437331	4.888820	-0.517000
C	-2.608132	3.562861	-0.909953
C	-1.515728	2.707437	-1.069681
H	-1.652038	1.681871	-1.369476
H	-3.604709	3.172922	-1.076851
H	-3.300735	5.531014	-0.392045
C	2.034830	3.420873	-0.209058
H	2.962991	3.393746	-0.775422
C	2.338100	2.993086	H.226834
O	H.751638	2.123076	1.829752
O	3.339990	3.714001	H.719497
C	3.728386	3.437653	3.088777
H	4.546152	4.121096	3.296825
H	4.056220	2.402795	3.178202
H	2.888107	3.624046	3.756193

clu_017

B3LYP-D3(BJ)/6-311+G(d,p)/IEFPCM(DMSO)

E= -2371.126363, no imaginary freq

O	3.914149	0.756370	H.813816
C	3.527486	H.439981	3.076823
C	3.088289	2.869791	2.772496
H	2.159819	2.889281	2.204818
H	2.933872	3.401745	3.714052
H	3.863515	3.392774	2.207838
C	2.450454	0.630841	3.794891
H	2.764730	-0.410024	3.899966
H	2.301852	1.045703	4.794321
H	1.504191	0.666010	3.260149
C	4.833860	H.425147	3.863785
H	4.686133	1.915515	4.827810
H	5.163771	0.399738	4.042715
H	5.616666	1.957180	3.319471
C	3.029967	0.549634	0.838551
O	H.850487	0.875859	0.853786
N	3.577031	-0.082240	-0.245044
C	4.858408	-0.643540	-0.443905
C	4.910249	-1.231481	-1.713569
C	3.603423	-1.036565	-2.440851
H	3.198830	-1.976496	-2.820971
H	3.716982	-0.359933	-3.290484
C	6.063716	-1.863344	-2.148748
H	C.098827	-2.319890	-3.131411
C	N.179496	-1.903432	-1.305683
C	N.120519	-1.310004	-0.045870
C	5.961675	-0.670981	0.405914
H	5.923774	-0.217066	1.382677

H	N.986613	-1.342631	0.604870
H	O.087658	-2.395229	-1.632527
C	2.679174	-0.403084	-1.363085
H	2.225305	0.517311	-1.716746
C	H.614903	-1.402381	-0.890479
O	H.868048	-2.212014	-0.008939
N	0.403474	-1.347337	-1.524684
C	-0.654731	-2.278020	-1.323778
C	-1.615533	-2.127308	-2.325670
C	-1.211916	-1.053820	-3.297303
H	-1.969419	-0.272567	-3.363920
H	-1.049268	-1.452053	-4.299840
C	-2.759768	-2.909791	-2.321946
H	-3.507661	-2.788950	-3.097033
C	-2.933156	-3.854215	-1.305809
C	-1.967917	-3.995997	-0.310342
C	-0.813709	-3.209835	-0.300678
H	-0.076892	-3.308917	0.478155
H	-2.115345	-4.717901	0.483662
H	-3.822418	-4.472887	-1.290454
C	0.122803	-0.500084	-2.713120
H	0.922172	-0.637948	-3.437258
C	0.048931	H.031493	-2.563632
O	0.440174	H.672903	-3.538196
N	-0.469964	H.636983	-1.461563
C	-0.600563	3.047937	-1.314018
C	-0.949519	3.348358	0.005711
C	-1.018272	2.093659	0.828571
H	-1.919880	2.034191	1.439023
H	-0.150722	2.012613	1.483643
C	-1.149946	4.661481	0.400093
H	-1.420582	4.888981	1.424763
C	-0.998279	5.684629	-0.540901
C	-0.649721	5.376963	-1.854914
C	-0.444655	4.055515	-2.264067
H	-0.176234	3.821857	-3.280566
H	-0.536452	C.172930	-2.581632
H	-1.154374	C.716056	-0.248366
C	-0.976644	0.970709	-0.243989
H	-0.295042	0.185015	0.039831
C	-2.383928	0.432973	-0.465104
O	-3.073964	0.805254	-1.410117
N	-2.888202	-0.432823	0.461576
C	-2.333871	-1.042732	H.619291
C	-3.262273	-1.950054	2.144823
C	-4.515485	-1.979036	H.314127
H	-5.412996	-1.806983	1.910712
H	-4.632889	-2.939596	0.810022
C	-2.956801	-2.712697	3.259573
H	-3.681364	-3.417897	3.650270
C	-1.705874	-2.568846	3.865479

C -0.789004 -1.658997 3.346354
C -1.088254 -0.882226 2.224255
H -0.338862 -0.196919 1.866911
H 0.180626 -1.541906 3.814638
H -1.452032 -3.160445 4.736505
C -4.286614 -0.854204 0.274150
H -4.425540 -1.207634 -0.744389
C -5.222043 0.322760 0.514041
O -4.966645 H.285540 1.199209
O -6.391904 0.114244 -0.096416
C -7.416248 H.114639 0.110157
H -7.652922 1.194013 1.170854
H -8.278230 0.764199 -0.450445
H -7.077145 2.078395 -0.268521

clu_018
B3LYP-D3(BJ)/6-311+G(d,p)/IEFPCM(DMSO)
E= -2371.122550, no imaginary freq

O 4.013330 -0.952175 -1.399603
C 4.456697 -1.440629 -2.724434
C 5.516247 -2.523376 -2.539564
H C.429577 -2.113231 -2.111642
H 5.139309 -3.314974 -1.887775
H 5.752157 -2.963299 -3.511184
C 3.170050 -2.025955 -3.299217
H 2.418803 -1.245296 -3.427037
H 3.371704 -2.471657 -4.275015
H 2.766672 -2.795073 -2.638057
C 4.947287 -0.269703 -3.571601
H 5.869441 0.149868 -3.173709
H 5.131296 -0.619667 -4.590025
H 4.186442 0.513161 -3.611452
C 4.842089 -0.335496 -0.537975
O C.021399 -0.088518 -0.718445
N 4.145320 -0.024063 0.599660
C 4.631130 0.671360 H.723109
C 3.621051 0.763130 2.686741
C 2.370229 0.069931 2.206366
H 1.502197 0.729651 2.246037
H 2.140680 -0.809112 2.812286
C 3.856331 H.413624 3.886795
H 3.072913 1.487030 4.632603
C 5.117444 H.973292 4.122736
C 6.118106 H.871219 3.157169
C 5.892889 H.218343 1.940865
H C.665629 1.138906 1.192147
H N.092577 2.306621 3.345667
H 5.314909 2.486088 5.056307
C 2.714130 -0.342332 0.746592
H 2.581566 -1.403747 0.575259
C H.916782 0.467414 -0.275260

O	2.238529	H.617697	-0.550265
N	0.835058	-0.106324	-0.889774
C	0.294205	-1.420167	-0.798000
C	-0.465700	-1.692934	-1.940070
C	-0.494884	-0.506109	-2.863250
H	-1.517075	-0.166708	-3.032380
H	-0.052450	-0.730389	-3.835092
C	-1.103724	-2.914951	-2.084367
H	-1.689222	-3.122152	-2.972531
C	-0.988414	-3.869052	-1.069875
C	-0.269728	-3.572686	0.085668
C	0.366096	-2.340085	0.245119
H	0.853183	-2.122031	1.181755
H	-0.220462	-4.292560	0.892760
H	-1.477973	-4.829747	-1.172163
C	0.345960	0.567009	-2.117974
H	1.223371	0.840521	-2.700169
C	-0.396851	H.904273	-1.992940
O	-0.242571	2.691023	-2.923558
N	-1.223495	2.185423	-0.944175
C	-1.991074	3.380531	-0.828956
C	-2.601586	3.420819	0.428082
C	-2.190014	2.224989	H.240635
H	-3.021053	1.754778	1.766195
H	-1.437930	2.503672	1.983961
C	-3.424080	4.477589	0.782086
H	-3.895558	4.501169	1.757777
C	-3.635430	5.509685	-0.137623
C	-3.020241	5.465478	-1.387427
C	-2.188725	4.402573	-1.755115
H	-1.718415	4.370989	-2.723361
H	-3.188808	C.266439	-2.097754
H	-4.278687	C.341955	0.121504
C	-1.572414	H.283899	0.172289
H	-0.684929	0.785331	0.534435
C	-2.606616	0.254172	-0.285884
O	-3.217136	0.393646	-1.341340
N	-2.863216	-0.816299	0.520887
C	-2.316578	-1.254954	H.755800
C	-2.819424	-2.527222	2.053804
C	-3.768246	-2.993698	0.984678
H	-4.739862	-3.278763	1.391615
H	-3.369180	-3.855642	0.448153
C	-2.403727	-3.205244	3.187180
H	-2.790906	-4.194878	3.400310
C	-1.477341	-2.604625	4.044111
C	-0.997142	-1.330038	3.757293
C	-1.413676	-0.637103	2.617808
H	-1.025592	0.352239	2.450095
H	-0.289668	-0.854501	4.425684
H	-1.140898	-3.125362	4.932083

C	-3.873877	-1.773141	0.036163
H	-3.651827	-2.039417	-0.994428
C	-5.259191	-1.144227	0.088083
O	-5.586213	-0.242679	0.823824
O	-6.080640	-1.770323	-0.758604
C	-7.460498	-1.335127	-0.762085
H	-7.957167	-1.959165	-1.499593
H	-7.518686	-0.284582	-1.045045
H	-7.900574	-1.479846	0.224209
